# A deep transcriptomic resource for the copepod crustacean *Labidocera madurae*: A potential indicator species for assessing near shore ecosystem health

**DOI:** 10.1371/journal.pone.0186794

**Published:** 2017-10-24

**Authors:** Vittoria Roncalli, Andrew E. Christie, Stephanie A. Sommer, Matthew C. Cieslak, Daniel K. Hartline, Petra H. Lenz

**Affiliations:** Békésy Laboratory of Neurobiology, University of Hawai‘i at Mānoa, Honolulu, HI, United States of America; Stazione Zoologica Anton Dohrn, ITALY

## Abstract

Coral reef ecosystems of many sub-tropical and tropical marine coastal environments have suffered significant degradation from anthropogenic sources. Research to inform management strategies that mitigate stressors and promote a healthy ecosystem has focused on the ecology and physiology of coral reefs and associated organisms. Few studies focus on the surrounding pelagic communities, which are equally important to ecosystem function. Zooplankton, often dominated by small crustaceans such as copepods, is an important food source for invertebrates and fishes, especially larval fishes. The reef-associated zooplankton includes a sub-neustonic copepod family that could serve as an indicator species for the community. Here, we describe the generation of a *de novo* transcriptome for one such copepod, *Labidocera madurae*, a pontellid from an intensively-studied coral reef ecosystem, Kāne‘ohe Bay, Oahu, Hawai‘i. The transcriptome was assembled using high-throughput sequence data obtained from whole organisms. It comprised 211,002 unique transcripts, including 72,391 with coding regions. It was assessed for quality and completeness using multiple workflows. Bench-marking-universal-single-copy-orthologs (BUSCO) analysis identified transcripts for 88% of expected eukaryotic core proteins. Targeted gene-discovery analyses included searches for transcripts coding full-length “giant” proteins (>4,000 amino acids), proteins and splice variants of voltage-gated sodium channels, and proteins involved in the circadian signaling pathway. Four different reference transcriptomes were generated and compared for the detection of differential gene expression between copepodites and adult females; 6,229 genes were consistently identified as differentially expressed between the two regardless of reference. Automated bioinformatics analyses and targeted manual gene curation suggest that the *de novo* assembled *L*. *madurae* transcriptome is of high quality and completeness. This transcriptome provides a new resource for assessing the global physiological status of a planktonic species inhabiting a coral reef ecosystem that is subjected to multiple anthropogenic stressors. The workflows provide a template for generating and assessing transcriptomes in other non-model species.

## Introduction

Copepods are ubiquitous in aquatic and semi-aquatic habitats, living in marine, estuarine, freshwater and interstitial environments from the deepest ocean trenches to the top of mountain peaks [1]. *Labidocera madurae* is in the family Pontellidae, which are free-living surface dwelling planktonic copepods that are particularly abundant in coastal marine environments [2]. The genus *Labidocera* is a key member of oligotrophic waters surrounding coral reefs in the Pacific and Indian Oceans including Kāne‘ohe Bay, Oahu, Hawai‘i [3,4]. Kāne‘ohe Bay has a thriving coral reef community, which has shown significant resilience and the ability to recover from major environmental perturbations, including pollution, eutrophication, high temperatures, and low salinities [5–7]. It is also one of the best-studied coral reef ecosystems, and serves as a natural laboratory for experimental research on coral reef habitats [8–9]. Equally important are the pelagic regions that surround coral reefs, which serve both as a source of food and habitat for reef dwellers. Fishes, corals and other invertebrates have bi-phasic lifestyles: their larvae spend days to months in the plankton before settling nearshore, often within 100 m of their parents [10]. Furthermore, planktivorous reef-dwelling fishes and invertebrates depend on the abundant supply of zooplankton brought to them by currents [11–12]. Thus, the coral reef ecosystem includes both coral reef areas and the surrounding open water.

**Fig 1:**
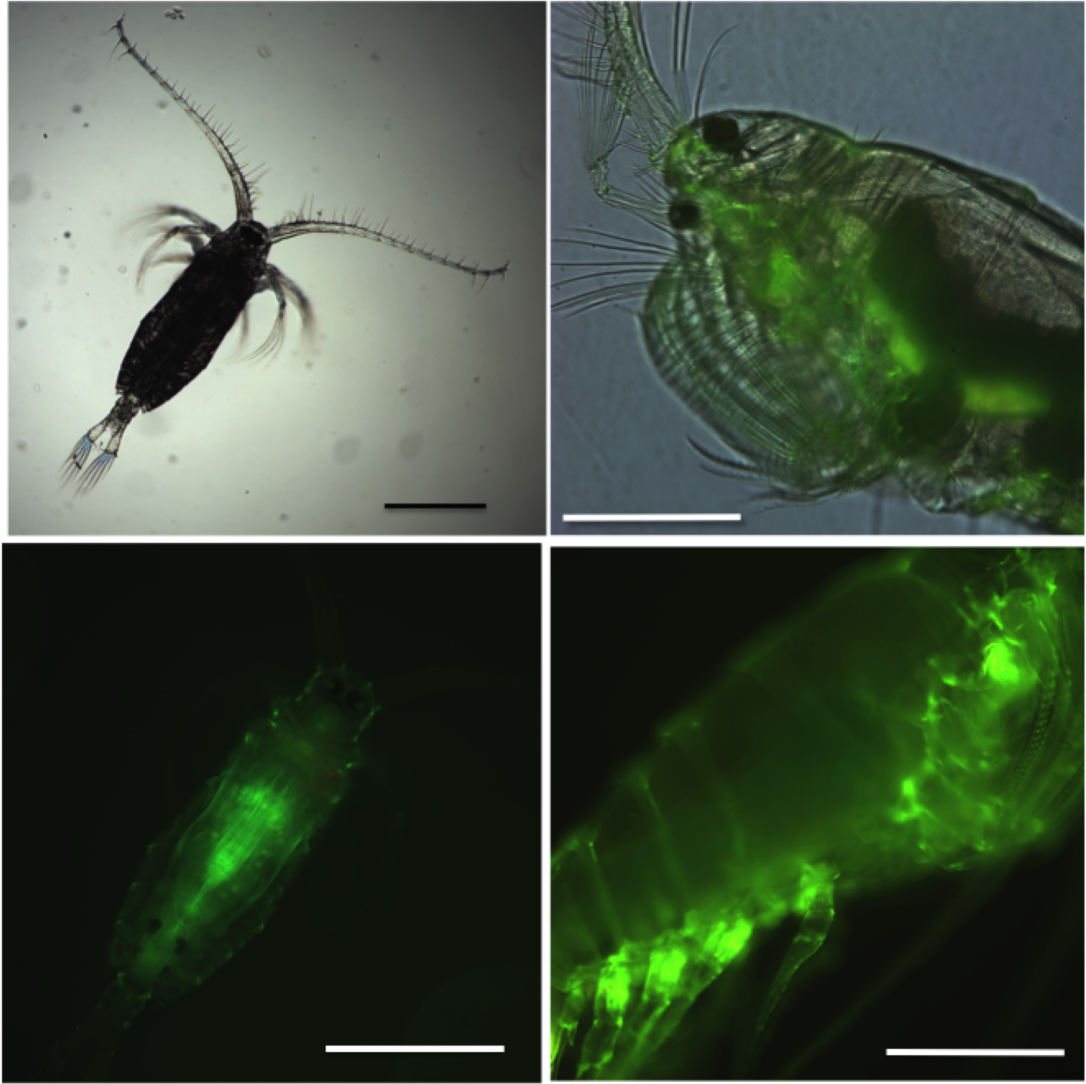
Light micrographs of *Labidocera madurae* copepodite (A, B) and adult female (C,D). (A) Copepodite stage CIII, dorsal view (magnification: 4x). (B) Same copepodite as in A under fluorescent light showing expression of green fluorescent protein (GFP) (magnification 10x). (C) lateral view of the anterior portion of an adult female showing one dorsal and the ventral ocelli, feeding appendages and GFP expression (magnification 10x). (D) Lateral view of the same individual as in C under fluorescent light showing GFP expression at the base of the swimming legs (magnification 10x). Scale bar: 0.5 mm.

The zooplankton community in Kāne‘ohe Bay is dominated by copepods including two cyclopoid species in the genus *Oithona*, two paracalanid species (*Bestiolina similis* and *Parvocalanus crassirostris*), and *L*. *madurae* [3]. Genetic barcoding indicates that while the *L*. *madurae* present in Kāne‘ohe Bay is genetically unique, it is clearly a member of the *L*. *madurae* species complex [3,4]. Because *L madurae* occurs throughout Kāne‘ohe Bay and its surrounding inshore waters, and it is moderately abundant year-round, it has the potential to be an indicator species for the pelagic regions of this estuarine system [13,14]. As one of the larger copepod species in Kāne‘ohe Bay (Fig 1), its physiology and behavior has been investigated [15–19]. However, *L*. *madurae* has been inaccessible to the genetic and genomic research tools that, applied to model organisms, have yielded so much insight into basic biology. As a group, copepods and other crustaceans are under-represented in the number of sequenced genomes and genomic resources. Thus, much of the basic understanding of the taxon potentially available from such resources is lacking. While there are several crustacean genome projects in progress (e.g., the water flea *Daphnia magna*, the copepods *Tigriopus californicus*, *Tigriopus kingsejongensis*, *Eurytemora affinis* and the amphipod *Hyalella azteca*), the genome of *Daphnia pulex* stands out as the only crustacean genome thus far completed, curated, fully annotated, and accessible through a searchable web portal (wFleaBase; http://wfleabase.org/) [20].

Transcriptomes can be reconstructed with high-throughput sequencing technologies. However, the quality of *de novo* assemblies is variable [21,22], and poor quality limits their usefulness in physiological and cellular studies that use gene expression profiles. Thus, the goal of this study was to generate a deep and high-quality *de novo* transcriptome for *L*. *madurae*. Furthermore, multiple workflows were used to provide complementary indicators for assessing its quality and depth. RNA was obtained from multiple developmental stages to increase the representation of transcripts, since a significant percentage of genes are silent in any particular stage [23]. Bioinformatics tools described in the Methods section were used to assemble and provide an initial evaluation of quality based on assembly, mapping and annotation statistics. This analysis was followed by targeted searches for transcripts encoding proteins of interest: green fluorescent proteins (GFP; Fig 1), the voltage-gated sodium channel (Na_V_), and the proteins involved in circadian signalling. All of the proteins are highly conserved across eukaryotes and possess stereotypical structural domains that were used to vet the completeness of the identified sequences.

## Materials and methods

### Sample preparation and RNA sequencing

Total RNA was obtained from two developmental stage groups of *L*. *madurae*: mix of copepodites (CIII to CV) and adult females (CVI) (S1 Table). All animals used here were collected in summer 2015 from central Kāneʻohe Bay (Hawaiʻi) (Lat: 21°4’N; Long: 157°7’W) using surface net tows with a 0.25 m diameter, 125-μm mesh plankton net. The field collection did not require any permits or approval and was performed by PHL and DKH using a personal watercraft. Zooplankton collections were immediately diluted into a bucket containing 5–10 L of seawater and returned to the laboratory. Adult female and copepodite *L*. *madurae* were sorted from samples under the microscope, rinsed in filtered seawater, transferred onto a sieve to remove excess seawater and either preserved in RNAlater (Ambion) (adult females) or prepared for immediate RNA extraction (copepodites). The copepodites were inspected for stage distribution prior to total RNA extraction. Three biological samples were obtained for each group with 5 to 6 pooled individual females and approximately 15 to 26 pooled copepodites for each replicate sample (S1 Table).

Total RNA was extracted using the QIAGEN RNeasy Plus Mini Kit (catalog # 74134) with Qiashredder (catalog # 79654) following the instructions of the manufacturer and stored in a -80°C freezer. For each sample, RNA concentration and quality were checked using an Agilent model 2100 Bioanalyzer (Agilent Technologies). Total RNA samples were shipped on dry ice to the Georgia Genomics Facility (University of Georgia, Athens, GA; dna.uga.edu) for library preparation and sequencing. Double-stranded cDNA libraries were prepared using the Kapa Stranded mRNA-seq kit (KK8420) following manufacturer’s instructions with a mean library insert size of 201–300 bp. Briefly, RNA samples were first purified with two oligo-dT selection (poly(A) enrichment using oligodT beds), and then fragmented and reverse transcribed into double-stranded complementary cDNA. Each sample was tagged with an indexed adapter and paired-end sequenced (151 bp, 300 cycles) using a High Output Flow Cell in a single lane using an Illumina NextSeq instrument (NextSeq 500) (S1 Table).

### *De novo* assembly and functional annotation

Prior to assembly, raw sequencing reads were assessed for quality using FASTQC (v1.0.0; Illumina Basespace Labs). The six RNA-Seq libraries were quality filtered using FASTQ Toolkit (v.2.0.0; Illumina Basespace Labs) by trimming the first nine bp, removing Illumina adapters (TruSeqLT universal primer) and low quality reads (“Phred” cutoff score ≥ 30), and setting the minimum read length to 50 bp. This led to the removal of an average of 11% of reads, leaving from 79 to 85 million reads per sample for the *de novo* assembly. The resulting reads from the six libraries were combined and assembled using Trinity (v. 2.0.6) [24] on the National Center for Genome Analysis Support’s (NCGAS; Indiana University, Bloomington, IN, USA) Mason Linux cluster. The initial parameters of Trinity were set to:–seqType fq–CPU 32–max_memory 200G –min_contig_length 300 –normalize_max_read_cov50. The minimum sequence length in the assembly was set to 300 bp. A summary of the assembly statistics was obtained using the script TrinityStat.pl (v2.0.6). Quality-filtered reads were mapped back to the reference using Bowtie2 software (v2.1.0) [25].

Functional annotation was performed in different steps. First, we predicted transcripts with coding regions (CDS) using TransDecoder (v3.0.0) with default settings (minimum open reading frame [ORF] length 100 amino acid and multiple ORFs per transcript) [24]. Then, all predicted transcripts with coding region were automatically annotated using a local BLAST webserver on a Beowulf cluster running the NCBI BLAST algorithm [26]. The BLASTx algorithm was used to search against the SwissProt protein database [27] (downloaded on 18^th^ September, 2015 from NCBI) employing a maximum E-value for annotation of 10^−3^. As a third step, the resulting BLAST annotations were mapped against the Gene Ontology (GO) and the Kyoto Encyclopedia of Genes and Genomes (KEGG) pathway database using UniProt [28]. The transcripts with GO terms were classified under three categories: biological process, molecular function and cellular component, which are hierarchically organized into levels. Lastly, "Bench-marking universal single-copy orthologs" (BUSCO) software (v1.22) was used to identify core genes: a set of single-copy genes highly conserved among eukaryotes and thus expected to be present in a complete assembly [29]. BUSCO analysis was performed using the Arthropoda dataset consisting of 2,675 single-copy orthologs.

### Transcriptome mining and confirmation of protein identification

In addition to the automated annotation step, a targeted approach was used to identify and vet transcripts encoding Na_V_s and GFPs and circadian signalling system proteins. The complete assembled transcriptome was downloaded to a local Beowulf cluster running the NCBI BLAST algorithm [26], and queried using known protein sequences for transcripts encoding putative homologs of the target groups (GFP: the copepod *Pontella mimocerami;* Na_V_ and circadian system: fruit fly *Drosophila melanogaster*, monarch butterfly *Danaus plexippus* or the copepod *Calanus finmarchicus*).

Nucleotide sequences with low E-value hits were translated (TranSeq or ExPASy) and then aligned (MAFFT, (v7) [30]) with and checked for homology to the query protein (typically better than 50% identity). Each deduced protein was used to query the NCBI non-redundant proteins (nr) to confirm the annotation. For Na_V_ channels, conserved regions were located in the MAFFT alignments with the *C*. *finmarchicus* predicted proteins as a check on the identification. Protein identity was confirmed by the presence of the characteristic four amino acid (DEKA) selectivity filter [31]. For GFP proteins, the online program Pfam (v 29.0) [32] was used to check for the presence of a GFP domain. BLAST searches for transcripts encoding putative circadian signaling system proteins including those for core clock, clock-associated, clock input pathway and clock output pathway proteins [33–35]. The circadian proteins were identified as “full-length” if they exhibit a functional signal sequence (including a “start” methionine) and were flanked on their C-terminus end by a stop codon, while “partial” proteins either lacked a start methionine (referred to as C-terminal partial proteins), or a stop codon (referred to as N-terminal partial proteins), or both of these features (referred to as internal fragment proteins). Next, each predicted *L*. *madurae* protein was used as the input query in a BLAST search of the annotated *Drosophila* protein dataset present in FlyBase (v FB2016_05) [36], except for CRY1 and CRY2. For these two proteins, the extant *D*. *plexippus* protein dataset present in GenBank was used for the reciprocal BLAST. The arthropod protein most similar to each *L*. *madurae* sequence was subsequently determined by conducting a BLAST search of the non-redundant arthropod protein dataset (taxid:6656) curated at NCBI. Finally, protein structural motifs were analyzed for each of the *L*. *madurae* proteins using the online program Pfam (v 29.0) [32]. This manual annotation was compared with the KEGG pathway annotation (map0471).

A key member of the circadian system is pigment dispersing hormone (PDH), which undergoes post-translational modification. Thus, the mature structures of *L*. *madurae* PDH and several other peptides derived from the PDH preprohormone were deduced using a workflow employed previously for peptide structural prediction in crustaceans, including copepods [37,38]. Specifically, the precursor protein in question was assessed for the presence of a signal peptide using the online program SignalP 4.1 [39]; the D-cutoff values of SignalP 4.1 were set to “Sensitive”. Prohormone cleavage sites were identified based on homology to known arthropod PDH preprohormone processing schemes. Carboxyl (C)-terminal amidation at glycine residues were predicted by homology to known peptide isoforms, while the sulfation state of tyrosine residues was predicted using the online program “Sulfinator” [40].

### Reference transcriptomes and differential gene expression

Four different transcriptomes were constructed and assessed for differential gene expression between copepodites and adult females. In addition to the full transcriptome (“Full”) consisting of 211,002 transcripts, three “reference” transcriptomes were generated and searched: 1) “Trinity predicted genes”, consisting of unique TR#_c#_g# and the longest “*i*”; 2) “Full-CDS”, which included only transcripts with predicted coding regions using TransDecoder [24] on the full transcriptome; 3) “Pred. genes-CDS”, which was derived from the Trinity predicted gene transcriptome and included only transcripts with predicted coding regions using TransDecoder [24].

Mapping and statistical analysis were performed using the pipeline described for “Differential expression using a Trinity assembly” [24] employing kallisto for mapping and edgeR for the statistical analysis. We compared these analyses to a second approach using Bowtie as the mapping program, followed by edgeR. Briefly, the quality filtered reads from the six RNA-Seq libraries were mapped against each reference transcriptome using either Bowtie (default settings; v. 2.0.6) [25] or kallisto (default settings; v.0.43.1) [41]. Each dataset generated by the mapping program was then tested for statistical significance using the BioConductor package edgeR [42]. As implemented by edgeR, prior to statistical testing, RNA-Seq libraries were normalized using the TMM methods (trimmed means of M values), followed by the removal of transcripts with expression levels below 1 count per million (1 cpm). Transcripts with a Benjamini-Hochberg corrected p-value <0.05 were considered differentially expressed (DEGs). Venny (v. 2.1) and BioVenn were used to generate Venn diagrams of the DEGs identified using kallisto and Bowtie [43,44]. Differential expression of the target genes was analyzed and compared across transcriptomes.

## Results and discussion

To date, the majority of publications describing *de novo* transcriptomes of calanoid copepods have targeted a single genus, *Calanus* [23, 45–48]. The individuals used in the current study are from the coastal region of Oahu, Hawai‘i: they belong to the *L*. *madurae* species complex [3,4]. Illumina sequencing of six libraries yielded 528 million paired-end reads ranging in length from 50 to 151 base pairs (bp) and over 92% of these reads were of high quality (Phred score ≥30). These reads were *de novo* assembled using the Trinity software package (see Methods)(Table 1). The first step in quality assessment was to generate the battery of standard statistical measures characterizing the results. The assembly produced 211,002 transcripts with an average length of 872 bp, a maximum of 23,836 bp and an N50 value of 1,184 bp (Table 1). It contained 153,604 “Trinity predicted genes” that is transcripts with unique “TR# | c#_g#” identifiers (Table 1). Of the “Trinity predicted genes”, the majority (127,025) were singletons (83%), with the remaining genes (26,579) possessing from two to 71 “Trinity predicted isoforms” (TR#|c#_g#_i#). This is similar to the percentage reported for *C*. *finmarchicus* [23].

**Table 1:** *De novo* assembly and annotation statistics. *Labidocera madurae* RNA-Seq data from six samples were combined, quality filtered and trimmed and assembled using Trinity software [24].

**Sequencing and Quality Filtering**				
	Raw reads (#)			528,000,341
	Sequencing yield (Mb)			89,510
	Trimmed and cleaned reads (#)			490,065,221
**Assembly**				
	Assembled transcripts (#)			211,002
	Trinity predicted genes (#)*			153,604
	Unique TR identifiers (#)*			89,545
	Minimum sequence length (bp)**			301
	Average contig length (bp)			872
	Longest contig length (bp)			23,836
	Total length of all sequence in assembly (bp)			184,023,017
	GC Content (%)			40.7
	N50 (bp)			1184
	N25 (bp)			2655
	N75 (bp)			538
	Mapped reads (#)			444,863,396
	Mapped reads (%)			90.8%
**Annotation of transcripts encoding proteins**				
	Transcripts with coding regions (CDS) (#)		TransDecoder	72,391
	Transcripts with BLAST hits (#)		SwissProt	62,980
	Transcripts with GO terms (#)		UniProt	60,097
	Transcripts with KEGG terms (#)		KEGG	57,912
	Core Eukaryotic Genes (#)		BUSCO	2,354
		Complete genes (%)***		76
		Complete duplicated (%)***		0.2
		Fragmented genes (%)***		11
		Missing genes (%)		12

* Trinity’s hierarchical nomenclature (“TR# | c#_g#_i#”) classifies assembled sequences by similarity. “TR#” corresponds to gene “families”; unique “TR# | c#_g#” corresponds to predicted “genes”.

** Minimum sequence length of > 300 bp was set as one of the assembly parameters

***“Complete” is defined as a gene with a predicted length that is within two standard deviations of the BUSCO group mean length that get annotation against the “Eukaryotes databases”. “Complete duplicate” indicates that multiple transcripts annotated to the same core gene such as transcripts with predicted isoforms. “Fragmented genes” refers to transcripts that encode partial proteins.

For the *L*. *madurae* assembly, the mapping rate was high, ranging from 88 to 92% for the six individual samples (Table 1, S1 Table), which is above the suggested cut-off at 80% mapping rate for a successful assembly. Ambiguous mapping, which was ~30% (31–37% of reads that aligned >1 time; S1 Table), is likely due to the large number of multiple isoforms assembled by Trinity.

**Fig 2:**
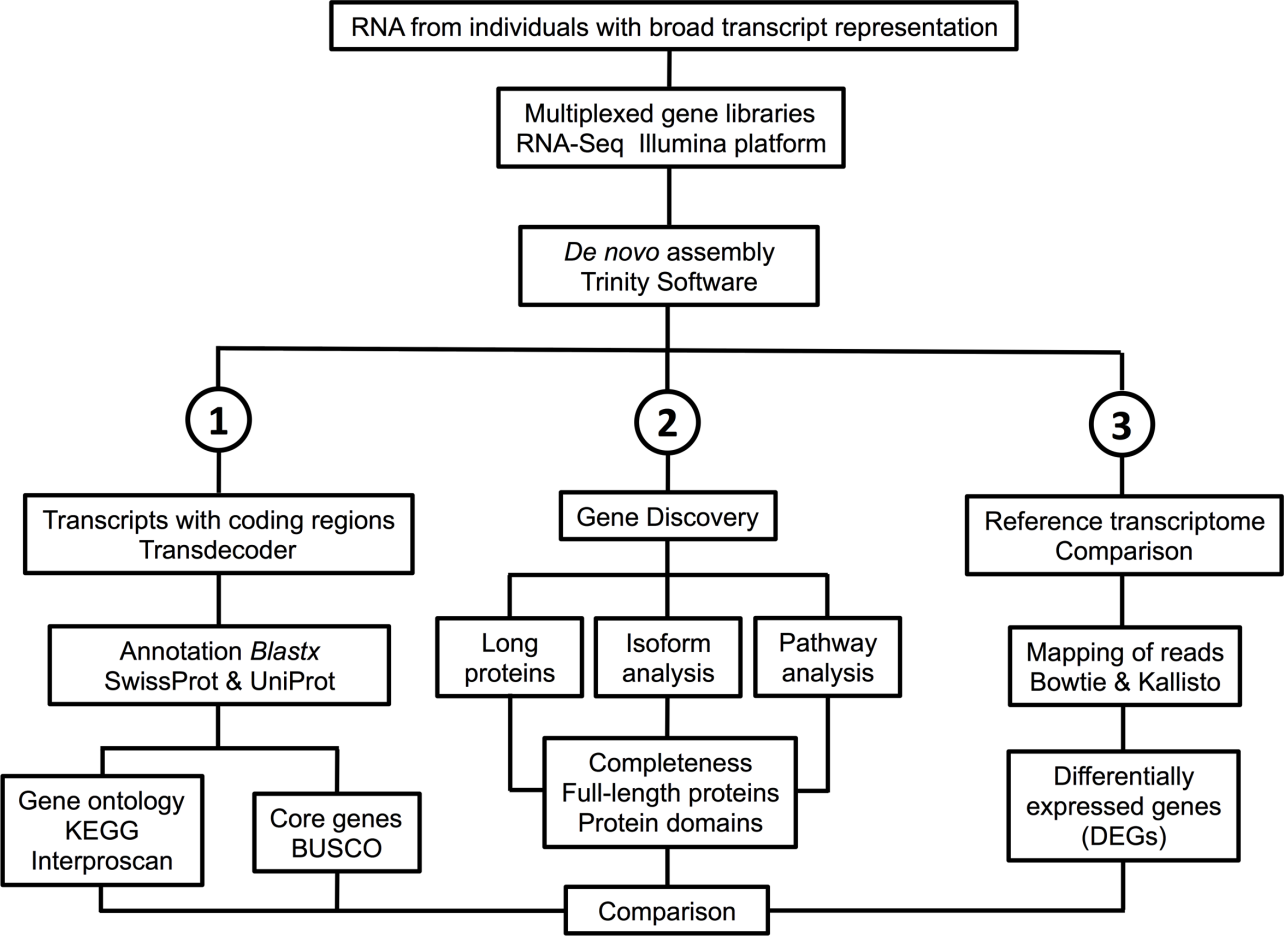
Diagram of the workflow used to generate the *de novo* transcriptome for *Labidocera madurae* and the three approaches used to test for completeness and quality of the assembly.

The complete *de novo* transcriptome containing 211,002 transcripts was used in three separate workflows to further assess the quality of the assembly (Fig 2, see methods). First, bioinformatic tools were used to identify transcripts with coding regions (CDS), which were then annotated against SwissProt, Gene Ontology and KEGG databases, followed by BUSCO analysis. Next, targeted protein discovery was focused on large conserved and complex proteins (“giant proteins” and Na_V_s), proteins of interest of this copepod group (GFPs and crystallins), and proteins members of a complex pathway (circadian signalling system). Finally, several approaches were tested for generating a representative reference transcriptome for gene expression studies.

### Functional annotation of the transcriptome

**Fig 3:**
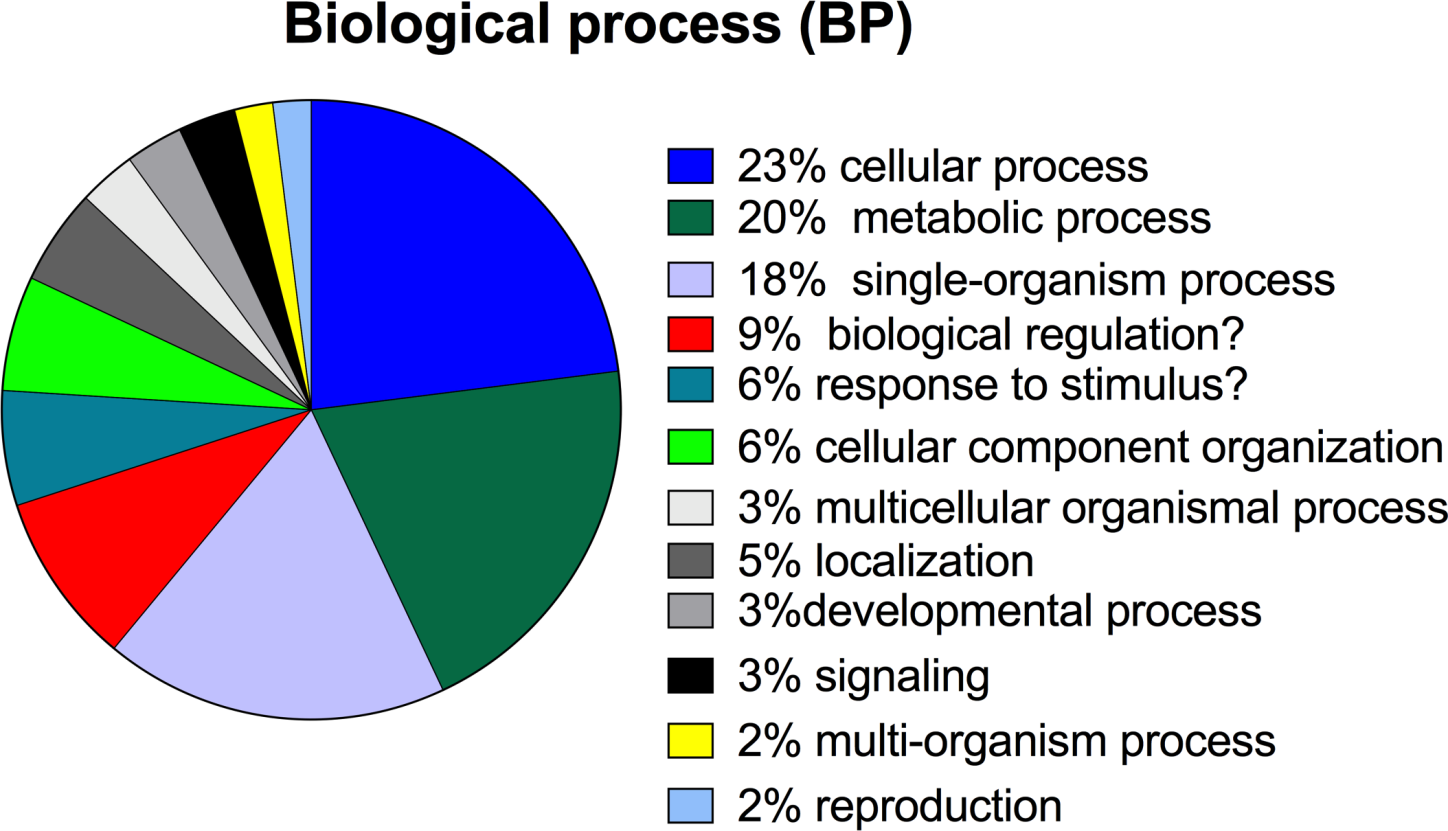
Biological processes represented in *L*. *madurae* transcriptome. Pie chart of the annotated transcripts including Gene Ontology (GO) terms belonging to the biological process (BP) category.

TransDecoder (see Methods) identified 72,391 transcripts with coding regions (CDS; length ≥ 100 amino acids) in the *de novo* assembly. Nearly 87% of the CDS retrieved significant hits with E-values of 10^−3^ or lower when blasted against the SwissProt database, and over 95% of these were further annotated with gene ontology terms (Table 1). Within the “biological process” category, *L*. *madurae* transcripts covered broadly conserved eukaryotic processes with “cellular process”, “metabolic process” and “single-organism process” representing more than 60% of the annotated transcripts (Fig 3). Eighty percent of transcripts with GO terms were annotated within the KEGG database (Table 1), indicating good coverage of transcripts encoding proteins/enzymes involved in lipid, amino acid and energy metabolism pathways (S1 Fig). BUSCO analysis identified 76% (2,036) complete orthologs of 2,679 core eukaryotic genes in the CDS with <1% of these genes present in more than one copy (duplicated). An additional 11% of fragmented core genes were found among the CDS, with only 12% of core genes missing completely (Table 1).

The assembly and annotation statistics of the *L*. *madurae de novo* transcriptome were compared with those of other non-model arthropods: three insect species and five other copepods (Table 2) [23, 47,49–53]. The number of assembled transcripts is quite variable across *de novo* transcriptomes with the number in the *L*. *madurae* transcriptome (~200K) being among the highest (Tables 1 and 2). The number of transcripts with coding regions is higher in copepods, including *L*. *madurae*, than that reported for the insect, *Lygus hesperus* (Western tarnished plant bug)[49]. Interestingly, the *L*. *madurae* annotation rates (87% of transcripts with coding regions) were higher than those reported in the other copepods which can in part be attributed to limiting annotation to protein encoding transcripts (Table 2). The number of predicted core proteins was similar across the transcriptomes with an approximate coverage of 80 to 90% based on the BUSCO analysis (Table 2). Overall, the annotation statistics suggests that the *L*. *madurae* transcriptome is at least as good in quality and depth as the others with which it was compared.

**Table 2:** Comparison of *de novo* transcriptomes generated for non-model arthropods.

		Hexapoda			Copepoda				
		Hemiptera			Calanoida		Cyclopoida	Harpacticoida	
		*Lygus hesperus*	*Cuerna arida*	*Graphocephala atropunctata*	*Calanus finmarchicus*	*Calanus sinicus*	*Paracyclopina nana*	*Tigriopus japonicus*	*Tigriopus kingsejongensis*
Sequencing platform		Illumina HiSeq	Illumina HiSeq	Illumina HiSeq	Illumina HiSeq	454 GS FLX	Illumina HiSeq	Illumina HiSeq	Illumina HiSeq
Transcripts (#)		22,022	91,830	97,830	206,041	31,591**	125,631	140,130	81,653
Minimum Length (bp)		297	224	224	301		201	201	224
Maximum Length (bp)		23,350	20,095	17,082	23,068	> 4,000	30,223	30,174	8,427
N50		2,610	1,560	1,692	1,418	873*	4,178	3,565	1,283
% mapping			88	95	89				
Transcripts with coding regions (CDS)		13,689			159,790		67,179	54,761	38,250
Transcripts with BLAST hits (#)		16,942			28,616	9,497	21,397	39,507	22,977
Transcripts with GO terms (#)		12,114			10,334			27,706	16,815
BUSCO									
	Complete (%)	74	68	66	79		72	81	72
	Duplicated (%)	33	26	24	20		0.2	0.4	3.5
	Fragmented (%)	13	17	19	8		5.7	6.9	10
	Missing (%)	17	14	13	12		21	11	17

* BUSCO analysis was performed in 2017 using publicly accessible NCBI “transcriptome shotgun assembly”. TSA data were first processed using transdecoder, followed by BUSCO (v.1.22) specifying the “Arthropoda” dataset, which included 2,675 core genes-analysis. TSA accession numbers: GAXK00000000 (*C*. *finmarchicus*), GCJT01000000 (*P*. *nana*), GCHA01000000 (*T*. *japonicus*), GDFW00000000 (*T*. *kingsejongensis*)

** # of transcripts given is the number of isotigs, N50 value is the isotig N50.*L*. *madurae de novo* assembly included a significant number of contigs (>100K), which lacked an open reading frame. Many of these non-coding sequences could belong to a class of transcripts called “long (>200 nucleotides) non-coding RNAs” (lncRNAs). While these sequences are often omitted from *de novo* transcriptomes, they are unlikely to be “assembly artifacts”.

The large number of putative lncRNA transcripts in *L*.*madurae* suggests that there may be more lncRNA loci in this crustacean than in *D*. *melanogaster* [54–55]. However, a shotgun assembly only produces predicted transcripts, and further analyses are needed to confirm which transcripts are indeed lncRNAs, as opposed to genes coding for very small proteins (<100 amino acids long), incomplete transcripts, or assembly artifacts (e.g. fragmented UTRs which have been found in this transcriptome).

### Searches of target genes based on automated annotation

#### “Giant” proteins

The presence of transcripts encoding “giant” proteins (those >4,000 amino acids) was used as an indicator of quality of the assembly. The *L*. *madurae* assembly included 23 transcripts that exceeded 15,000 bp in length. The lengths of these transcripts are comparable to those reported for six of the transcriptomes listed in Table 2. The majority of the long transcripts encoded “giant” proteins belonging to titin/connectin family, such as “twitchin”, and proteins involved in cellular architecture/cytoskeleton such as “nesprin”. Examples of long transcripts, all of which are predicted to be full-length and annotations are given in Table 3.

**Table 3:** Giant proteins. Four transcripts encoding “giant” proteins assembled using Trinity software in *Labidocera madurae* transcriptome. For each transcript, transcript length, predicted protein length, annotation name (NCBI), Accession No. of top blast hit (NCBI), E-value annotation (NCBI), protein family and protein function are listed.

	TR75346|c7_g2_i1	TR27483|c2_g1_i1	TR79107|c1_g1_i1	TR75290|c0_g1_i1
Transcript length (bp)	23,836	14,575	15,121	23,210
Predicted protein (aa)	7,112	4,555	4,683	7,737
Full/partial	Full	Full	Full	Partial
Annotation	Twitchin X20	TitinX21	Dynein heavy chain 5	Nesprin-1 X10
Accession No.	UNC22_CAEEL	dme:Dmel_CG1915	DYH5_MOUSE	SYNE1_HUMAN
E-vale annotation	0	0	0	0
Protein family	Titin family	Titin family	Dynein family	Nesprin family
Protein description	muscle contraction	muscle contraction	cytoskeletal motor protein	nuclear-cytoskeletal connections

#### Crystallins

An unusual feature of *Labidocera* and other pontellids is a sophisticated frontal eye structure [56, 57]. Unlike most copepods, the pontellid eye includes a clear lens, which requires structural proteins that are both stable and transparent. However not much is known about the structure of invertebrate lenses [58]. In vertebrates, the structural proteins of lenses include crystallins, which have been well characterized. A search of the *L*. *madurae* list of automated annotated transcripts identified 20 putative crystallins. Fifteen of these encode putative α-crystallins, with others encoding putative members of the β-crystallin (2), the γ-crystallin (1) and λ-crystallin (1) families (S2 Table). The β- and γ-crystallins, which form a partnership with α-crystallins, are the primary structural proteins of the vertebrate lens [59,60]. Thus, one or more of these transcripts might be involved in lens formation in *L*. *madurae*.

### Manual sequence annotation using targeted gene discovery

#### Green fluorescent proteins (GFPs)

Pontellids are well known for the presence of GFPs, which include some of the brightest GFPs currently known [61]. In *L*. *madurae*, GFPs are concentrated at the base of the appendages as seen in the side view of an adult female (Fig 1C and 1D). Three transcripts were found that putatively encode GFPs (S2 Table). Two of the predicted proteins, both full lengths, shared 90% amino acid identity with a pair of GFPs identified in a closely related species, *Pontella mimocerami* [61]. The third *L*. *madurae* GFP is most similar to a jellyfish (*Aequorea victoria*) GFP with which it shares 90% amino acid identity (S2 Table); this protein appears to represent a new class of copepod GFP. These putative transcripts encoding crystallins could serve as a starting point for any study investigating lens formation in copepods, specifically the pontellids, which possess modified naupliar eyes.

#### Large proteins with splice variants: voltage-gated sodium channels (Na_V_)

Large proteins that belong to families with closely-related members and which possess multiple splice sites or other regions of variation can be challenging to assemble and group dependably. One such protein family comprises the Na_V_s. In arthropods and in particular copepods *de novo* transcriptomes, incomplete or fragmented genes are common within this family (e.g. see publicly accessible transcriptomes in the following references: [23, 45, 48, 52] and NCBI Bioprojects PRJEB20069, PRJNA231234). Thus, as a stringent test of transcriptome quality, we assessed the assembly of the *L*. *madurae* Na_V_s proteins (Labma Na_V_s), comparing it with that from our previously published well-vetted transcriptome for *C*. *finmarchicus* [23, 38, 62,63]. We examined whether expectations were met in: 1) the number and completeness of predicted Na_V_ genes, identified by their expected characteristics (match statistics, conserved motifs, length); 2) the occurrence and nature of predicted splice variants; 3) how well Na_V_s were grouped into the Trinity hierarchy; 4) the occurrence and nature of irregularities (incorrect or incomplete sequences).

**Fig 4:**
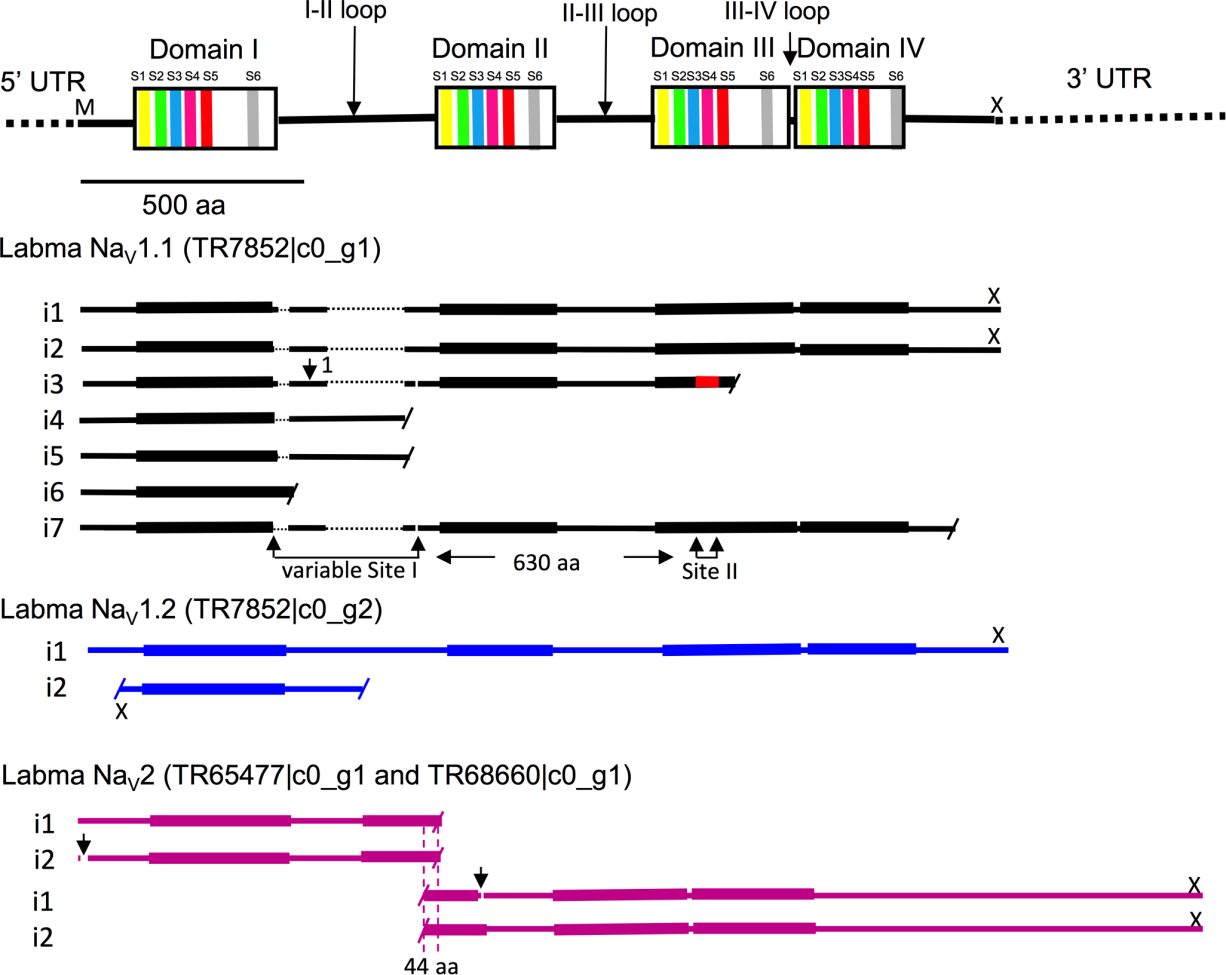
*Labidocera madurae* voltage-gated sodium channel sequences assembled by Trinity. Diagram at top shows the four well-conserved domains (DI-DIV) bridged by less-well-conserved loops. Conserved domains are depicted vertically expanded to show approximate locations of six trans-membrane α-helical segments (colored bands labeled S1, S2-S6). Sodium-selectivity of the Na_V_1 transcripts (but not Na_V_2) is confirmed by the occurrence of four characteristic amino acids (aspartic acid, glutamic acid, lysine and alanine [DEKA]) in specific locations termed the "P-loops" [31]. Coverage by variants of three putative genes, Labma Na_V_1.1 Labma Na_V_1.2 and Labma Na_V_2 indicated by bars labeled with the *i* number assigned by Trinity. For Labma Na_V_1.1, no one sequence possessed all of the pieces (putative exons), so the overall span across the diagram represents a manual reconstruction generated by including all of the pieces from the different *i*’s. Gaps in sequences are indicated by fine dotted lines. Identical 5' (504 nucleotide) UTRs for i1-i7 have been omitted, as have the identical 3' UTRs (1518 nucleotides) of i1 and i2. Within each gene, corresponding residues across different *i*’s were identical (reflected in the same coloration of the bars) in almost all cases, except for the splice variant indicated in red for Na_V_1.1 *i*3. Sequences representing partial predicted proteins not initiated by an M at the N-terminal or terminated by a stop codon (“X” above the bar) at the C-terminal are indicated with a short diagonal bar. Positions of the domains for Na_V_2 differ somewhat from those of Na_V_1 shown in the top diagram and are indicated by thickening of the bars. Two sites of putative splice variation (Site I and II) are indicated below the Na_V_1.1 diagram, and one non-optional segment within Site I is designated "1" (96aa). Arrows in the Na_V_2 diagram indicate short optional pieces (gaps in the horizontal bars), and the overlap region between the two pairs of isoforms of 44 identical amino acids (aa) is indicated.

Characteristics of Na_V_s expected to be present in an invertebrate transcriptome include occurrence of contigs from two families of orthologous genes, designated Na_V_1 and Na_V_2 [64]. However, in *L*. *madurae* three predicted gene families (TR#) were identified as Na_V_s by the automated annotation. This is one more than expected (Table 4). These had low E-values (<8e-156) and were identified either as *para* or 60E, the *D*. *melanogaster* designations for Na_V_1 and Na_V_2 respectively. Querying the full transcriptome with a well-vetted arthropod sodium-channel sequence from *D*. *melanogaster* (SwissProt SP3500) retrieved 13 sequences from the same three gene families with E-values < 1e-88. Sequences with the next higher E-values had features of voltage-gated calcium channels. The retrieved sequences are shown diagrammatically in Fig 4. ReBLASTing each of the Na_V_ contigs into Flybase returned either *para* or 60E (Table 4). To further resolve the identity of the contigs, they were used to query the *C*. *finmarchicus* transcriptome [23], retrieving top hits for 7 isoforms corresponding to Na_V_1.1 (TR7852|c0_g1), 2 corresponding to Na_V_1.2 (TR7852|c0_g2) and 4 in two TR groupings corresponding to the Na_V_2 gene (TR65477_c0_g1 and TR68660_c0_g1). The motifs expected of Na_V_s, shown diagrammatically at the top of Fig 4 (see caption for details), were validated through sequence alignment (MAFFT) and hence the various groups have been designated Labma Na_V_1.1, 1.2 and 2.

**Table 4:** *Labidocera madurae* (Labma) voltage-gated sodium channel transcripts/predicted proteins.

Transcript			Deduced protein					
Trinity ID number (Drome Na_V_1 hits)	Lengthnt	Drome E-value ^1^	*C*. *finmarchicus* top hit	Labma name	Length aa	Type	Calfi e-value	Flybase top hit ^2^
TR7852|c0_g1_i1	7686	0.0	GAXK01152315	Na_V_1.1	1888	F	0.0	para-PAL
TR7852|c0_g1_i2	7668	0.0	GAXK01152315	"	1882	F	0.0	para-PBA
TR7852|c0_g1_i3	4399	0.0	GAXK01152316	"	1292	N	0.0	para-PBA
TR7852|c0_g1_i4	2636	0.0	GAXK01042242	"	710	N	0.0	para-PBE
TR7852|c0_g1_i5	2654	0.0	GAXK01042242	"	716	N	0.0	para-PBH
TR7852|c0_g1_i6	1928	e-168	GAXK01042242	"	474	N	0.0	para-PBH
TR7852|c0_g1_i7	5858	0.0	GAXK01152315	"	1785	N	0.0	para-PBA
TR7852|c0_g2_i1	6765	0.0	GAXK01186590	Na_V_1.2	2069	F	0.0	para-PAL
TR7852|c0_g2_i2	1731	e-135	GAXK01121435	"	547	I	0.0	para-PBE
TR65477|c0_g1_i1	3165	7e-89	GAXK01056270	Na_V_2	817	N	0.0	NaCP60E-PJ
TR65477|c0_g1_i2	3220	7e-89	GAXK01056270	"	819	N	0.0	NaCP60E-PM
TR68660|c0_g0_i1	5266	0.0	GAXK01056270	"	1755	C	0.0	NaCP60E-PJ
TR68660|c0_g0_i2	5281	0.0	GAXK01056270	"	1759	C	0.0	NaCP60E-PI
TR25803|c0_g1_i1	457	- ^3^	GAXK01114023 GAXK01037398	Na_V_X ^4^	50	I	4e-09 2e-08	para-PX

^1^ Query sequence = *Drosophila melanogaster* canonical Na_V_1 sequence SwissProt P33500

^2^ Top BLASTp result from Flybase annotated proteins; "para" = Na_V_1; "NaCP60E" = Na_V_2

^3^ Original identification based on automated annotation

^4^ Sodium channel not fully characterized

The *Drosophila melanogaster* Na_V_1 sequence (sp|P{35500) *para* was used as a query in a tBLASTn probe of the *Labidocara madurae* 2015 transcriptome (column Drome e-value) The top hits (Trinity ID number column), with e-values < e-84, were translated into protein sequnces and reblasted using the tBLASTn tool against the *Calanus finmarchicus* Gulf of Maine transcriptome [23]. The top hits from that BLAST are indicated in the column "*C*. *finmarchicus* top hit,*"* with e-values given in the column "Calfi e." These are used to identify the protein (column "Labma name") using the correspondence of comp222993 and comp299307 with Na_V_1.1, comp44060 and comp233807 with Na_V_1.2, and comp428211 with Na_V_2.

Full-length proteins of the Na_V_ family are expected to be around 200 kD in size. Completeness of predicted proteins was verified for one or more contigs from each Labma Na_V_1 gene as well as from the single reconstructed Labma Na_V_2 gene (see below). Start and stop codons as well as 5' and 3' UTRs are present in all three. When all optional sequence segments (putative exons) are included, predicted proteins 2072 and 2069 amino acids long result for Labma Na_V_1.1 and Labma Na_V_1.2, respectively. These match the lengths predicted for corresponding genes of *C*. *finmarchicus* (2094 and 2079 respectively) [23], for *D*. *melanogaster* Na_V_1 (2131; UniProtKB P35500), and for human Na_V_1.1 (2009; UniProtKB P35498). Similarly, the 2533 residue length of the reconstructed Labma Na_V_2 was within 2% of that for *C*. *finmarchicus* (2485aa) and 10% of that for *D*. *melanogaster* (2821aa). Thus, three Na_V_ genes, with appropriate characteristics, are well assembled in the *L*. *madurae* transcriptome. Two or more sites of splice variation separated by more than a cDNA-insert-length of identical bridging sequence cannot be assembled reliably without additional information. Labma Na_V_1.1 has two sites with variant segments at opposite ends of the molecule. Site I is an N-terminal region of optional segments (putative exons; Fig 4); site II is an alternatively spliced segment nearer the C-terminal end. Both sites correspond to ones in Calfi Na_V_1.1 (Table 4). The two sites are separated by a minimum of 630 residues in *L*. *madurae*, well over a cDNA-insert-length (200–300 bp mean value), so the associations implied by the contigs assembled that include those two regions are unreliable. This does not imply a poorer quality of assembly compared with other paired-end assemblies of cDNA inserts of the same length: it is intrinsic to the shotgun approach. This caveat applies to four of the seven contigs of Labma Na_V_1.1 (Fig 4), but as well to the long contigs (18 in all) of Calfi Na_V_1.1 (see Fig 10 of Lenz et al [23]). Despite this ambiguity, the Labma Na_V_1.1 contigs gave solid evidence for the presence of four optional segments at Site I and one alternative segment at Site II, which is qualitatively similar to the pattern found in *C*. *finmarchicus*. No clear evidence for splice variants was found for Labma Na_V_1.2 (i2 is an anomalous fragment, possibly artifactual), the same being the case for Calfi Na_V_1.2. For Labma Na_V_2, the two members of each pair of fragments (TR65477 and TR68660) differ in the presence of "optional" segments in each, a feature not found in Calfi Na_V_2 (arrows in the Na_V_2 diagram of Fig 4). Thus, aside from this last case, the *L*. *madurae* transcriptome showed splicing features expected from the *C*. *finmarchicus* assembly. Most differences in the details (see below) are likely species differences.

Hierarchical transcript grouping by Trinity, as outlined in Methods, enables classifying assembled sequences into likely gene families, genes and isoforms. It performed well on the Labma Na_V_1 genes, separating them correctly into two genes nested within a single family. In contrast, transcripts for the same Calfi Na_V_1 genes are more broadly assigned, spanning four "Chrysalis components" (comps = gene proxies; Table 4)[24]. Reassembly of the *C*. *finmarchicus* transcriptome using Trinity 2.0.6 only reduced this number from four to three and failed to include them in the same gene family. Thus the *L*. *madurae* transcriptome is of higher quality in this respect than that of *C*. *finmarchicus*. On the other hand, a single transcript coded for Calfi Na_V_2, while Labma Na_V_2 was present as two fragments assigned to different Trinity (2.0.6) predicted gene families (Table 4). Still, these fragments had overlapping ends and could be amalgamated to form a full-length predicted protein with all of the expected properties. Thus the overall structure of the three Na_V_ genes was successfully assembled in the *L*. *madurae* transcriptome with about the same quality as for that of the *C*. *finmarchicus*.

Irregularities in the *L*. *madurae* assembly were of several types, described in more detail in S5 Fig. To summarize, the number of Labma Na_V_s assembled was smaller (three vs. six) than for *C*. *finmarchicus*. This is likely in part a species difference. Anomalous sequences of various origins were also noted. These include a short contig (TR25803|c0_g1_i1) that may represent an additional Labma Na_V_1 (Table 4) and a sequence with a frame-shift that is probably an error. In addition, several issues appear to have arisen from the ambiguity in assembling regions of variation bridged by segments with identical sequences that are longer than one cDNA-insert- length: 1) isoforms, especially within the Labma Na_V_1.1 gene, code for partial rather than full-length proteins (Table 4); 2) Calfi Na_V_1.1s have many more full-length contigs (18 vs 2) perhaps reflecting a greater leniency of Trinity 1.0 for matching variable regions; 3) genetic variability within the population may have increased the number of variable regions, possibly contributing to premature truncation of sequences.

Overall, the *L*. *madurae* transcriptome assembled Na_V_s as well as or better than that of *C*. *finmarchicus* [23]. However, it highlighted the limitations inherent in matching variant segments separated by stretches of identical sequence longer than a cDNA-insert-length.

#### Key regulatory pathways: circadian signaling system

The number of full-length circadian signaling system proteins deduced from *Labidocera* assembly supports the conclusion that this transcriptome is of high quality. Twenty-one protein families [65–68] were searched for and putative homologs were identified in the *L*. *madurae* assembly (Table 5), with the proteins encoded by the identified transcripts predicted (S3 Table), and vetted via reciprocal BLAST searches (S4 Table and S5 Table) and protein structural motif analysis (S6 Table). The protein families included: 1) the core clock proteins clock (CLK): cryptochrome 2 (CRY2), cycle (CYC), period (PER) and timeless (TIM); 2) the clock-associated proteins: casein kinase II α (CKII α), casein kinase IIß (CKIIß), clockwork orange (CWO), doubletime (DBT), jetlag (JET), PAR-domain protein 1 (PDP1), protein phosphatase 1 (PP1), protein phosphatase (PP2A) catalytic subunit microtubule star (MTS), PP2A regulatory subunit twins (TWS), PP2A regulatory subunit widerborst (WDB), shaggy (SGG), supernumerary limbs (SLIMB) and vrille (VRI); 3)the clock input pathway protein cryptochrome 1 (CRY1); and 4) the putative clock output pathway proteins: pigment dispersing hormone (PDH) and pigment dispersing hormone receptor (PDHR).

**Table 5:** Putative *Labidocera madurae* (Labma) circadian signaling system transcripts/proteins identified via *in silico* transcriptome mining.

Circadian signaling system protein		Transcript/protein identifications				
		Transcript		Deduced protein		
Clock component	Family	Trinity identification number	Length*	Name	Length^+^	Type
Core clock	Clock (CLK)	TR80374|c0_g1_i1	1944	Labma-CLK	590	N
	Cryptochrome 2 (CRY2)	TR24805|c1_g1_i4	3157	Labma-CRY2	799	F
		TR24805|c1_g1_i12	5006	Labma-CRY2	799	F
		TR24805|c1_g1_i11	3036	Labma-CRY2	799	F
		TR24805|c1_g1_i10	4023	Labma-CRY2	799	F
		TR24805|c1_g1_i9	3691	Labma-CRY2	799	F
		TR24805|c1_g1_i8	4784	Labma-CRY2	799	F
		TR24805|c1_g1_i7	4837	Labma-CRY2	799	F
		TR24805|c1_g1_i6	5012	Labma-CRY2	799	F
		TR24805|c1_g1_i5	2978	Labma-CRY2	799	F
		TR24805|c1_g1_i3	3658	Labma-CRY2	799	F
		TR24805|c1_g1_i2	3049	Labma-CRY2	799	F
		TR24805|c1_g1_i1	3007	Labma-CRY2	799	F
	Cycle (CYC)	TR40651|c0_g1_i4	3926	Labma-CYC-v1	706	F
		TR40651|c0_g1_i1	4000	Labma-CYC-v1	706	F
		TR40651|c0_g1_i3	3982	Labma-CYC-v2a	700	F
		TR40651|c0_g1_i5	3908	Labma-CYC-v2b	700	F
		TR40651|c0_g1_i2	2278	Labma-CYC-v3	669	F
		TR40651|c0_g1_i7	3688	Labma-CYC-v4	663	F
		TR40651|c0_g1_i6	3614	Labma-CYC-v4	663	F
	Period (PER)	TR32117|c1_g1_i2	4925	Labma-PER-v1	1409	F
		TR32117|c1_g1_i1	4913	Labma-PER-v2	1405	F
	Timeless (TIM)	TR9084|c2_g1_i4	5887	Labma-TIM-v1	1173	F
		TR9084|c2_g1_i3	5875	Labma-TIM-v2	1169	F
		TR9084|c2_g1_i2	5851	Labma-TIM-v3	1161	F
		TR9084|c2_g1_i1	5839	Labma-TIM-v4	1157	F
Clock-associated	Casein kinase IIα (CKIIα)	TR16899|c1_g1_i1	2279	Labma-CKIIα	375	F
	Casein kinase IIβ (CKIIβ)	TR61463|c0_g1_i1	1281	Labma-CKIIβ	217	F
	Clockwork orange (CWO)	TR54681|c0_g1_i3	4432	Labma-CWO-v1	617	F
		TR54681|c0_g1_i2	4422	Labma-CWO-v1	617	F
		TR54681|c0_g1_i1	4404	Labma-CWO-v2	611	F
	Doubletime (DBT)	TR25584|c0_g3_i1	2273	Labma-DBT-I	312	F
		TR13652|c3_g1_i1	5782	Labma-DBT-II-v1	609	F
		TR13652|c3_g1_i2	5141	Labma-DBT-II-v2	586	F
		TR84098|c0_g1_i2	4145	Labma-DBT-III-v1	413	F
		TR84098|c0_g1_i1	6085	Labma-DBT-III-v1	413	F
		TR84098|c0_g1_i4	6288	Labma-DBT-III-v2	407	F
		TR84098|c0_g1_i3	4348	Labma-DBT-III-v2	407	F
	Jetlag (JET)	TR56999|c0_g1_i3	2307	Labma-JET	291	F
		TR56999|c0_g1_i2	2681	Labma-JET	291	F
		TR56999|c0_g1_i1	2293	Labma-JET	291	F
	Par domain protein 1 (PDP1)	TR26154|c2_g1_i2	1714	Labma-PDP1-I-v1	252	F
		TR26154|c2_g1_i1	1686	Labma-PDP1-I-v2	243	F
		TR81334|c0_g4_i2	1078	Labma-PDP1-II	266	F
		TR81334|c0_g4_i1	2886	Labma-PDP1-II	266	F
		TR85690|c1_g2_i3	2036	Labma-PDP1-III	329	F
		TR85690|c1_g2_i2	1955	Labma-PDP1-III	329	F
		TR85690|c1_g2_i1	2002	Labma-PDP1-III	329	F
		TR40313|c4_g1_i2	2359	Labma-PDP1-IV	312	F
		TR40313|c4_g1_i1	2324	Labma-PDP1-IV	312	F
	Protein phosphatase 1 (PP1)	TR8331|c4_g1_i1	1820	Labma-PP1-I	328	F
		TR44262|c1_g1_i1	3263	Labma-PP1-II	340	F
		TR58187|c0_g1_i1	3191	Labma-PP1-III	316	F
		TR43009|c0_g1_i1	2414	Labma-PP1-IV	468	F
	Protein phosphatase 2A (PP2A)–Microtubule star (MTS)	TR69087|c4_g1_i1	2162	Labma-MTS-I	311	F
		TR6003|c0_g1_i1	1742	Labma-MTS-II	350	F
	PP2A –Twins (TWS)	TR47276|c5_g1_i1	3687	Labma-TWS-I	445	F
		TR55093|c0_g1_i1	4446	Labma-TWS-II	534	F
	PP2A –Widerborst (WDB)	TR25971|c2_g2_i2	2441	Labma-WDB-v1	481	F
		TR25971|c2_g2_i1	2337	Labma-WDB-v2	465	F
	Shaggy (SGG)	TR76551|c2_g2_i2	3218	Labma-SGG-I	411	F
		TR76551|c2_g2_i1	3190	Labma-SGG-I	411	F
		TR80377|c0_g1_i2	5696	Labma-SGG-II-v1	600	F
		TR80377|c0_g1_i1	5675	Labma-SGG-II-v2	593	F
	Supernumerary limbs (SLIMB)	TR55609|c6_g1_i2	3676	Labma-SLIMB-v1	547	F
		TR55609|c6_g1_i1	3662	Labma-SLIMB-v2	546	F
	Vrille (VRI)	TR41378|c1_g1_i2	2296	Labma-VRI	457	F
		TR41378|c1_g1_i1	2339	Labma-VRI	457	F
Clock input	Cryptochrome 1 (CRY1)	TR53226|c0_g1_i1	2585	Labma-CRY1	531	F
Clock output	Pigment dispersing hormone (PDH)	TR22949|c0_g1_i2	731	Labma-prepro-PDH-v1	136	F
		TR22949|c0_g1_i1	701	Labma-prepro-PDH-v2	126	F
	PDH receptor (PDHR)	TR69493|c0_g1_i1	1635	Labma-PDHR	428	C

*Length in nucleotides.

^+^Length in amino acids.

Protein type abbreviations: F, full-length protein; N, amino (N)-terminal partial protein; C, carboxyl (C)-terminal partial protein.

Proteins used as queries in tblastn searches: CLK, *Drosophila melanogaster* CLK (**Accession No.** **AAC62234**); CRY2, *Danaus plexippus* CRY2 (**Accession No.** **ABA62409**); CYC, *D*. *melanogaster* CYC (**Accession No.** **AAF49107**); PER, *D*. *melanogaster* PER, isoform A (**Accession No.** **AAF45804**); TIM, *D*. *melanogaster* TIM (**Accession No.** **AAC46920**); CKII α, *D*. *melanogaster* CKIIα, isoform A (**Accession No.** **AAN11415**); CKIIß, *D*. *melanogaster* CKIIß, isoform B (**Accession No.** **AAF48093**); CWO, *D*. *melanogaster* CWO, isoform A (**Accession No.** **AAF54527**); DBT, *D*. *melanogaster* discs overgrown, isoform A (**Accession No.** **AAF57110**); JET, *D*. *melanogaster* JET, isoform A (**Accession No.** **AAF52178**); PDP1, *D*. *melanogaster* PDP1, isoform B (**Accession No.** **AAN12022**); PP1, *D*. *melanogaster* PP1 (**Accession No.** **CAA39821**); MTS, *D*. *melanogaster* MTS, isoform A (**Accession No.** **AAF52567**); TWS, *D*. *melanogaster* TWS, isoform A (**Accession No.** **AAF54498**); WDB, *D*. *melanogaster* WDB, isoform A (**Accession No.** **AAF56720**); SGG, *D*. *melanogaster* SGG, isoform A (**Accession No.** **AAN09082**); SLIMB, *D*. *melanogaster* SLIMB, isoform A (**Accession No.** **AAF55853**); VRI, *D*. *melanogaster* VRI, isoform A (**Accession No.** **AAF52237**); CRY1, *D*. *plexippus* CRY (**Accession No.** **AAX58599**); PDH, *Eucyclops serrulatus* Prepro-PDH I (deduced from **Accession No.** **GARW01021210**); PDHR, *D*. *melanogaster* pigment dispersing factor receptor, isoform A (**Accession No.** **AAF45788**).

**Fig 5:**
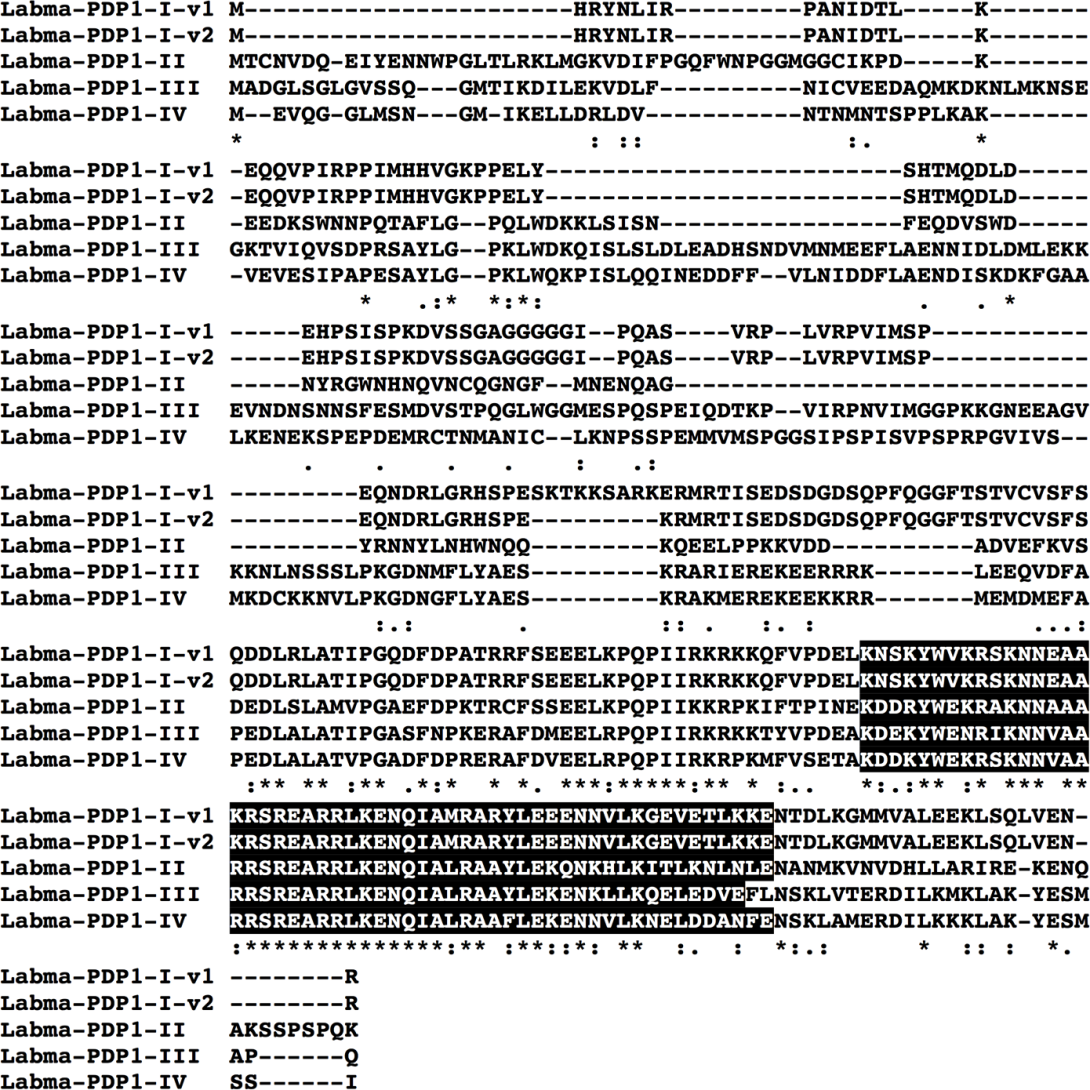
Alignment of five PDP1 protein sequences predicted from the *L*. *madurae de novo* transcriptome. Four genes were predicted (I-IV). The first two sequences (Labma-PDP1-I-v1 and Labma-PDP1-I-v2) are likely to be splice variants, since they are identical except for a 9 amino acid long indel.

Translation of the identified transcripts revealed that the vast majority encoded full-length proteins (Table 5, S3 Table), with just two encoding partial sequences (Table 5). For many protein groups, multiple variants, all likely derived from a common gene, were predicted. These variants were most likely derived from alternative splicing, as well as single nucleotide polymorphisms (*e*.*g*., the five CYC variants shown in S2 Fig). In addition, for a number of groups, proteins derived from multiple genes were identified (*e*.*g*., the four distinct PP1s shown in S3 Fig). PDP1 was represented with four predicted genes, one with a splice variant, as shown in Fig 5. While parts of the molecule were very conserved, there were significant differences between the predicted proteins, which may reflect diversity in function. In the case of the CRY2 protein, 12 distinct transcripts were identified, and while they differed in length (Table 5), the predicted proteins were all identical. These transcripts differed in the two untranslated regions (5'UTR and 3'UTR), which may be related to differential processing and/or tissue-specific expression.

In addition to vetting the completeness/quality of the *L*. *madurae* transcriptome, the mining of this resource for circadian protein-encoding transcripts has shed light on the clock system of this species, and for that matter, those of crustaceans in general. The large suite of proteins predicted from the *Labidocera* transcriptome (Table 5), include, among others, the canonical core clock proteins CLK, CYC, PER and TIM, all showing significant homology to those of *D*. *melanogaster* (S4 Table). They possess structural domains consistent with their fruit fly homologs, domains required for normal function (S1 Fig). Moreover, putative *L*. *madurae* homologs of both CRY1 and CRY2 were identified (Table 5), a finding that suggests that the *Labidocera* circadian system is organized more similarly to the “ancestral-type” clock proposed for lepidopteran/mosquito species than to that of *D*. *melanogaster* [66]. Specifically, CRY2, which is missing in *Drosophila*, but participates in the core clock itself, is likely to be a repressor of CLK-CYC-mediated transcription, while CRY1 functions as a photoreceptor, putatively providing photic input to the core clock. This result is consistent with the “ancestral-type” circadian systems described in other crustaceans that have been examined via genome/transcriptome analyses [33–35, 69], suggesting that this type of clock organization is broadly conserved within members of this arthropod subphylum.

The mining of the *Labidocera* transcriptome resulted in the discovery of the first PDP1s from a member of the Copepoda. The results suggest the presence of multiple genes from several protein families: DBT (three genes), PDP1 (four genes), PP1 (four genes), MTS (two genes), TWS (two genes) and SGG (two genes). No members of PDP1 had been identified previously from either *C*. *finmarchicus* or *T*. *californicus* [33,34]. The identification of the *L*. *madurae* PDP1 genes allowed for the revisitation of the *C*. *finmarchicus* and *T*. *californicus* transcriptomes for putative homologs. Using the *Labidocera* PDP1 predicted proteins as queries, related proteins have now been discovered in these two copepod species (A. E. Christie, unpublished). Moreover, mining of the assembly led to the prediction of a novel isoform of PDH, NSEMLHILRSMPKDMGKIIRNamide, which is just the second member of this peptide family identified from a copepod [37], a peptide that may serve as an output signal from the *Labidocera* clock for controlling its physiology and behavior.

**Fig 6:**
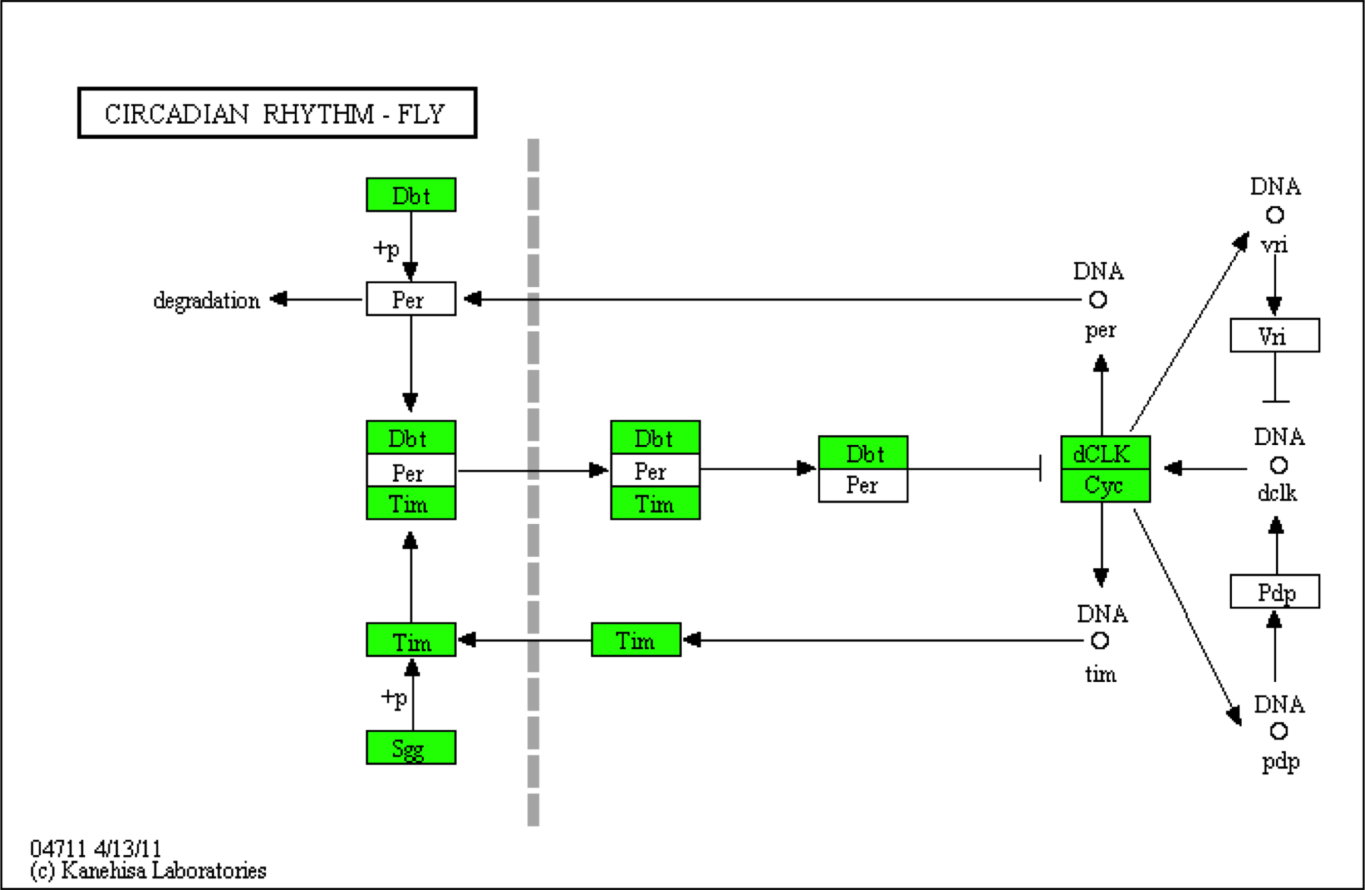
Predicted gene mapping to the circadian rhythm pathway obtained through KEGG annotation. Circadian rhythm pathway shown represents a map for *Drosophila melanogaster* (map04711). Highlighted boxes (green) represent *L*. *madurae* transcripts with coding regions (CDS) automatically annotated against the Kyoto Encyclopedia of Genes and Genomes (KEGG). PER, VRI, PDP1 were not identified by the automated annotation (white boxes).

Comparison between the results from the targeted gene discovery workflow with the results from the automated annotation is shown in Fig 6. The circadian system pathway retrieved from the KEGG database (map0471) resulted in the identification of five of the eight expected genes (Fig 6). The automated annotation programs failed to identify VRI, PDP1 and PER among the *L*. *madurae* transcripts with coding regions (CDS). These results underscore the value of targeted gene discovery in combination with the automated bioinformatics tools to obtain a complete annotation for a *de novo* transcriptome.

### Reference transcriptome analysis

#### Identification of differentially expressed genes between *L*. *madurae* developmental stages

The generation of a transcriptome that provides robust results for gene expression profiling is key for application to physiological ecology. While sequenced and annotated genomes are used as reference in model species, *de novo* assembled transcriptomes, in combination with bioinformatic tools for annotation and statistical testing, provide a powerful alternative. However, for a transcriptome of a non-model species to be used as an alternative for a genome, it needs to be of high quality and complete. Here, we compare four strategies for obtaining a reference for read mapping and identification of differentially expressed genes (DEGs). While the full transcriptome (211,002 transcripts) is optimal for targeted gene discovery, including the identification of genetic variants (i.e., splice variants, indels, SNPs), it also generates a large percentage of ambiguous mapping that could affect statistical testing. In addition to the full transcriptome (“Full”), we generated three alternative “reference” transcriptomes from the “Full” assembly by: 1) selecting the longest transcript for Trinity predicted genes (unique TR#_c#_g#; “Pred. genes”); 2) selecting only transcripts with coding regions (CDS) (“Full-CDS”); and 3) selecting only transcripts with coding regions (CDS) from the “Trinity predicted genes” transcriptome (“Pred. genes-CDS”).

Table 6 shows the effects of applying these filters. The number of transcripts decreased from 211K to 45K in the smallest “reference”. Nevertheless, the four transcriptomes were comparable with respect to the number of core eukaryotic proteins, which declined only by 3% between the full and the Trinity-predicted “unique” gene transcriptomes (“Pred. gene”, “Pred. gene-CDS”). With the exception of the full transcriptome, the number of duplicated genes (genes with more then one copy) was low (< 0.5%). The percentage of mapped reads using Bowtie decreased from 91% to 68% between the Full and Pred. genes-CDS references Furthermore, the three derived reference transcriptomes had fewer ambiguous reads than the full transcriptome, and the “unique gene” approach led to the lowest number of reads mapped more than once (14% and 6% for “Pred. genes” and “Pred. genes-CDS”, respectively).

**Table 6:** Comparison across four possible reference transcriptomes generated from the *de novo* assembly for gene expression studies. Reference transcriptomes—“Full”: complete *de novo* Trinity assembly; “Pred. genes”: retained a single (longest) isoform each Trinity-defined unique genes; “Full-CDS”: *de novo* Trinity assembly filtered using TransDecoder with only transcripts with predicted coding regions retained; “Pred. genes-CDS”: “Pred. genes” transcriptome filtered using TransDecoder with only transcripts with predicted coding regions retained. Number of transcripts, Bowtie mapping statistics and BUSCO analysis is given for each reference. Differential gene expression results include the number of transcripts that were included in the statistical analysis (expression level: > 1 cpm) and number of identified differentially expressed genes (DEGS) using either Bowtie or kallisto software as the mapping program.

		“Full”	“Pred. genes”	“Full-CDS”	“Pred. genes-CDS”
# Transcripts		211,002	153,604	72,391	45,090
MAPPING (%)*					
	Overall alignment	91	88.2	70	68
	Mapped >1 time	35	14	24	6
BUSCO (%)					
	Total	88	85	88	85
	Duplicated	20	0.4	0.2	0.5
GENE EXPRESSION					
*Bowtie*					
	# Transcripts >1cpm	38,237	29,951	28,674	19,437
	# DEGs	21,798	15,628	18,210	12,844
*Kallisto*					
	# Transcripts >1cpm	33,821	27,737	26,565	19,702
	# DEGs	13,138	13,137	12,050	11,017

*Mapping statistics are given as averages of six samples. Information for individual samples is provided in S1 Table.

**Fig 7:**
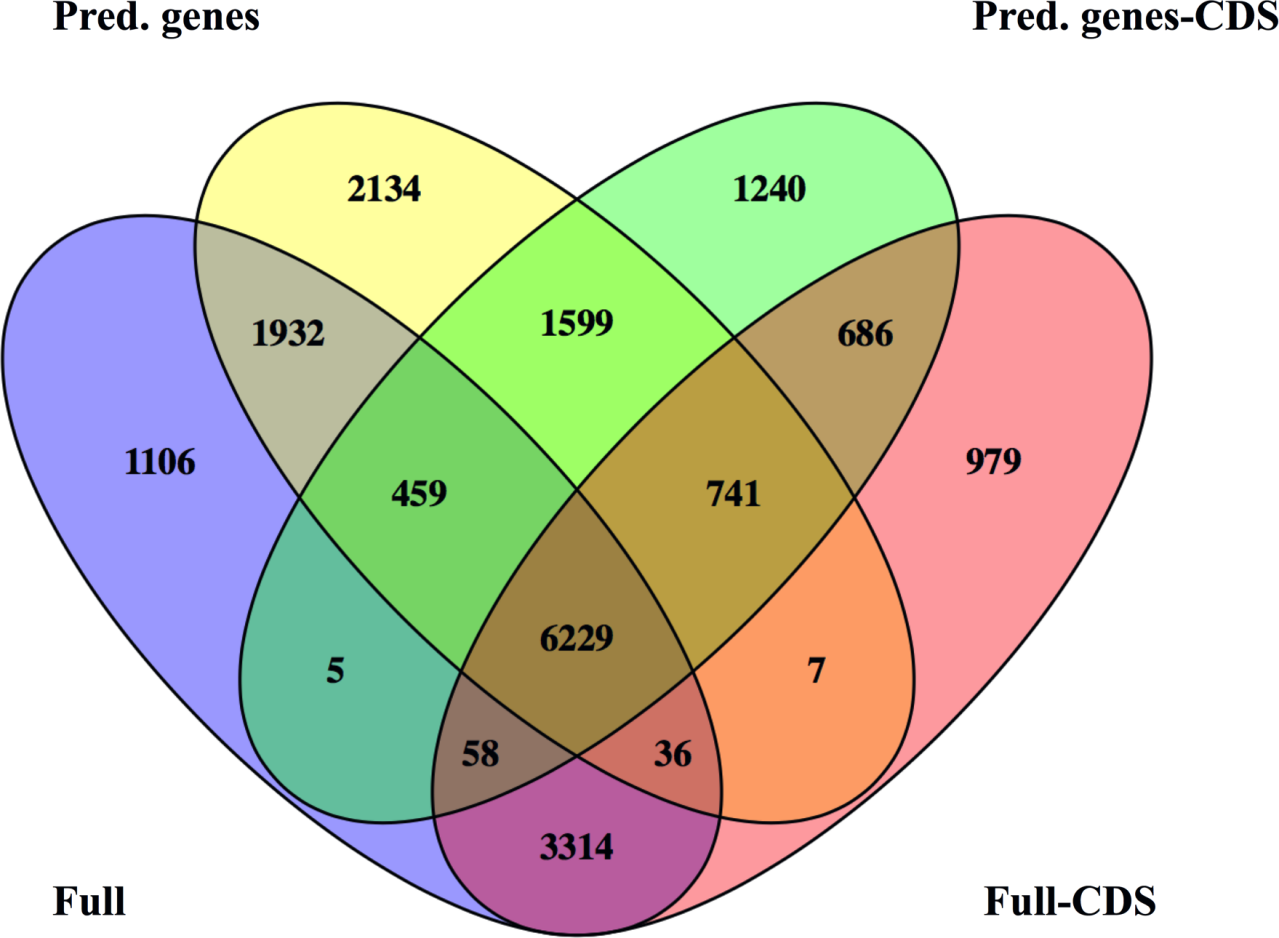
Non-proportional Venn diagram for the number of differentially expressed genes (DEGs) identified using four different transcriptomes as a reference for mapping of reads. The references transcriptomes are defined as: “Full” with 211K transcripts (purple), “Pred. genes” consisting of longest transcript for Trinity predicted genes (yellow), “Pred.genes-CDS” consisting of transcripts with coding regions (CDS) from the “Pred.genes” (green) and “Full-CDS” consisting of transcripts with coding regions (CDS) from “Full” (pink). Relative transcript abundance as determined using kallisto, and DEGs were identified by statistical analysis using edgeR with P<0.05 and false discovery rate (FDR) cutoff at 5%.

Differences among these potential “reference transcriptomes” were further evaluated by testing for differential gene expression between copepodites and adult females. Thus, we mapped reads to the four “reference transcriptomes” using two different bioinformatics tools (Bowtie and kallisto) followed by statistical testing to DEGs (edgeR). While the number of counts (= mapped reads) associated with each transcript is higher in Bowtie than in kallisto, this did not affect the number of transcripts tested for relative gene expression after applying the 1 cpm filter (Table 6). The number of DEGs identified by edgeR using counts generated by Bowtie varied by more than a factor of two among the references used. Nevertheless, 8,970 DEGs were shared among the four references (S4 Fig). In contrast, the number of DEGs identified with kallisto was similar for all four transcriptomes (Table 6), with 6,229 shared among all references (Fig 7). A comparison between Bowtie and kallisto of the shared DEGs identified 5,438 common DEGs (S4 Fig). The smallest reference transcriptome (“Pred. genes-CDS”) had best agreement between Bowtie and kallisto with 9,827 shared DEGs, which represented approximately 89% (kallisto) and 77% (Bowtie) of identified DEGs, which is not surprising given that this transcriptome had the smallest number of ambiguous reads (S4 Fig). In general, mapping by kallisto is more conservative, making it the preferred mapping program for the identification of DEGs, in particular in association with an assembly program like Trinity, which is designed to preserve isoform variants [24].

While the large number of shared DEGs regardless of mapping program or reference transcriptome (5,438 DEGs) was reassuring, there were still many of DEGs that were identified in one or two references but not the others as shown in the Venn diagram for DEGs generated from kallisto mapped reads (Fig 7). There was good agreement between the Full and Full-CDS (9,637 DEGs) and the Pred. genes and Pred. genes-CDS (9,028 DEGs; Fig 7).

#### Differential expression of transcripts identified through targeted gene discovery

To gain further insight into differences in expression, we examined expression results for the targeted genes identified in the previous sections (Tables 3, 4 and 5). For all investigated transcripts, expression rate was higher such that the transcripts did pass the 1 cpm filter and were considered for the statistical test. The transcripts encoding “giant” proteins were represented in all reference transcriptomes, and two transcripts, fibrillin-1 and nesprin-1, were consistently identified as differentially expressed (Table 7). Other target genes that contributed to the shared DEGs (6,229) included a Na_V_ (Labma1.2) and one transcript each of PER-v1, CWO-v1 and VRI (Table 7; Fig 7).

**Table 7:** Comparison among reference transcriptomes in the identification of differentially expressed genes (DEGs) between *L*. *madurae* copepodites and adult females among transcripts encoding for “giant” proteins, voltage-gated sodium channels and circadian system proteins. Transcripts were identified as DEGs using a Benjamini-Hochberg corrected p-value <0.05.

Target proteins						
		Transcript	Reference transcriptomes			
	Protein name	Trinity identification #	“Full”	“Pred.genes”	“Full-CDS”	“Pred.genes-CDS”
***“Giants”***						
	Twitchin X20	TR75346|c7_g2_i1	-	-	-	-
	Titin	TR27483|c2_g1_i1	-	-	-	-
	Dynein heavy chain5	TR79107|c1_g1_i1	-	-	-	-
	**Nesprin-1**	TR75290|c0_g1_i1	**YES**	**YES**	**YES**	**YES**
	Dystonin	TR39786|c3_g2_i1	-	-	-	-
	**Fibrillin-1**	TR81357|c0_g1_i1	**YES**	**YES**	**YES**	**YES**
	Nesprin-1	TR75299|c4_g1_i1	-	-	-	-
***Voltatge-gated sodium channel***						
	Na_V_1.1	TR7852|c0_g1_i1	-	-	-	-
		TR7852|c0_g1_i2	-	X	-	X
		TR7852|c0_g1_i3	**YES**	X	**YES**	X
		TR7852|c0_g1_i4	**YES**	X	**YES**	X
		TR7852|c0_g1_i5	-	X	-	X
		TR7852|c0_g1_i6	-	X	-	X
		TR7852|c0_g1_i7	-	X	-	X
	**Na**_**V**_**1.2**	TR7852|c0_g2_i1	**YES**	**YES**	**YES**	**YES**
		TR7852|c0_g2_i2	-	X	-	X
	Na_V_2	TR65477|c0_g1_i1	**-**	X	**-**	X
		TR65477|c0_g1_i2	-	-	**-**	**-**
		TR68660|c0_g0_i1	-	X	-	X
		TR68660|c0_g0_i2	-	-	-	-
		TR25803|c0_g1_i1	-	-	-	-
***Circadian system***						
Clock (CLK)	Labma-CLK	TR80374|c0_g1_i1	-	-	-	-
Cryptochrome 2 (CRY2)	Labma-CRY2	TR24805|c1_g1_i4	**YES**	X	**YES**	X
	Labma-CRY2	TR24805|c1_g1_i12	**-**	X	YES	X
	Labma-CRY2	TR24805|c1_g1_i11	**YES**	X	**YES**	X
	Labma-CRY2	TR24805|c1_g1_i10	-	X	-	X
	Labma-CRY2	TR24805|c1_g1_i9	**YES**	X	**YES**	X
	Labma-CRY2	TR24805|c1_g1_i8	**YES**	X	**YES**	X
	Labma-CRY2	TR24805|c1_g1_i7	**YES**	X	**YES**	X
	Labma-CRY2	TR24805|c1_g1_i6	-	**YES**	**-**	**YES**
	Labma-CRY2	TR24805|c1_g1_i5	-	X	-	X
	Labma-CRY2	TR24805|c1_g1_i3	**YES**	X	**YES**	X
	Labma-CRY2	TR24805|c1_g1_i2	**YES**	X	**YES**	X
	Labma-CRY2	TR24805|c1_g1_i1	**YES**	X	**YES**	X
Cycle (CYC)	Labma-CYC-v1	TR40651|c0_g1_i4	-	X	-	X
	Labma-CYC-v1	TR40651|c0_g1_i1	-	-	YES	-
	Labma-CYC-v2a	TR40651|c0_g1_i3	**YES**	X	**YES**	X
	Labma-CYC-v2b	TR40651|c0_g1_i5	**YES**	X	**YES**	X
	Labma-CYC-v3	TR40651|c0_g1_i2	**YES**	X	**YES**	X
	Labma-CYC-v4	TR40651|c0_g1_i7	-	X	-	X
	Labma-CYC-v4	TR40651|c0_g1_i6	-	X	-	X
**Period (PER)**	**Labma-PER-v1**	**TR32117|c1_g1_i2**	**YES**	**YES**	**YES**	**YES**
	Labma-PER-v2	TR32117|c1_g1_i1	**YES**	X	**YES**	X
Timeless (TIM)	Labma-TIM-v1	TR9084|c2_g1_i4	**YES**	**-**	**YES**	-
	Labma-TIM-v2	TR9084|c2_g1_i3	-	X	YES	X
	Labma-TIM-v3	TR9084|c2_g1_i2	**YES**	X	**YES**	X
	Labma-TIM-v4	TR9084|c2_g1_i1	**YES**	X	**YES**	X
Casein kinase IIα (CKIIα)	Labma-CKIIα	TR16899|c1_g1_i1	-	-	-	-
Casein kinase IIβ (CKIIβ)	Labma-CKIIβ	TR61463|c0_g1_i1	-	-	-	-
**Clockwork orange (CWO)**	**Labma-CWO-v1**	**TR54681|c0_g1_i3**	**YES**	**YES**	**YES**	**YES**
	Labma-CWO-v1	TR54681|c0_g1_i2	**YES**	X	**YES**	X
	Labma-CWO-v2	TR54681|c0_g1_i1	**YES**	X	**YES**	X
Doubletime (DBT)	Labma-DBT-I	TR25584|c0_g3_i1	-	-	-	-
	Labma-DBT-II-v1	TR13652|c3_g1_i1	-	**YES**	**-**	**YES**
	Labma-DBT-II-v2	TR13652|c3_g1_i2	-	X	-	X
	Labma-DBT-III-v1	TR84098|c0_g1_i2	-	X	-	X
	Labma-DBT-III-v1	TR84098|c0_g1_i1	-	X	-	X
	Labma-DBT-III-v2	TR84098|c0_g1_i4	-	-	-	-
	Labma-DBT-III-v2	TR84098|c0_g1_i3	-	X	-	X
Jetlag (JET)	Labma-JET	TR56999|c0_g1_i3	**YES**	X	**YES**	X
	Labma-JET	TR56999|c0_g1_i2	-	**YES**	**-**	**YES**
	Labma-JET	TR56999|c0_g1_i1	**YES**	X	**YES**	X
PAR-domain protein 1 (PDP1)	Labma-PDP1-I-v1	TR26154|c2_g1_i2	-	-	-	-
	Labma-PDP1-I-v2	TR26154|c2_g1_i1	-	-	-	-
	Labma-PDP1-II	TR81334|c0_g4_i2	-	X	-	X
	Labma-PDP1-II	TR81334|c0_g4_i1	-	**YES**	**-**	**YES**
	Labma-PDP1-III	TR85690|c1_g2_i3	-	-	-	-
	Labma-PDP1-III	TR85690|c1_g2_i2	-	X	-	X
	Labma-PDP1-III	TR85690|c1_g2_i1	**YES**	X	**YES**	X
	Labma-PDP1-IV	TR40313|c4_g1_i2	**-**	-	**-**	-
	Labma-PDP1-IV	TR40313|c4_g1_i1	**YES**	X	**YES**	X
Protein phosphatase 1 (PP1)	Labma-PP1-I	TR8331|c4_g1_i1	-	-	-	-
	Labma-PP1-II	TR44262|c1_g1_i1	-	-	-	-
	Labma-PP1-III	TR58187|c0_g1_i1	-	-	-	-
	Labma-PP1-IV	TR43009|c0_g1_i1	-	-	-	-
Protein phosphatase 2A (PP2A)–Microtubule star (MTS)	Labma-MTS-I	TR69087|c4_g1_i1	-	-	-	-
	Labma-MTS-II	TR6003|c0_g1_i1	-	-	-	-
PP2A –Twins (TWS)	Labma-TWS-I	TR47276|c5_g1_i1	-	-	YES	YES
	Labma-TWS-II	TR55093|c0_g1_i1	-	-	-	-
PP2A –Widerborst (WDB)	Labma-WDB-v1	TR25971|c2_g2_i2	-	-	-	-
	Labma-WDB-v2	TR25971|c2_g2_i1	-	X	-	X
Shaggy (SGG)	Labma-SGG-I	TR76551|c2_g2_i2	-	-	-	-
	Labma-SGG-I	TR76551|c2_g2_i1	-	X	-	X
	Labma-SGG-II-v1	TR80377|c0_g1_i2	-	-	-	-
	Labma-SGG-II-v2	TR69087|c4_g1_i1	-	-	-	-
Supernumerary limbs (SLIMB)	Labma-SLIMB-v1	TR55609|c6_g1_i2	-	-	-	-
	Labma-SLIMB-v2	TR55609|c6_g1_i1	-	X	-	X
Vrille (VRI)	Labma-VRI	TR41378|c1_g1_i2	-	X	-	X
	**Labma-VRI**	TR41378|c1_g1_i1	**YES**	**YES**	**YES**	**YES**
Cryptochrome 1 (CRY1)	Labma-CRY1	TR53226|c0_g1_i1	-	-	-	YES
Pigment dispersing hormone (PDH)	Labma-prepro-PDH-v1	TR22949|c0_g1_i2	-	**YES**	**-**	**YES**
	Labma-prepro-PDH-v2	TR22949|c0_g1_i1	-	X	-	X
PDH receptor (PDHR)	Labma-PDHR	TR69493|c0_g1_i1	-	-	-	-

Legend

- Transcript present in the reference transcriptome but not differentially expressed

YES Transcript present in the reference transcriptome and differentially expressed

X Transcript not present in the reference transcriptome

In bold Transcripts resulting differentially expressed in all 4 reference transcriptomes

The transcriptomes differed in the number of Na_V_ transcripts given the presence of isoforms. Thus, Na_V_1.1 and 1.2 had seven and two isoforms, respectively, in the Full and Full-CDS transcriptomes, while the two unique gene transcriptomes (Pred. genes and Pred. genes-CDS) had single transcripts representing each of these two genes (Table 7). Two isoforms (*i3*, *i4*) of the Na_V_1.1 transcripts were differentially expressed in the Full and Full-CDS transcriptomes, however, the single Na_V_1.1 in the other two transcriptomes was not among the DEGs (Table 7).

Several CRY2 isoforms were identified as differentially expressed in the Full and Full-CDS transcriptomes, but not in the Pred. genes and Pred. genes-CDS references (Table 7). In these two transcriptomes the “*i6*” isoform was among the DEGs (Table 7). A similar pattern was observed with JET–two out of three isoforms were among the DEGs, while the third isoform was identified as differentially expressed in the references with single isoforms (Table 7). While these are examples of disagreements between the references (Fig 7), the results are consistent in identifying at least one isoform of CRY2 and JET as differentially expressed.

Another pattern that occurred was the inclusion of multiple isoforms among the DEGs in both Full and Full-CDS transcriptomes, but not in the “unique gene” ones (e.g., CYC, Tim, PDP1-III and PDP1-IV). The reverse, differentially expressed according to the “unique gene” transcriptomes, but not the other two, occurred for transcripts of one doubletime (Labma-DBT-II), one PAR-domain protein 1 (Labma-PDP1-II) and pigment-dispersing hormone (Labma-PDH). Four DEGs were identified in a single reference (3 in Full-CDS and 1 in Pred. genes-CDS), while one DEG was shared between the two CDS-based reference transcriptomes (Table 7). In summary, comparing DEGs identified with four reference transcriptomes for the target genes indicated good concordance between the Full and the CDS-based transcriptomes (29/33) and the two “unique gene” references (Pred. genes and Pred. genes-CDS: 11/13). Agreement between all four transcriptomes regardless of isoform was observed in eight out of 13 genes. Inconsistent results across reference transcriptomes are typically associated with transcripts belonging to genes with multiple isoforms, such as those with predicted splice variants. Thus, independent of the method used for generating a reference transcriptome, it is important to assess the number of isoforms for each differentially expressed gene.

## Conclusions

High-throughput sequencing in combination with bioinformatics tools has made transcriptomic approaches accessible to non-model species, including those of ecological interest. Thus, transcriptomics can now be used to investigate the eco-physiology of key species within the context of life history strategies, population cycles and ecosystem dynamics. However, these types of studies, which involve gene expression profiling, depend on good reference transcriptomes. Application of multiple workflows to evaluate the quality and completeness of a transcriptome generated for the copepod *L*. *madurae* demonstrates that no single criterion is sufficient to assess a *de novo* assembly. High-throughput bioinformatics tools were used to identify transcripts with protein coding regions and provide annotations. Targeted gene discovery provided information on completeness of individual genes, identified possible sources of fragmentation, established predicted gene variants, and provided additional annotations. The analysis of four different strategies for generating a reference for gene expression studies suggest good agreement among references when a predicted gene assembled into a single isoform. However, many predicted genes include a multiplicity of isoforms, and when these are included in the reference they contribute to ambiguous mapping. Thus, one source of disagreement among transcriptomes in the identification of DEGs is related to which genes are regulated, and weather they are represented by multiple isoforms. The workflows developed in this study if used in a routine assessment of *de novo* transcriptomes would enhance the reliability of gene expression studies.

## Supporting information

S1 Fig Metabolic pathways represented in the *Labidocera madurae* transcriptome based on annotation using Kyoto Encyclopedia of Genes and Genomes (KEGG). Diagram in light purple is a map of 146 KEGG pathways that provide a generalized overview of global metabolism in eukaryotes. Metabolic compounds are identified by nodes, while the lines show enzymatic transformations. Highlighted blue lines and corresponding nodes represent the pathways that were annotated in the *L*. *madurae* transcriptome using SwissProt and KEGG pathway analysis. The KEGG map was customized using ipath2.
(PDF)

S2 Fig Comparison of variants of predicted cycle (Labma-CYC) proteins predicted from the *Labidocera madurae* transcriptome. Variants were aligned using MAFFT. In the line immediately below each sequence grouping, “*” indicates identical amino acid residues, while “:” and “.” denote amino acids that are similar in structure between sequences. In this figure, helix-loop-helix DNA-binding, PAS fold, and PAS domains identified by Pfam analyses are highlighted in yellow, light green, and light blue, respectively.
(DOCX)

S3 Fig Comparison of variants of predicted protein phosphatase 1 (Labma-P1) proteins predicted from the *Labidocera madurae* transcriptome. Variants were aligned using MAFFT. In the line immediately below each sequence grouping, “*” indicates identical amino acid residues, while “:” and “.” denote amino acids that are similar in structure between sequences. In this figure, serine-threonine protein phosphatase N-terminal and calcineurin-like phosphoesterase domains identified by Pfam analyses are highlighted in blue and red, respectively.
(PDF)

S4 Fig Venn diagrams of differentially expressed genes (DEGs) identified using two mapping software programs (Bowtie and kallisto) and different reference transcriptomes. The reference transcriptomes are defined as: “Full” with 211K transcripts, “Pred. genes” consisting of longest transcript for Trinity predicted genes, “Pred.genes-CDS” consisting of transcripts with coding regions (CDS) from the “Pred.genes” and “Full-CDS” consisting of transcripts with coding regions (CDS) from “Full”. A) Non-proportional Venn diagram comparing all four transcriptomes for the number of identified DEGs using Bowtie as the mapping program. “Full” transcriptome (purple), “Pred. genes” (yellow), “Pred.genes-CDS”(green) and “Full-CDS” (pink). B) Proportional Venn diagram comparing the DEGs that were shared among all four reference transcriptomes using either Bowtie (purple) or kallisto (green) as the mapping program. C) Proportional Venn diagram comparing DEGs identified using the smallest reference “Pred. genes-CDS” using either Bowtie (purple) or kallisto (green) as the mapping program. DEGs were separately identified for each transcriptome and mapping combination using edgeR set to P-value <0.05 and false discovery rate (FDR) cutoff of 5%.
(PDF)

S5 Fig Irregularities in the *L*. *madurae* assembly. (DOC)

S1 Table Summary of *Labidocera madurae* RNA-Seq and mapping analysis. For each stage three biological replicates were considered (R1, R2, R3). Number of pooled individuals (# ind), sequencing yields in number of reads (#) and number of megabases (Mb), are listed. For the mapping analysis overall alignment (%) and reads mapped > 1 time (%) are listed for each biological replicate.
(DOCX)

S2 Table Crystallins and green fluorescent proteins (GFP) in *L*. *madurae* transcriptome. A) Crystallins have been searched in the list of automated annotated transcripts. For each transcript, Annotation name (NCBI) E-vale annotation (SwissProt) and Aceesion No. (NCBI) of top Blast hit. B) Putative GFP identifies via *in silico* transcriptome mining. For each *L*. *madurae* transcripts, transcript and protein name and Top hit results (Top hit Accession No.,and BLAST E-value) and protein length (aa) are listed.
(XLSX)

S3 Table List of amino acid sequences for the *L*. *madurae* proteins involved in the circadian signaling system. I) Core clock proteins, II) Clock-associated proteins, III) Clock input pathway proteins and IV) Clock output pathway proteins.
(DOCX)

S4 Table Summary of BLAST analysis against FlyBase for the *L*. *madurae* transcript encoding proteins involved in the circadian signaling pathway. For each *L*. *madurae* transcripts, transcript and protein name and Top hit results (Top hit Accession No., BLAST Score and BLAST E-value) are listed.
(DOCX)

S5 Table Summary of BLAST analysis against NCBI GeneBank for the *L*. *madurae* transcript encoding proteins involved in the circadian signaling pathway. For each *L*. *madurae* transcripts, transcript name, BLAST results (Top hit Accession No., Species, Protein name, BLAST Score and BLAST E-value) are listed.
(DOCX)

S6 Table Summary of structural domains/regions predicted by Pfam in deduced *L*. *madurae* (Labma) circadian signaling system proteins. (DOCX)

## Abbreviations

BUSCO: bench-marking universal single-copy orthologs
CDS: transcripts with coding regions
cpm: counts per million
DEGs: differentially expressed genes
GO: gene ontology
Na_V_: voltage-gated sodium channel
aa: amino acid
KEGG: Kyoto Encyclopedia of Genes and Genomes
TSA: transcriptome shotgun assembly
Na_V_: voltage-gated sodium channels
GFP: green fluorescent proteins
CLK: Core clock proteins
CRY2: clock cryptochrome 2
CYC: cycle
PER: period
TIM: timeless
CKII α: casein kinaseII α
CKIIß: casein kinase IIß
CWO: clockwork orange
DBT: doubletime
JET: jetlag
PDP1: PAR-domain protein 1
PP1: protein phosphatase 1
PP2A: protein phosphatase
MTS: catalytic subunit microtubule star
TWS: PP2A regulatory subunit twins
WDB: PP2A regulatory subunit widerborst
SGG: shaggy
SLIMB: supernumerary limbs
VRI: vrille
CRY1: cryptochrome 1
PDH: pigment dispersing hormone
PDHR: pigment dispersing hormone receptor

## References

[1] Huys R and Boxshall GA. Copepod evolution. London: The Ray Society, Unwin Brothers; 1991.

[2] Mauchline J. The Biology of Calanoid Copepods. New York: Academic Press; 1998.

[3] Jungbluth MJ and Lenz PH. Copepod diversity in a subtropical bay based on a fragment of the mitochondrial COI gene. J Plankton Res. 2013;35 3:630–43. doi: 10.1093/Plankt/Fbt015

[4] Sanu VF, Nandan SB, Deepak J and Harikrishnan M. Molecular identification and systematic assessment of Labidocera madurae A. Scott, 1909 (calanoid copepod) from Lakshadweep Archipelago, southwest coast of India, based on mitochondrial COI gene sequences. Mar Biodivers. 2016;46 1:95–103.

[5] Smith SV, Kimmerer WJ, Laws EA, Brock RE, Walsh TW. Kaneohe Bay sewage diversion experiment: perspectives on ecosystem responses to nutritional perturbation. Pacific Science. 1981; 35 4:279–395.

[6] Jokiel PL, Hunter CL, Taguchi S, Watarai L. Ecological impact of a fresh-water “reef kill” in Kaneohe Bay, Oahu, Hawaii. Coral Reefs. 1993; 12 3:177–84.

[7] Bahr KD, Jokiel PL, Toonen RJ. The unnatural history of Kāne ‘ohe Bay: coral reef resilience in the face of centuries of anthropogenic impacts. PeerJ. 2015;3:e950 doi: 10.7717/peerj.950 26020007 10.7717/peerj.950 PMC4435448

[8] Hunter CL, Evans CW. Coral reefs in Kaneohe Bay, Hawaii: two centuries of western influence and two decades of data. Bull Mar Sci. 1995;57 2:501–15.

[9] Bahr KD, Jokiel PL, Rodgers KS. The 2014 coral bleaching and freshwater flood events in Kāneʻohe Bay, Hawaiʻi. PeerJ. 2015;3:e1136 doi: 10.7717/peerj.1136 26290792 10.7717/peerj.1136 PMC4540025

[10] Jones GP, Planes S, Thorrold SR. Coral reef fish larvae settle close to home. Current Biology. 2005;15 14:1314–8. doi: 10.1016/j.cub.2005.06.061 16051176 10.1016/j.cub.2005.06.061

[11] Hamner WM, Jones MS, Carleton JH, Hauri IR, Williams DM. Zooplankton, planktivorous fish, and water currents on a windward reef face: Great Barrier Reef, Australia. Bull Mar Sci. 1988;42 3:459–79.

[12] Hamner WM, Colin PL, Hamner PP. Export–import dynamics of zooplankton on a coral reef in Palau. MEPS. 2007;334:83–92.

[13] Leis JM. Nearshore distributional gradients of larval fish (15 taxa) and planktonic crustaceans (6 taxa) in Hawaii. Mar Biol. 1982;72 1:89–97.

[14] Hassett RP, Boehlert GW. Spatial and temporal distributions of copepods to leeward and windward of Oahu, Hawaiian Archipelago. Mar Biol. 1999;134(3):571–84.

[15] Yen J, Lenz PH, Gassie DV, Hartline DK. Mechanoreception in marine copepods: electrophysiological studies on the first antennae. J Plankton Res. 1992;14 4:495–512.

[16] Fields DM, Yen J. The escape behavior of marine copepods in response to a quantifiable fluid mechanical disturbance. J Plankton Res. 1997;19 9:1289–304.

[17] Hartline DK, Lenz PH, Herren CM. Physiological and behavioral studies of escape responses in calanoid copepods. Mar Freshwater Behav Physiol 1996;27 2–3:199–212.

[18] Lenz PH, Hartline DK, Davis AD. The need for speed. I. Fast reactions and myelinated axons in copepods. J Comp Physiol A: Neuroethol Sens Neural Behav Physiol. 2000;186 4:337–45. 10.1007/s003590050434 10798722

[19] Weatherby TM, Davis AD, Hartline DK, Lenz PH. The need for speed. II. Myelin in calanoid copepods. J Comp Physiol A: Neuroethol Sens Neural Behav Physiol. 2000;186 4:347–57. 10.1007/s003590050435 10798723

[20] Colbourne JK, Pfrender ME, Gilbert D, Thomas WK, Tucker A, Oakley TH, et al The ecoresponsive genome of Daphnia pulex. Science. 2011;331 6017:555–61. doi: 10.1126/science.1197761 21292972 10.1126/science.1197761 PMC3529199

[21] Havird JC and Santos SR. Here we are, but where do we go? A systematic review of crustacean transcriptomic studies from 2014–2015. Integr Comp Biol. 2016;56:1055–66. doi: 10.1093/icb/icw061 27400974 10.1093/icb/icw061 PMC6281346

[22] Francis WR, Christianson LM, Kiko R, Powers ML, Shaner NC and Haddock SH. A comparison across non-model animals suggests an optimal sequencing depth for de novo transcriptome assembly. BMC Genomics. 2013;14 1:167. 23496952 10.1186/1471-2164-14-167 PMC3655071

[23] Lenz PH, Roncalli V, Hassett RP, Wu LS, Cieslak MC, Hartline DK, et al De novo assembly of a transcriptome for Calanus finmarchicus (Crustacea, Copepoda)—the dominant zooplankter of the North Atlantic Ocean. PLoS ONE. 2014;9 2:e88589 doi: 10.1371/journal.pone.0088589 24586345 10.1371/journal.pone.0088589 PMC3929608

[24] Haas BJ, Papanicolaou A, Yassour M, Grabherr M, Blood PD, Bowden J, et al De novo transcript sequence reconstruction from RNA-seq using the Trinity platform for reference generation and analysis. Nat Protoc. 2013;8 8:1494–512. doi: 10.1038/nprot.2013.084 23845962 10.1038/nprot.2013.084 PMC3875132

[25] Langmead B, Trapnell C, Pop M and Salzberg SL. Ultrafast and memory-efficient alignment of short DNA sequences to the human genome. Genome Biol. 2009;10 3 doi: 10.1186/Gb-2009-10-3-R25 19261174 10.1186/gb-2009-10-3-r25 PMC2690996

[26] Altschul SF, Madden TL, Schaffer AA, Zhang JH, Zhang Z, Miller W, et al Gapped BLAST and PSI-BLAST: a new generation of protein database search programs. Nucleic Acids Res. 1997;25 17:3389–402. doi: 10.1093/Nar/25.17.3389 9254694 10.1093/nar/25.17.3389 PMC146917

[27] Bairoch A and Apweiler R.The SWISS-PROT protein sequence database and its supplement TrEMBL in 2000. Nucleic Acids Res. 2000;28 1, 45–48. 10592178 10.1093/nar/28.1.45 PMC102476

[28] The UniProt Consortium; UniProt: the universal protein knowledgebase. Nucleic Acids Res 2017; 45 (D1): D158–D169. doi: 10.1093/nar/gkw1099 27899622 10.1093/nar/gkw1099 PMC5210571

[29] Simão FA, Waterhouse RM, Ioannidis P, Kriventseva EV and Zdobnov EM. BUSCO: assessing genome assembly and annotation completeness with single-copy orthologs. Bioinformatics. 2015;31 19:3210–2. doi: 10.1093/bioinformatics/btv351 26059717 10.1093/bioinformatics/btv351

[30] Katoh K and Standley DM. MAFFT multiple sequence alignment software version 7: improvements in performance and usability. Mol Biol Evol. 2013;30 4:772–80. doi: 10.1093/molbev/mst010 23329690 10.1093/molbev/mst010 PMC3603318

[31] Catterall WA, Goldin AL and Waxman SG. International Union of Pharmacology. XXXIX. Compendium of voltage-gated ion channels: Sodium channels. Pharmacol Rev. 2003;55 4:575–8. doi: 10.1124/pr.55.4.7 14657413 10.1124/pr.55.4.7

[32] Finn RD, Coggill P, Eberhardt RY, Eddy SR, Mistry J, Mitchell AL, et al The Pfam protein families database: towards a more sustainable future. Nucleic Acids Res. 2016;44 D1:D279–D85. doi: 10.1093/nar/gkv1344 26673716 10.1093/nar/gkv1344 PMC4702930

[33] Christie AE, Fontanilla TM, Nesbit KT and Lenz PH. Prediction of the protein components of a putative Calanus finmarchicus (Crustacea, Copepoda) circadian signaling systems using a de novo assembled transcriptome. Comp Biochem Phys D. 2013;8:165–93. doi: 10.1016/j.cbd.2013.04.002 23727418 10.1016/j.cbd.2013.04.002 PMC3755088

[34] Nesbit KT and Christie AE. Identification of the molecular components of a Tigriopus californicus (Crustacea, Copepoda) circadian clock. Comp Biochem Phys D. 2014;12:16–44. doi: 10.1016/j.cbd.2014.09.002 25310881 10.1016/j.cbd.2014.09.002

[35] Tilden AR, McCoole MD, Harmon SM, Baer KN and Christie AE. Genomic identification of a putative circadian system in the cladoceran crustacean Daphnia pulex. Comp Biochem Phys D. 2011;6 3:282–309. doi: 10.1016/J.Cbd.2011.06.002 21798832 10.1016/j.cbd.2011.06.002 PMC3994191

[36] Gramates LS, Marygold SJ, dos Santos G, Urbano JM, Antonazzo G, Matthews BB, et al FlyBase at 25: looking to the future. Nucleic Acids Res. 2016;45 D1:D663–D71. doi: 10.1093/nar/gkw1016 27799470 10.1093/nar/gkw1016 PMC5210523

[37] Christie AE. Neuropeptide discovery in Eucyclops serrulatus (Crustacea, Copepoda): in silico prediction of the first peptidome for a member of the Cyclopoida. Gen Comp Endocrinol. 2015;211:92–105. doi: 10.1016/j.ygcen.2014.11.002 25448253 10.1016/j.ygcen.2014.11.002

[38] Christie AE, Roncalli V, Wu LS, Ganote CL, Doak T and Lenz PH. Peptidergic signaling in Calanus finmarchicus (Crustacea, Copepoda): In silico identification of putative peptide hormones and their receptors using a de novo assembled transcriptome. Gen Comp Endocrinol. 2013;187:117–35. doi: 10.1016/j.ygcen.2013.03.018 23578900 10.1016/j.ygcen.2013.03.018

[39] Petersen TN, Brunak S, Von Heijne G and Nielsen H. SignalP 4.0: discriminating signal peptides from transmembrane regions. Nature Methods. 2011;8 10:785–6. doi: 10.1038/nmeth.1701 21959131 10.1038/nmeth.1701

[40] Monigatti F, Gasteiger E, Bairoch A and Jung E. The Sulfinator: predicting tyrosine sulfation sites in protein sequences. Bioinformatics. 2002;18 5:769–70. doi: 10.1093/bioinformatics/18.5.769 12050077 10.1093/bioinformatics/18.5.769

[41] Schaeffer L, Pimentel H, Bray N, Melsted P, Pachter L. Pseudoalignment for metagenomic read assignment. 2015.arXiv preprint arXiv:1510.07371. 10.1093/bioinformatics/btx106 PMC5870846 28334086

[42] Robinson MD, McCarthy DJ and Smyth G.K. edgeR: a Bioconductor package for differential expression analysis of digital gene expression data. Bioinformatics, 2010;26 1:139–140. doi: 10.1093/bioinformatics/btp616 19910308 10.1093/bioinformatics/btp616 PMC2796818

[43] Oliveros JC. Venny. An interactive tool for comparing lists with Venn's diagrams. 2007–2015. http://bioinfogp.cnb.csic.es/tools/venny/index.html.

[44] Hulsen T, de Vlieg J, Alkema W. BioVenn–a web application for the comparison and visualization of biological lists using area-proportional Venn diagrams. BMC genomics. 2008;9 1:488. 18925949 10.1186/1471-2164-9-488 PMC2584113

[45] Tarrant AM, Baumgartner MF, Hansen BH, Altin D, Nordtug T and Olsen AJ. Transcriptional profiling of reproductive development, lipid storage and molting throughout the last juvenile stage of the marine copepod Calanus finmarchicus. Front Zool. 2014;11 1:1 doi: 10.1186/s12983-014-0091-8 25568661 10.1186/s12983-014-0091-8 PMC4285635

[46] Yang Q, Sun F, Yang Z and Li H. Comprehensive transcriptome study to develop molecular resources of the copepod Calanus sinicus for their potential ecological applications. BioMed Res Int. 2014;2014 doi: 10.1155/2014/493825 24982883 10.1155/2014/493825 PMC4055022

[47] Ning J, Wang MX, Li CL and Sun S. Transcriptome sequencing and de novo analysis of the copepod Calanus sinicus using 454 GS FLX. PLoS ONE. 2013;8 5:e63741 doi: 10.1371/journal.pone.0063741 23671698 10.1371/journal.pone.0063741 PMC3646036

[48] Almada AA, Tarrant AM. Vibrio elicits targeted transcriptional responses from copepod hosts. FEMS Microb Ecol. 2016; 1 92 6. 10.1093/femsec/fiw072 27056917

[49] Tassone EE, Geib SM, Hall B, Fabrick JA, Brent CS, Hull JJ. De novo construction of an expanded transcriptome assembly for the western tarnished plant bug, Lygus hesperus. GigaScience. 2016;28 5:1:6. 10.1186/s13742-016-0109-6 PMC4730634 26823975

[50] Tassone EE, Cowden CC, Castle SJ. De novo transcriptome assemblies of four xylem sap-feeding insects. GigaScience. 2017;1 6 3:1–4. 10.1093/gigascience/giw007 PMC5467018 28327966

[51] Kim HS, Lee BY, Han J, Lee YH, Min GS, Kim S, et al De novo assembly and annotation of the Antarctic copepod (Tigriopus kingsejongensis) transcriptome. Marine genomics. 2016;28:37–9. doi: 10.1016/j.margen.2016.04.009 27157881 10.1016/j.margen.2016.04.009

[52] Lee BY, Kim HS, Choi BS, Hwang DS, Choi AY, Han J. et al RNA-seq based whole transcriptome analysis of the cyclopoid copepod Paracyclopina nana focusing on xenobiotics metabolism. Comp Biochem Physiol D 2015; 15: 12–19. 10.1016/j.cbd.2015.04.002 26001055

[53] Kim HS, Lee BY, Won EJ, Han J, Hwang DS, Park HG, et al Identification of xenobiotic biodegradation and metabolism-related genes in the copepod Tigriopus japonicus whole transcriptome analysis. Marine genomics. 2015;24:207–8. doi: 10.1016/j.margen.2015.05.011 26024611 10.1016/j.margen.2015.05.011

[54] Milligan MJ, Lipovich L. Pseudogene-derived lncRNAs: emerging regulators of gene expression. Front Genet. 2014;5. 10.3389/fgene.2014.00476 PMC4316772 25699073

[55] Brown JB, Boley N, Eisman R, May GE, Stoiber MH, Duff MO, et al Diversity and dynamics of the Drosophila transcriptome. Nature. 2014. 10.1038/nature12962 PMC4152413 24670639

[56] Alberts B, Johnson A, Lewis J, Morgan D, Raff M, Roberts K, et al Molecular Biology of the Cell. 6th ed. New York, NY: Garland Science; 2014.

[57] Slingsby C, Wistow GJ and Clark AR. Evolution of crystallins for a role in the vertebrate eye lens. Protein Science.2013; 22 4:367–380. doi: 10.1002/pro.2229 23389822 10.1002/pro.2229 PMC3610043

[58] Stahl AL, Charlton-Perkins M, Buschbeck EK and Cook TA. The cuticular nature of corneal lenses in Drosophila melanogaster. 2017 Dev Genes Evol. 1–8. 28477155 10.1007/s00427-017-0582-7 PMC5581546

[59] Cohen JH, Piatigorsky J, Ding L, Colley NJ, Ward R and Horwitz J. Vertebrate-like βγ-crystallins in the ocular lenses of a copepod. J Comp Physiol A. 2005;19:291–8. 10.1007/s00359-004-0594-4 15702356

[60] Cohen JH, Piatigorsky J, Ding L, Colley NJ, Ward R, Horwitz J. ERRATUM: Vertebrate-like βγ-crystallins in the ocular lenses of a copepod. J Comp Physiol A. 2007;1;193 5:573–4. 10.1007/s00359-004-0594-4 15702356

[61] Hunt ME, Scherrer MP, Ferrari FD, Matz MV. Very bright green fluorescent proteins from the Pontellid copepod Pontella mimocerami. PLoS ONE. 2010;5 7:e11517 doi: 10.1371/journal.pone.0011517 20644720 10.1371/journal.pone.0011517 PMC2904364

[62] Roncalli V, Cieslak MC and Lenz PH. Transcriptomic responses of the calanoid copepod Calanus finmarchicus to the saxitoxin producing dinoflagellate Alexandrium fundyense. Sci Rep. 2016;6:25708 doi: 10.1038/srep25708 27181871 10.1038/srep25708 PMC4867593

[63] Porter M, Steck M, Roncalli V, Lenz PH. Molecular characterization of copepod photoreception. Biol Bull.2017 In press. 10.1086/694564 29182504

[64] Goldin AL. Evolution of voltage-gated Na+ channels. J Exp Biol. 2002;205 5:575–84. 11907047 10.1242/jeb.205.5.575

[65] Allada R and Chung BY. Circadian organization of behavior and physiology in Drosophila. Annu Rev Physiol. 2010;72:605–24. doi: 10.1146/annurev-physiol-021909-135815 20148690 10.1146/annurev-physiol-021909-135815 PMC2887282

[66] Hardin PE. Molecular genetic analysis of circadian timekeeping in Drosophila. Adv Genet. 2011;74:141–73. doi: 10.1016/B978-0-12-387690-4.00005-2 21924977 10.1016/B978-0-12-387690-4.00005-2 PMC4108082

[67] Reppert SM. The ancestral circadian clock of monarch butterflies: role in time-compensated sun compass orientation. In:Cold Spring Harbor Symposia on quantitative biology. 2007;72:113–6. doi: 10.1101/sqb.2007.72.056 18419268 10.1101/sqb.2007.72.056

[68] Yuan Q, Metterville D, Briscoe AD and Reppert SM. Insect cryptochromes: gene duplication and loss define diverse ways to construct insect circadian clocks. Mol Biol Evol. 2007;24 4:948–55. doi: 10.1093/molbev/msm011 17244599 10.1093/molbev/msm011

[69] Sbragaglia V, Lamanna F, Mat AM, Rotllant G, Joly S, Ketmaier V, et al Identification, characterization, and diel pattern of expression of canonical clock genes in Nephrops norvegicus (Crustacea: Decapoda) eyestalk. PLoS ONE. 2015;10:e0141893 doi: 10.1371/journal.pone.0141893 26524198 10.1371/journal.pone.0141893 PMC4629887

