# Supplementary figures and images for: A deep transcriptomic resource for the copepod crustacean Labidocera madurae: A potential indicator species for assessing near shore ecosystem health
Source: PLoS One. 2017 Oct 24;12(10):e0186794. doi: 10.1371/journal.pone.0186794 (PMC5655441; doi:10.1371/journal.pone.0186794)

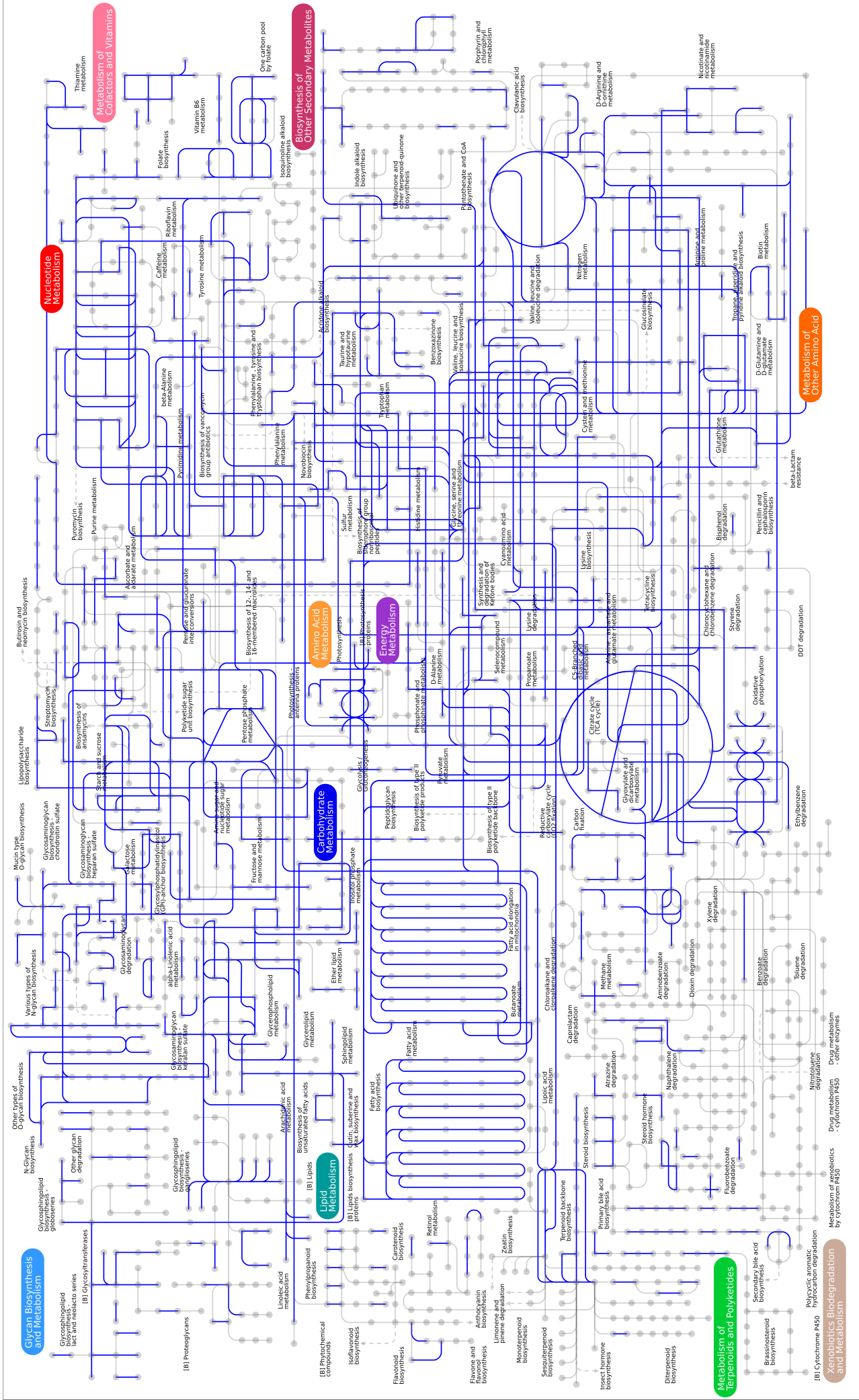

Supplement: S1 Fig — Diagram in light purple is a map of 146 KEGG pathways that provide a generalized overview of global metabolism in eukaryotes. Metabolic compounds are identified by nodes, while the lines show enzymatic transformations. Highlighted blue lines and corresponding nodes represent the pathways that were annotated in the L. madurae transcriptome using SwissProt and KEGG pathway analysis. The KEGG map was customized using ipath2. (PDF) [file pone.0186794.s001.pdf]

## Pred.genes-CDS

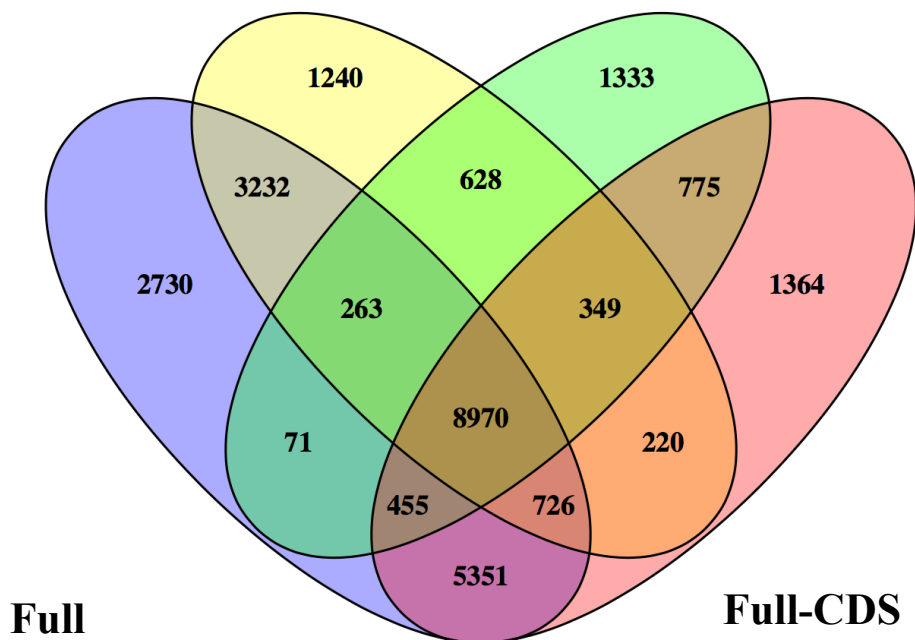

B

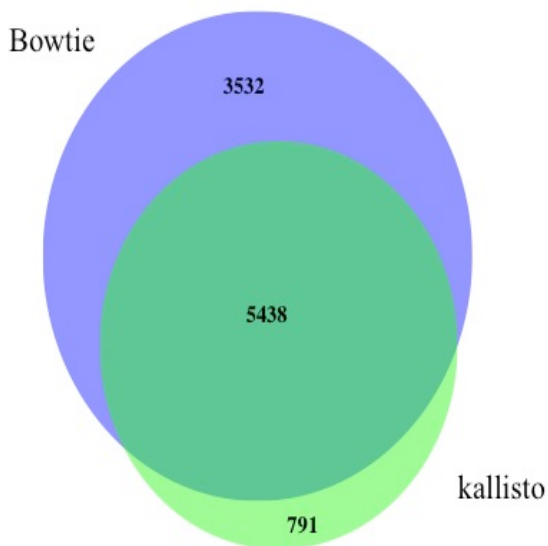

C

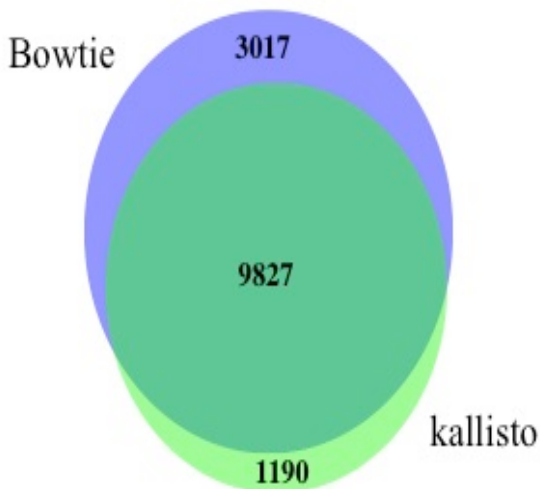

Supplement: S4 Fig — The reference transcriptomes are defined as: “Full” with 211K transcripts, “Pred. genes” consisting of longest transcript for Trinity predicted genes, “Pred.genes-CDS” consisting of transcripts with coding regions (CDS) from the “Pred.genes” and “Full-CDS” consisting of transcripts with coding regions (CDS) from “Full”. A) Non-proportional Venn diagram comparing all four transcriptomes for the number of identified DEGs using Bowtie as the mapping program. “Full” transcriptome (purple), “Pred. genes” (yellow), “Pred.genes-CDS”(green) and “Full-CDS” (pink). B) Proportional Venn diagram comparing the DEGs that were shared among all four reference transcriptomes using either Bowtie (purple) or kallisto (green) as the mapping program. C) Proportional Venn diagram comparing DEGs identified using the smallest reference “Pred. genes-CDS” using either Bowtie (purple) or kallisto (green) as the mapping program. DEGs were separately identified for each transcriptome and mapping combination using edgeR set to P-value <0.05 and false discovery rate (FDR) cutoff of 5%. (PDF) [file pone.0186794.s004.pdf]
