# Supplementary material for: A deep transcriptomic resource for the copepod crustacean Labidocera madurae: A potential indicator species for assessing near shore ecosystem health
Source: PLoS One. 2017 Oct 24;12(10):e0186794. doi: 10.1371/journal.pone.0186794 (PMC5655441; doi:10.1371/journal.pone.0186794)
Supplement: S2 Fig — Variants were aligned using MAFFT. In the line immediately below each sequence grouping, “*” indicates identical amino acid residues, while “:” and “.” denote amino acids that are similar in structure between sequences. In this figure, helix-loop-helix DNA-binding, PAS fold, and PAS domains identified by Pfam analyses are highlighted in yellow, light green, and light blue, respectively. (DOCX) [file pone.0186794.s002.docx]

S2 Fig.

Labma-CYC-v1 MNQAGESQPDYTASHDGFHYQNLVFPDFLHEGPGILNMMEFQGYSEDLATSPGHSEKKRK

Labma-CYC-v2a MNQAGESQPDYTASHDGFHYQNLVFPDFLHEGPGILNMMEFQGYSEDLATSPGHSEKKRK

Labma-CYC-v2b MNQAGESQPDYTASHDGFHYQNLVFPDFLHEGPGILNMMEFQGYSEDLATSPGHSEKKRK

Labma-CYC-v3 -------------------------------------MMEFQGYSEDLATSPGHSEKKRK

Labma-CYC-v4 -------------------------------------MMEFQGYSEDLATSPGHSEKKRK

***********************

Labma-CYC-v1 LPPEEDTHLPEETKQRKILSQDVSPLKEEQKRYVRQNHSEIEKRRRDKMNTYITELSSMI

Labma-CYC-v2a LPPEEDTHLPEETKQRKILSQ------EEQKRYVRQNHSEIEKRRRDKMNTYITELSSMI

Labma-CYC-v2b LPPEEDTHLPEETKQRKILSQ------EEQKRYVRQNHSEIEKRRRDKMNTYITELSSMI

Labma-CYC-v3 LPPEEDTHLPEETKQRKILSQDVSPLKEEQKRYVRQNHSEIEKRRRDKMNTYITELSSMI

Labma-CYC-v4 LPPEEDTHLPEETKQRKILSQ------EEQKRYVRQNHSEIEKRRRDKMNTYITELSSMI

********************* *********************************

Labma-CYC-v1 PTCVAMQRKMDKLTVLRLAVQHLKSIRGSLDAYSEGNSRPAMLTDSELRQLIVPSADGFI

Labma-CYC-v2a PTCVAMQRKMDKLTVLRLAVQHLKSIRGSLDAYSEGNSRPAMLTDSELRQLIVPSADGFI

Labma-CYC-v2b PTCVAMQRKMDKLTVLRLAVQHLKSIRGSLDAYSEGNSRPAMLTDSELRQLIVPSADGFI

Labma-CYC-v3 PTCVAMQRKMDKLTVLRLAVQHLKSIRGSLDAYSEGNSRPAMLTDSELRQLIVPSADGFI

Labma-CYC-v4 PTCVAMQRKMDKLTVLRLAVQHLKSIRGSLDAYSEGNSRPAMLTDSELRQLIVPSADGFI

************************************************************

Labma-CYC-v1 FVVDSARTRILYVSESVNNILNFSSQDLIGQSLFDILHPKDIDKVKEQLSNSDGPRSRLI

Labma-CYC-v2a FVVDSARTRILYVSESVNNILNFSSQDLIGQSLFDILHPKDIDKVKEQLSNSDGPRSRLI

Labma-CYC-v2b FVVDSARTRILYVSESVNNILNFSSQDLIGQSLFDILHPKDIDKVKEQLSNSDGPRSRLI

Labma-CYC-v3 FVVDSARTRILYVSESVNNILNFSSQDLIGQSLFDILHPKDIDKVKEQLSNSDGPRSRLI

Labma-CYC-v4 FVVDSARTRILYVSESVNNILNFSSQDLIGQSLFDILHPKDIDKVKEQLSNSDGPRSRLI

************************************************************

Labma-CYC-v1 DSKTMLPLKVGEIPQSIGRLQPGARRVFFCRMKCKPTQMIKQEDDIYPILNSNSVQPQEN

Labma-CYC-v2a DSKTMLPLKVGEIPQSIGRLQPGARRVFFCRMKCKPTQMIKQEDDIYPILNSNSVQPQEN

Labma-CYC-v2b DSKTMLPLKVGEIPQSIGRLQPGARRVFFCRMKCKPTQMIKQEDDIYPILNSNSVQPQEN

Labma-CYC-v3 DSKTMLPLKVGEIPQSIGRLQPGARRVFFCRMKCKPTQMIKQEDDIYPILNSNSVQPQEN

Labma-CYC-v4 DSKTMLPLKVGEIPQSIGRLQPGARRVFFCRMKCKPTQMIKQEDDIYPILNSNSVQPQEN

************************************************************

Labma-CYC-v1 NGKKKKCGNSDKKYISIQCTGYLKSWPFTKVGLEGEFPDLDADSDTCMSCLVAVGRVQPS

Labma-CYC-v2a NGKKKKCGNSDKKYISIQCTGYLKSWPFTKVGLEGEFPDLDADSDTCMSCLVAVGRVQPS

Labma-CYC-v2b NGKKKKCGNSDKKYISIQCTGYLKSWPFTKVGLEGEFPDLDADSDTCMSCLVAVGRVQPS

Labma-CYC-v3 NGKKKKCGNSDKKYISIQCTGYLKSWPFTKVGLEGEFPDLDADSDTCMSCLVAVGRVQPS

Labma-CYC-v4 NGKKKKCGNSDKKYISIQCTGYLKSWPFTKVGLEGEFPDLDADSDTCMSCLVAVGRVQPS

************************************************************

Labma-CYC-v1 FQSTIEDCIERGETTSTAVEFFSRHGIDGKFSFVDQRVTLMLGYLPQELVGTSLYEHIQY

Labma-CYC-v2a FQSTIEDCIERGETTSTAVEFFSRHGIDGKFSFVDQRVTLMLGYLPQELVGTSLYEHIQY

Labma-CYC-v2b FQSTIEDCIERGETTSTAVEFFSRHGIDGKFSFVDQRVTLMLGYLPQELVGTSLYEHIQY

Labma-CYC-v3 FQSTIEDCIERGETTSTAVEFFSRHGIDGKFSFVDQRVTLMLGYLPQELVGTSLYEHIQY

Labma-CYC-v4 FQSTIEDCIERGETTSTAVEFFSRHGIDGKFSFVDQRVTLMLGYLPQELVGTSLYEHIQY

************************************************************

Labma-CYC-v1 DDIPLIAECHRKSLRNSDEVNTPIFGFRTKDGNFVKLKSKFKHFRNPWTREIDYIFCKNY

Labma-CYC-v2a DDIPLIAECHRKSLRNSDEVNTPIFGFRTKDGNFVKLKSKFKHFRNPWTREIDYIFCKNY

Labma-CYC-v2b DDIPLIAECHRKSLRNSDEVNTPIFGFRTKDGNFVKLKSKFKHFRNPWTREIDYIFCKNY

Labma-CYC-v3 DDIPLIAECHRKSLRNSDEVNTPIFGFRTKDGNFVKLKSKFKHFRNPWTREIDYIFCKNY

Labma-CYC-v4 DDIPLIAECHRKSLRNSDEVNTPIFGFRTKDGNFVKLKSKFKHFRNPWTREIDYIFCKNY

************************************************************

Labma-CYC-v1 LIFSKEKYSESSNFGSADTADMDFIMANNSGRHNGGTQTGAGNGGNSGVGANNGQIGKDI

Labma-CYC-v2a LIFSKEKYSESSNFGSADTADMDFIMANNSGRHNGGTQTGAGNGGNSGVGANNGQIGKDI

Labma-CYC-v2b LIFSKEKYSESSNFGSADTADMDFIMANNSGRPNGGTQTGAGHGGNSGGGANNGQIGKDI

Labma-CYC-v3 LIFSKEKYSESSNFGSADTADMDFIMANNSGRHNGGTQTGAGNGGNSGVGANNGQIGKDI

Labma-CYC-v4 LIFSKEKYSESSNFGSADTADMDFIMANNSGRHNGGTQTGAGNGGNSGVGANNGQIGKDI

******************************** *********:***** ***********

Labma-CYC-v1 QQVISSHAEAAKIGRNIADEEIEKWRTDNSASNSPISSLQSGSPSTLQSSTSINPASSAL

Labma-CYC-v2a QQVISSHAEAAKIGRNIADEEIEKWRTDNSASNSPISSLQSGSPSTLQSSTSINPASSAL

Labma-CYC-v2b QQVISSHAEAAKIGRNIADEEIEKWRTDNSASNSPISSLQSGSPSTLQSSTSINPASSAL

Labma-CYC-v3 QQVISSHAEAAKIGRNIADEEIEKWRTDNSASNSPISSLQSGSPSTLQSSTSINPASSAL

Labma-CYC-v4 QQVISSHAEAAKIGRNIADEEIEKWRTDNSASNSPISSLQSGSPSTLQSSTSINPASSAL

************************************************************

Labma-CYC-v1 GGKGEAELIREAMLDVERDRNKLSGVIVSRNSSTSNATLNRLVTNRARIDQIPAAALSAR

Labma-CYC-v2a GGKGEAELIREAMLDVERDRNKLSGVIVSRNSSTSNATLNRLVTNRARIDQIPAAALSAR

Labma-CYC-v2b GGKGEAELIREAMLDVERDRNKLSGVIVSRNSSTSNATLNRLVTNRARIDQIPAAALSAR

Labma-CYC-v3 GGKGEAELIREAMLDVERDRNKLSGVIVSRNSSTSNATLNRLVTNRARIDQIPAAALSAR

Labma-CYC-v4 GGKGEAELIREAMLDVERDRNKLSGVIVSRNSSTSNATLNRLVTNRARIDQIPAAALSAR

************************************************************

Labma-CYC-v1 TSPPHSTSSSESGNDEAATAVLMSLLEAEGGLGGPFDFGSLPWPLP

Labma-CYC-v2a TSPPHSTSSSESGNDEAATAVLMSLLEAEGGLGGPFDFGSLPWPLP

Labma-CYC-v2b TSPPHSTSSSESGNDEAATAVLMSLLEAEGGLGGPFDFGSLPWPLP

Labma-CYC-v3 TSPPHSTSSSESGNDEAATAVLMSLLEAEGGLGGPFDFGSLPWPLP

Labma-CYC-v4 TSPPHSTSSSESGNDEAATAVLMSLLEAEGGLGGPFDFGSLPWPLP

**********************************************
