# Supplementary material for: A deep transcriptomic resource for the copepod crustacean Labidocera madurae: A potential indicator species for assessing near shore ecosystem health
Source: PLoS One. 2017 Oct 24;12(10):e0186794. doi: 10.1371/journal.pone.0186794 (PMC5655441; doi:10.1371/journal.pone.0186794)
Supplement: S3 Fig — Variants were aligned using MAFFT. In the line immediately below each sequence grouping, “*” indicates identical amino acid residues, while “:” and “.” denote amino acids that are similar in structure between sequences. In this figure, serine-threonine protein phosphatase N-terminal and calcineurin-like phosphoesterase domains identified by Pfam analyses are highlighted in blue and red, respectively. (PDF) [file pone.0186794.s003.pdf]

Figure S3

```
Labma-PP1-I      M-----------------------------------------------------------
Labma-PP1-II     MDAK----------FGGP----------------------------------------GG
Labma-PP1-III    M----------------------------------------------------------G
Labma-PP1-IV     MSIEVPTSDSSAINYSTPIQDVNNVSRRPTIKNPVKPLTRMMRNLKDANGSSVPNLTTNG
                 *

Labma-PP1-I      ---AEGEINIDNIIQRLLEVRGS-RPGKTVQMSEAEVRGLCLKSRELFLQQPILLELEAP
Labma-PP1-II     GGKETDKLNIDSIIARLLEVRGS-RPGKNVQLSEYEIRGLCLKSRELFLSQPILLELEAP
Labma-PP1-III    EKGDVSEVDVDSIIDRLLEVRGC-RPGKQVQLDEHEIKWLCSKSREIFISQPILLELEAP
Labma-PP1-IV     TSNTQSNQLIDSIISKLIDIGMSGRVPRVLPIKSSDILWLLAQVQPILLSQPTLLEIQAP
                      .:  :*.** :*:::  . *  : : :.. ::  *  : : :::.** ***::**

Labma-PP1-I      LKICGDIHGQYTDLLRLFEYGGFPPEANYLFLGDYVDRGKQSLETICLLLAYKIKYPENF
Labma-PP1-II     LKICGDVHGQYYDLLRLFEYGGFPPESNYLFLGDYVDRGKQSLETVCLLLAYKIKYPENF
Labma-PP1-III    IKICGDIHGQYYDLLRLFEYGGFPPEANYLFLGDYVDRGKQSLETICLLLAYKIKYPENF
Labma-PP1-IV     VKIVGDIHGQYLDLMRIFTKSGWPPGSNYLFLGDYVDRGKQSLETILLLFAFKVRYKGRF
                 :** **:**** **:*:*  .*:** :******************: **:*:*::*  .*

Labma-PP1-I      FLLRGNHECASINRIYGFYDECKRRYNIKLWKTFTDCFNCLPIAAIVDEKIFCCHGGLSP
Labma-PP1-II     FLLRGNHECASINRIYGFYDECKRRYNVKLWKTFTDCFNCLPVAAIVDEKIFCCHGGLSP
Labma-PP1-III    FILRGNHECASINRIYGFYDECKRRYNIKLWKTFTDCFNCLPIAAIIDEKIFTMHGGLSP
Labma-PP1-IV     FLLRGNHECQGICRVYGFYDECKRRASVKIWKSFVDTFNCLPIAAVVADKIFCVHGGLSP
                 *:******* .* *:********** .:*:**:*.* *****:**:: :***  ******

Labma-PP1-I      DLQSMEQIRRIMRPTDVPDTGLLCDLLWSDPDKDVQGWGENDRGVSFTFGADVVSKFLNR
Labma-PP1-II     DLQSMEQIRRIMRPTDVPDQGLLCDLLWSDPDKDTMGWGENDRGVSFTFGAEVVSKFLHK
Labma-PP1-III    DLQSMEQIRRVMRPTDVPDTGLLCDLLWSDPEKEITGWGENDRGVSFTFGPDVVSRFLQN
Labma-PP1-IV     ELNSMQRIRDIERPTEVPDFGLINDLLWSDPSHSAVDWEDSDRGVSYCFGRDILSRFLHK
                 :*:**::** : ***:*** **: *******.:.  .* :.*****: ** :::*:**:.

Labma-PP1-I      HDLDLICRAHQVVEDGYEFFAKRQLVTLFSAPNYCGEFDNAGGMMSVDETLMCSFQILKP
Labma-PP1-II     HDFDLICRAHQVVEDGYEFFAKRQLVTLFSAPNYCGEFDNAGAMMSVDETLMCSFQILKP
Labma-PP1-III    HEMDLICRAHQVVEDGYEFFAKRQLVTLFSAPNYCGEFDNAGAMMSVDDTLMCSFQILKP
Labma-PP1-IV     HKFDLLARAHMVVEDGYEFFANRGLVTIFSAPNYCGIFKNMGAVMIVEPDLVCRFDLLQP
                 *.:**:.*** **********:* ***:******** *.* *.:* *:  *:* *::*:*

Labma-PP1-I      SEKKAKYQYQGLN--RPQT-PRGPQNNPGNIK----------------------------
Labma-PP1-II     ADKK-KFPYGGLNSSRPLTPPRGATQQKGKKK----------------------------
Labma-PP1-III    AEKKQKYTYAA-----------------GRMG----------------------------
Labma-PP1-IV     ASQA-SVTLQDEVVKADMAAAHAHAQLQNKDGNGTTGDGSNVNAQDRASPGGSVLSRSLS
                 :.:  .                      ..

Labma-PP1-I      -----------------------------------------------RK
Labma-PP1-II     -------------------------------------------------
Labma-PP1-III    -------------------------------------------------
Labma-PP1-IV     TKGGKVLKSPGLGHKSVNGDQGVISGARWNPNESPPEGSLLITRDKSKR
```
