# Supplementary material for: A deep transcriptomic resource for the copepod crustacean Labidocera madurae: A potential indicator species for assessing near shore ecosystem health
Source: PLoS One. 2017 Oct 24;12(10):e0186794. doi: 10.1371/journal.pone.0186794 (PMC5655441; doi:10.1371/journal.pone.0186794)
Supplement: S5 Fig — (DOC) [file pone.0186794.s005.doc]

**Supplementary File 11:** *Irregularities in the* L. madurae *assembly*

1) *How many Na_V_ genes?* The *L. madurae* transcriptome contains Na_V_ isoforms from 3 genes, including Na_V_2 reconstructed from two TR# fragments. This compares with at least 4 genes for *C. finmarchicus* [23]. Evidence also turned up for a single additional gene in each species. As *L. madurae* belongs to a more basal taxon than *C. finmarchicus*, one fewer Na_V_ genes may represent an evolutionary difference, and so we do not consider that it reflects on the quality of the transcriptome. A 486-residue contig assigned to a different gene family (TR25803|c0_g1_i1; Table 4) appears to represent an additional splice variant of Labma Na_V_1.1, one with its coding region having high identity (72%) to a similar fragment from *Eurytemora affinis* (accession [GBGO01072110](https://www.ncbi.nlm.nih.gov/Traces/wgs/GBGO01/GBGO01072110?rettype=fasta)).

2) *Shared common sequences with variable flanks.* Several cases arise in the *L. madurae* transcriptome of isoforms that have two or more variable regions separated by a shared common portion of sequence. As described in the main text, this can be problematic if the divergent flanking portions are separated by more than one cDNA-insert-length. The example given was for a potential error in assigning splice variants separated by 630 amino acid residues. The Calfi Na_V_1.1 gene, like that of *L. madurae*, also has two regions of splice variation separated by a long stretch of identical sequence [23]. Trinity 1.0 constructed 17 isoforms using various combinations of variants from the two regions, which is equally unreliable. While this gives Calfi Na_V_1.1 the outward appearance of being better assembled as full-length isoforms than Labma Na_V_1.1, such a conclusion may not be warranted. In the latter case with the Trinity 2.0.6 assembly, this misleading multiplicity of combinations did not occur as extensively.

As another example, all of the Labma CRY2 assembled isoforms have in common the identical 2478-long nucleic acid sequence that includes most of the coding region. As assembled, however, there are 5 distinct 5' UTRs and 11 distinct 3' UTRs, as shown in the diagram below. This should provide 55 (5×11) possible UTR combinations, none of which is certain because of the long identical coding sequence held in common. However, 12 combinations are represented in the assembly. Without additional information, it is unclear which combinations of the variants of the two flanking regions are valid.

| Labma CRY2 | 5' UTR | | | | | | 3' UTR | | | | | | | | | | | | | | | | | |
| --- | --- | --- | --- | --- | --- | --- | --- | --- | --- | --- | --- | --- | --- | --- | --- | --- | --- | --- | --- | --- | --- | --- | --- | --- |
| Sequence | 1 | 2 | 3 | 4 | 5 | 6 | 1 | 2 | 3 | 4 | 5 | 6 | 7 | 8 | 9 | 10 | 11 | 12 | 13 | 14 | 15 | 16 | 17 | 18 |
| TR24805c1g1_i12 |  |  |  |  |  |  |  |  |  |  |  |  |  |  |  |  |  |  |  |  |  |  |  |  |
| TR24805c1g1_i6 |  |  |  |  |  |  |  |  |  |  |  |  |  |  |  |  |  |  |  |  |  |  |  |  |
| TR24805c1g1_i7 |  |  |  |  |  |  |  |  |  |  |  |  |  |  |  |  |  |  |  |  |  |  |  |  |
| TR24805c1g1_i8 |  |  |  |  |  |  |  |  |  |  |  |  |  |  |  |  |  |  |  |  |  |  |  |  |
| TR24805c1g1_i9 |  |  |  |  |  |  |  |  |  |  |  |  |  |  |  |  |  |  |  |  |  |  |  |  |
| TR24805c1g1_i10 |  |  |  |  |  |  |  |  |  |  |  |  |  |  |  |  |  |  |  |  |  |  |  |  |
| TR24805c1g1_i3 |  |  |  |  |  |  |  |  |  |  |  |  |  |  |  |  |  |  |  |  |  |  |  |  |
| [TR24805c1g1_i4](#i4_and_i5) |  |  |  |  |  |  |  |  |  |  |  |  |  |  |  |  |  |  |  |  |  |  |  |  |
| TR24805c1g1_i2 |  |  |  |  |  |  |  |  |  |  |  |  |  |  |  |  |  |  |  |  |  |  |  |  |
| TR24805c1g1_i1 |  |  |  |  |  |  |  |  |  |  |  |  |  |  |  |  |  |  |  |  |  |  |  |  |
| TR24805c1g1_i11 |  |  |  |  |  |  |  |  |  |  |  |  |  |  |  |  |  |  |  |  |  |  |  |  |
| [TR24805c1g1_i5](#i4_and_i5) |  |  |  |  |  |  |  |  |  |  |  |  |  |  |  |  |  |  |  |  |  |  |  |  |

UTR segments (numbered columns) defined by sequences shared among two or more isoforms. In each segment, shared nt sequences are coded with the same color

3) *Partial proteins.* Transcript isoforms coding for partial proteins, e.g. especially within the Na_V_1.1 gene (Table 4), might seem at first glance to indicate poor assembly quality, as might arise from an inadequate assembly depth. However, this too can result from shared common sequences with variable flanks as just described. Rather than assembling full-length isoforms with all possible combinations of the variable flanks, it is not incorrect to terminate the sequence if the assembler cannot reliably match the two variable ends of a segment. The truncated sequence is still useful, reflecting presence of isoforms that contain it. This interpretation of a truncated sequence should be kept in mind in assessing transcriptome quality. However, breaking a sequence up into truncated fragments to avoid generating questionable full-length isoforms lowers N50s. Rather than reflecting on the quality of the assembly, it can be seen as resulting from the way the particular assembly program handles flanking variation.

An example is provided by our assembly of Labma Na_V_2 (Fig. 4): two N-terminal fragments (in TR65477) differed from each other by the presence or absence of a 55nt optional piece (arrow in Fig. 4). Similarly a 15nt optional piece marked the difference between the two C-terminal isoform fragments (in TR68660). Without further information one cannot determine which of the two forms of the N-terminal piece go with which of those of the C-terminal piece. However the N-terminal and C-terminal pieces overlap by 132 identical nucleotides (44 amino acids), confirming their identity as Na_V_2 fragments and showing how the fragments fit together in a reconstructed channel (Fig. 4). A good *de novo* assembly of a transcript with two or more well-separated (> cDNA-insertion-length) sites of variation, as in this case, cannot assemble it as a full-length sequence. It can nonetheless have a full-length protein (as translated), including its UTRs, laid out in overlapping pieces, with no missing sections. The ambiguity arising from variant regions does not interfere with this.

4) *Genetic variability* can provide another potential source of fragmentation in a transcriptome. The more individuals included in the original RNA sample, the more chance for such problems. This can lead to transcripts assembling as separate contigs because of differences in the nucleotide sequence, even though they may code for the same protein. An example comes from a Trinity 2.0.6 reassembly of one of the optional exons in the Na_V_1.1 of the *C. finmarchicus* transcriptome. This reassembly produced two contigs assigned to different Trinity predicted isoforms (TR93632|c0_g1_i1 and i2), both translating into the same amino acid sequence. The two contigs (see below) differ at a total of 16 nucleotide locations (2.4%), which presumably explains the divergent assignment (in contrast, the same region was assembled as part of full-length predicted proteins by Trinity 1.0 [23]). Fragmentation of this sort contributes to shorter mean contig lengths in the assembly statistics. A second feature of note in this example is the clustering of the sequence discrepancies at the two ends of the contigs. Each cluster is compact: the length of identical nucleotides spanning adjacent substitution sites never exceeds 22, which is well within the span covered by a 90 base-pair cDNA-insert (200-300 nt mean expected). Thus, the substitutions within a cluster are reliably grouped together. The sequences terminate at either end within one cDNA-insertion-length of the outermost variant nucleotide, which is conservative. However, the span of over 400 identical nucleotides between the two clusters exceeds one cDNA-insertion-length, so any pairing across this distance is problematic, in the absence of other information.

G P L E G N N S R R E S N H T N S S H G

GGGTCCTCTCGAGGGTAACAACAGCAGGAGGGAGAGCAACCATACAAACTCCTCCCATGG

GGGTCCTCTCGAGGGTAACAACAGCAGGAGGGAGAGCAATCATACAAATTCCTCACACGG

***************************************.********.***** **.**

P R K A S L S L P G S P F H R R L S R A

GCCTAGAAAGGCCAGTCTTAGTCTTCCTGGGTCTCCATTTCATAGACGGTTATCCCGAGC

TCCAAGAAAGGCCAGTCTAAGTCTTCCTGGGTCTCCATTTCATAGACGGTTATCCCGAGC

** ************** *****************************************

S Q A G S H T Y S W R S N G Y R F G E R

ATCTCAAGCCGGAAGTCATACATACTCATGGAGGTCAAATGGATATCGTTTTGGAGAGCG

ATCTCAAGCCGGAAGTCATACATACTCATGGAGGTCAAATGGATATCGTTTTGGAGAGCG

************************************************************

K Q W L R S S Y L D T R D H L P Y M D D

AAAGCAATGGCTGAGATCCTCCTACCTCGATACTCGAGACCATCTGCCTTATATGGACGA

AAAGCAATGGCTGAGATCCTCCTACCTCGATACTCGAGACCATCTGCCTTATATGGACGA

************************************************************

S R V G S P N G S L D G G V I Q L G Q G

TTCTCGAGTAGGGTCTCCAAATGGTAGCCTTGACGGTGGTGTGATTCAGCTTGGTCAAGG

TTCTCGAGTAGGGTCTCCAAATGGTAGCCTTGACGGTGGTGTGATTCAGCTTGGTCAAGG

************************************************************

W P L G P N S R H N S Y S S H T S R V T

ATGGCCACTTGGTCCAAACTCAAGACATAACTCATACTCTTCTCACACCTCAAGGGTTAC

ATGGCCACTTGGTCCAAACTCAAGACATAACTCATACTCTTCTCACACCTCAAGGGTTAC

************************************************************

Y N S H A E L T K G G G S R E L H W R R

TTATAATTCGCATGCTGAGCTCACCAAGGGTGGAGGTTCTCGCGAACTTCATTGGCGACG

TTATAATTCGCATGCTGAGCTCACCAAGGGTGGAGGTTCTCGCGAACTTCATTGGCGACG

************************************************************

S G P E R N T Q A P G P M G W G Q W V D

GTCTGGACCTGAACGAAATACTCAGGCCCCTGGGCCCATGGGTTGGGGACAGTGGGTGGA

GTCTGGACCTGAACGAAATACTCAGGCCCCTGGGCCCATGGGTTGGGGACAGTGGGTGGA

************************************************************

S F Y T T H D L P L P P N G H G G T V I

TAGCTTTTACACTACACATGATCTGCCCCTACCACCTAATGGACATGGGGGCACTGTGAT

TAGCTTTTACACTACTCATGATCTGCCCCTACCACCCAATGGACATGGGGGCACCGTAAT

*************** ********************.*****************.**.**

S M R D V V K T N L A D T V L P T K Q R

CAGCATGAGGGACGTCGTCAAGACCAACCTGGCGGACACCGTCCTGCCTACCAAGCAACG

CAGCATGAGAGACGTTGTCAAGACCAACCTGGCAGACACTGTCCTGCCTACCAAGCAACG

*********.*****.*****************.*****.********************

L S V Q F P A N F P H Y F S P G E K Q L

ACTCAGTGTTCAATTTCCAGCCAATTTTCCTCACTACTTCAGCCCGGGTGAGAAGCAGCT

ACTGAGTGTTCAATTTCCAGCCAATTTTCCTCACTACTTCAGCCCGGGTGAGAAGCAGCT

*** ********************************************************

T G Q N P X

GACCGGCCAAAACCCGT

GACCGGCCAAAACCCGT

*****************

We have focused the argument on limitations to inferring an association between variant flanks on either side of too great a distance of identical common sequence. However, the argument in principle holds for insert-lengths that are so large that when one end derives from one variant region, the other extends beyond the opposite flank, thereby missing it entirely.
