# Supplementary material for: A deep transcriptomic resource for the copepod crustacean Labidocera madurae: A potential indicator species for assessing near shore ecosystem health
Source: PLoS One. 2017 Oct 24;12(10):e0186794. doi: 10.1371/journal.pone.0186794 (PMC5655441; doi:10.1371/journal.pone.0186794)
Supplement: S1 Table — For each stage three biological replicates were considered (R1, R2, R3). Number of pooled individuals (# ind), sequencing yields in number of reads (#) and number of megabases (Mb), are listed. For the mapping analysis overall alignment (%) and reads mapped > 1 time (%) are listed for each biological replicate. (DOCX) [file pone.0186794.s006.docx]

**S1 Table.**

|  | Adult female | | | Copepodite | | |
| --- | --- | --- | --- | --- | --- | --- |
|  | R1 | R2 | R3 | R1 | R2 | R3 |
| # Individuals | 5 | 6 | 6 | 20 | 26 | 15 |
| Raw reads | 85,901,661 | 86,553,097 | 86,586,788 | 92,094,836 | 90,779,877 | 86,084,082 |
| Sequencing Yields (Mb) | 14,490 | 14,720 | 14,730 | 15,530 | 15,300 | 14,740 |
| Overall alignment | 91.46 | 91.38 | 91.15 | 90.85 | 91.96 | 87.75 |
| Mapped > 1 time | 36.62 | 36.68 | 35.47 | 35.19 | 34.62 | 30.91 |
