# Supplementary material for: A deep transcriptomic resource for the copepod crustacean Labidocera madurae: A potential indicator species for assessing near shore ecosystem health
Source: PLoS One. 2017 Oct 24;12(10):e0186794. doi: 10.1371/journal.pone.0186794 (PMC5655441; doi:10.1371/journal.pone.0186794)
Supplement: S3 Table — I) Core clock proteins, II) Clock-associated proteins, III) Clock input pathway proteins and IV) Clock output pathway proteins. (DOCX) [file pone.0186794.s008.docx]

**S3 Table.**

**I. Core clock proteins**

***Labidocera maderae* clock (Labma-CLK)**

MSQRWSMSMDGMEELDDEKDETKRKSRNLSEKKRRDQFNMLVTELGGMVSSNNRKMDKTTVLKTTIAFLKQHNQQTVRSQAEEIKDDWKPPFLSNDEFTHLMLEALDGFIIVLGTDGHILYTSESLSSLLGYLPASLHNTTLYEILGDGDKIPLYNHLNMSGTIDTGNEQQLKIQLHIRRSVTADRIDGEEASPVLELVELSGYFRKWVNPESCQQEANYQSDDEISVKSGFSVQNSQVPASSRYRTFHADKTVFVATGRLVTPQLIRELPVVHQRETEFTSRHSLEWKFLFLDHRAPPIIGYLPFELLGTSGYDYYHVDDLDQVAGCHESLRQTGECTSCFYRFLTKGQQWIWLQSRYYITYHQWTSKPEFIVATHKVVNYKEVIDNANKKAGSERDSAAENKSGKTQSFRSGSPTWSSKSSLCGSTGTGFASSSRHSQDTGGNLQSSYSGYRSRQGGRSAQSTNSDVIDQFQLRQDNIIMSDTMSGMDVNSEIAAPIESPLVLSPPQLQLQQQLRTKHAELSRRILLQQQELARLGEQLLLTPQSLGQATAVSVQSYPPQQQQQQQQQHIQVQQSQQRQPIISGGNPT+

***Labidocera maderae* cryptochrome 2 (Labma-CRY2)**

MSRKSSRRKHSVHWFRKGLRLSDNPALLRAIRRCETFRCIFILDPWFAGSSNVGTNKWRFLLQCLEDLDSSLRKLNSRLFVVRGQPADVLPTLFKEWGITYFTFEEDPEPFGRVRDQNIIAMCKEMGIIVTKEHSHTLYNLDRIIERNNGKAPLTYKRFQSIIEGMDPPPKPVPTLTLQMLGGGVSPIADDHDEKYGVPSLEELGFDCENLKPTVWVGGETEALSRLERHLERKAWVASFGRPKMTPQSLLASQTGLSPYLRFGCLSTRLFYHALTDLYRKIKKCDPPLSLHGQLLWREFFYCASTKNPNFDKMVGNPICVQIPWDRNDEALAKWANAQTGFPWIDAIMTQLREEGWIHHLARHAVACFLTRGDLWVSWEEGMKVFEELLLDADWSVNAGTWLWLSCSSFFQQFFHCYCPVKFGRKADANGDFIRRYLPVLKNYPTRYIHEPWTAPEAVQKSAKCIIGQDYPKPMCNHSFVSKINMERMKQVYQQLAHYSQTGQTGPGIPQDLLNQLPKVPKMLSPTSGAMGPPDPPENKKKTSGEKEDMEGKNLISSQQNFYPSNVQRTATNQYAISGPRTVKFAVTPGAEHPTSAASGSLASTPTTTNLTYPPFDTSSNIYMQNNRPGYSFEQENIQSSSRQESLPLLSGDMLLPLSVPLSHHQVLKLAQPVLDADLDIPPPTPSLPSSISTVHQGDLPLMTSGEVASQLRASEVPNHRTVSSVGADISMSSANISMSSANISMNSDSVSMSSGQQLGISSLVADCGDIQLNQNIVHSIPGYEAAIPEEYYPSVSQQ

*Labidocera maderae* cycle variant 1 (Labma-CYC-v1)

MNQAGESQPDYTASHDGFHYQNLVFPDFLHEGPGILNMMEFQGYSEDLATSPGHSEKKRKLPPEEDTHLPEETKQRKILSQDVSPLKEEQKRYVRQNHSEIEKRRRDKMNTYITELSSMIPTCVAMQRKMDKLTVLRLAVQHLKSIRGSLDAYSEGNSRPAMLTDSELRQLIVPSADGFIFVVDSARTRILYVSESVNNILNFSSQDLIGQSLFDILHPKDIDKVKEQLSNSDGPRSRLIDSKTMLPLKVGEIPQSIGRLQPGARRVFFCRMKCKPTQMIKQEDDIYPILNSNSVQPQENNGKKKKCGNSDKKYISIQCTGYLKSWPFTKVGLEGEFPDLDADSDTCMSCLVAVGRVQPSFQSTIEDCIERGETTSTAVEFFSRHGIDGKFSFVDQRVTLMLGYLPQELVGTSLYEHIQYDDIPLIAECHRKSLRNSDEVNTPIFGFRTKDGNFVKLKSKFKHFRNPWTREIDYIFCKNYLIFSKEKYSESSNFGSADTADMDFIMANNSGRHNGGTQTGAGNGGNSGVGANNGQIGKDIQQVISSHAEAAKIGRNIADEEIEKWRTDNSASNSPISSLQSGSPSTLQSSTSINPASSALGGKGEAELIREAMLDVERDRNKLSGVIVSRNSSTSNATLNRLVTNRARIDQIPAAALSARTSPPHSTSSSESGNDEAATAVLMSLLEAEGGLGGPFDFGSLPWPLP

*Labidocera maderae* cycle variant 2a (Labma-CYC-v2a)

MNQAGESQPDYTASHDGFHYQNLVFPDFLHEGPGILNMMEFQGYSEDLATSPGHSEKKRKLPPEEDTHLPEETKQRKILSQEEQKRYVRQNHSEIEKRRRDKMNTYITELSSMIPTCVAMQRKMDKLTVLRLAVQHLKSIRGSLDAYSEGNSRPAMLTDSELRQLIVPSADGFIFVVDSARTRILYVSESVNNILNFSSQDLIGQSLFDILHPKDIDKVKEQLSNSDGPRSRLIDSKTMLPLKVGEIPQSIGRLQPGARRVFFCRMKCKPTQMIKQEDDIYPILNSNSVQPQENNGKKKKCGNSDKKYISIQCTGYLKSWPFTKVGLEGEFPDLDADSDTCMSCLVAVGRVQPSFQSTIEDCIERGETTSTAVEFFSRHGIDGKFSFVDQRVTLMLGYLPQELVGTSLYEHIQYDDIPLIAECHRKSLRNSDEVNTPIFGFRTKDGNFVKLKSKFKHFRNPWTREIDYIFCKNYLIFSKEKYSESSNFGSADTADMDFIMANNSGRHNGGTQTGAGNGGNSGVGANNGQIGKDIQQVISSHAEAAKIGRNIADEEIEKWRTDNSASNSPISSLQSGSPSTLQSSTSINPASSALGGKGEAELIREAMLDVERDRNKLSGVIVSRNSSTSNATLNRLVTNRARIDQIPAAALSARTSPPHSTSSSESGNDEAATAVLMSLLEAEGGLGGPFDFGSLPWPLP

*Labidocera maderae* cycle variant 2b (Labma-CYC-v2b)

MNQAGESQPDYTASHDGFHYQNLVFPDFLHEGPGILNMMEFQGYSEDLATSPGHSEKKRKLPPEEDTHLPEETKQRKILSQEEQKRYVRQNHSEIEKRRRDKMNTYITELSSMIPTCVAMQRKMDKLTVLRLAVQHLKSIRGSLDAYSEGNSRPAMLTDSELRQLIVPSADGFIFVVDSARTRILYVSESVNNILNFSSQDLIGQSLFDILHPKDIDKVKEQLSNSDGPRSRLIDSKTMLPLKVGEIPQSIGRLQPGARRVFFCRMKCKPTQMIKQEDDIYPILNSNSVQPQENNGKKKKCGNSDKKYISIQCTGYLKSWPFTKVGLEGEFPDLDADSDTCMSCLVAVGRVQPSFQSTIEDCIERGETTSTAVEFFSRHGIDGKFSFVDQRVTLMLGYLPQELVGTSLYEHIQYDDIPLIAECHRKSLRNSDEVNTPIFGFRTKDGNFVKLKSKFKHFRNPWTREIDYIFCKNYLIFSKEKYSESSNFGSADTADMDFIMANNSGRPNGGTQTGAGHGGNSGGGANNGQIGKDIQQVISSHAEAAKIGRNIADEEIEKWRTDNSASNSPISSLQSGSPSTLQSSTSINPASSALGGKGEAELIREAMLDVERDRNKLSGVIVSRNSSTSNATLNRLVTNRARIDQIPAAALSARTSPPHSTSSSESGNDEAATAVLMSLLEAEGGLGGPFDFGSLPWPLP

*Labidocera maderae* cycle variant 3 (Labma-CYC-v3)

MMEFQGYSEDLATSPGHSEKKRKLPPEEDTHLPEETKQRKILSQDVSPLKEEQKRYVRQNHSEIEKRRRDKMNTYITELSSMIPTCVAMQRKMDKLTVLRLAVQHLKSIRGSLDAYSEGNSRPAMLTDSELRQLIVPSADGFIFVVDSARTRILYVSESVNNILNFSSQDLIGQSLFDILHPKDIDKVKEQLSNSDGPRSRLIDSKTMLPLKVGEIPQSIGRLQPGARRVFFCRMKCKPTQMIKQEDDIYPILNSNSVQPQENNGKKKKCGNSDKKYISIQCTGYLKSWPFTKVGLEGEFPDLDADSDTCMSCLVAVGRVQPSFQSTIEDCIERGETTSTAVEFFSRHGIDGKFSFVDQRVTLMLGYLPQELVGTSLYEHIQYDDIPLIAECHRKSLRNSDEVNTPIFGFRTKDGNFVKLKSKFKHFRNPWTREIDYIFCKNYLIFSKEKYSESSNFGSADTADMDFIMANNSGRHNGGTQTGAGNGGNSGVGANNGQIGKDIQQVISSHAEAAKIGRNIADEEIEKWRTDNSASNSPISSLQSGSPSTLQSSTSINPASSALGGKGEAELIREAMLDVERDRNKLSGVIVSRNSSTSNATLNRLVTNRARIDQIPAAALSARTSPPHSTSSSESGNDEAATAVLMSLLEAEGGLGGPFDFGSLPWPLP

*Labidocera maderae* cycle variant 4 (Labma-CYC-v4)

MMEFQGYSEDLATSPGHSEKKRKLPPEEDTHLPEETKQRKILSQEEQKRYVRQNHSEIEKRRRDKMNTYITELSSMIPTCVAMQRKMDKLTVLRLAVQHLKSIRGSLDAYSEGNSRPAMLTDSELRQLIVPSADGFIFVVDSARTRILYVSESVNNILNFSSQDLIGQSLFDILHPKDIDKVKEQLSNSDGPRSRLIDSKTMLPLKVGEIPQSIGRLQPGARRVFFCRMKCKPTQMIKQEDDIYPILNSNSVQPQENNGKKKKCGNSDKKYISIQCTGYLKSWPFTKVGLEGEFPDLDADSDTCMSCLVAVGRVQPSFQSTIEDCIERGETTSTAVEFFSRHGIDGKFSFVDQRVTLMLGYLPQELVGTSLYEHIQYDDIPLIAECHRKSLRNSDEVNTPIFGFRTKDGNFVKLKSKFKHFRNPWTREIDYIFCKNYLIFSKEKYSESSNFGSADTADMDFIMANNSGRHNGGTQTGAGNGGNSGVGANNGQIGKDIQQVISSHAEAAKIGRNIADEEIEKWRTDNSASNSPISSLQSGSPSTLQSSTSINPASSALGGKGEAELIREAMLDVERDRNKLSGVIVSRNSSTSNATLNRLVTNRARIDQIPAAALSARTSPPHSTSSSESGNDEAATAVLMSLLEAEGGLGGPFDFGSLPWPLP

*Labidocera maderae* period variant 1 (Labma-PER-v1)

MGEENDKKGNQKQDNDKEEKDGEITENSSSMYKGSSELSTNQQDSAYSSIINSAYSSLDSLRTPTTNSCASLSQSTNSRSSTKILSSKSSGSSGRSTQLGAVTSTTQASAQGNCKDLVKPSTKETPDRPLRKKKPKISHSNDESRQFTTEKGNSSGFNTAQECKAAKVATLSQALGYVDQFRRIQASKSDKARRGHDESDLNDLAINLKVASQTASTPTIIGMLPEAPDERVKIPEDTIIKHEKTEGSGFCVAVSLHDGMVMQTTVSITSILGYPKDMWVGRSFIDFVHPQDKDTFINQVTENIGLILRDFHPTQGTRKTKKDTYCKSGGFFCRIRIYNGLKSGFSVKERKTRYSPFKLSVCFLEMDSKDSSRTPGSGSIDGGPQSTYLFITAIPLVSAFTESYQEFPANERNSEQHVFVTKHNSSCVFSNIEESTIPYLGYLPQDMNGEDIFNFIHPHDLGQLKNVFETAMLEQGKPCKSKDIRFKVRNGGYIQCNSWWSCFINPWSRQLEFVHGKHVVTKGPRHPDVFADFLKDSAETVQLTEEHTKQTANINNEIKHILRKTVQRNTFLDSNTENSSKTKKELSSFMGTLLEEVSKAETIKLSRTGKIGAVVIGNISPHQSDSSETPPSYNQLTYNENLTRFFNSQPKTLSEKDNQDVSSQHQSKSSEDTLQESNHLKKKGQKPCSQRSVHKEDGGRSALGSGGSGEQTQTISGSGEGNGNHSSTLQTGLSAGQVQSGREECMMSQDGSGSGSRFGSGSRGASVDAYNPPALTVELLAMHNKDMEQKMLSKFKEAKKTGDIRFIRDSKYGPSQNNVVKRLSLQKGNPSVLKNETQIKSKKHQSKPAKEPKSGPAYDLQPNCLPGTDLQWNHFQEEVGARNKFSNAKVNHGHEGYVGFNQANMMERGTPNTLPGFRNVIGVPAFYMAINGRLNSQPAQTFDSVQSGILLQNTPTRYIPMGVIESVPGGVYHQLGISGVDPRVPGSKRVVGGHHSPSQQPELPNCLAVNSCMVCHPASDRSSCKVLKHETKFARPLSRTGSRATSVKGEPGSALESNASATASVKEKNILSPKGHTKPAKKGYSNENDIDPNCLSTSSSSLYSFLKTSEDYSCTNPNSSGDEVIQLNKHIAKPVLSEPFWNERIELTTDLIFNYQLEPRDKATVLKEDLEKLRNMQQFPELHEQLMELFLETEETMSHAQLLLEVNSNSSYEESTSAEEGAGEYDEIAYKKKRLKRKEKLEKLNIFMEADAPFPMPDSPKLALSSKGSWKMFSSPNYSSNADHNSEFSISSNSEVDCIKNVCNKGQESVQENTKVLAKGKEEDEQKADQETGTPGDQEAGKPEEEDTNQSKQKEEQEGRDHDDKDVSMESVQDPAAGCVQGPQLLNNESSSQPVSQGMENDNSGDSDL

*Labidocera maderae* period variant 2 (Labma-PER-v2)

MGEENDKKGNQKQDNDKEEKDGEITENSSSMYKGSSELSTNQQDSAYSSIINSAYSSLDSLRTPTTNSCASLSQSTNSRSSTKILSSKSSGSSGRSTQLGAVTSTTQGNCKDLVKPSTKETPDRPLRKKKPKISHSNDESRQFTTEKGNSSGFNTAQECKAAKVATLSQALGYVDQFRRIQASKSDKARRGHDESDLNDLAINLKVASQTASTPTIIGMLPEAPDERVKIPEDTIIKHEKTEGSGFCVAVSLHDGMVMQTTVSITSILGYPKDMWVGRSFIDFVHPQDKDTFINQVTENIGLILRDFHPTQGTRKTKKDTYCKSGGFFCRIRIYNGLKSGFSVKERKTRYSPFKLSVCFLEMDSKDSSRTPGSGSIDGGPQSTYLFITAIPLVSAFTESYQEFPANERNSEQHVFVTKHNSSCVFSNIEESTIPYLGYLPQDMNGEDIFNFIHPHDLGQLKNVFETAMLEQGKPCKSKDIRFKVRNGGYIQCNSWWSCFINPWSRQLEFVHGKHVVTKGPRHPDVFADFLKDSAETVQLTEEHTKQTANINNEIKHILRKTVQRNTFLDSNTENSSKTKKELSSFMGTLLEEVSKAETIKLSRTGKIGAVVIGNISPHQSDSSETPPSYNQLTYNENLTRFFNSQPKTLSEKDNQDVSSQHQSKSSEDTLQESNHLKKKGQKPCSQRSVHKEDGGRSALGSGGSGEQTQTISGSGEGNGNHSSTLQTGLSAGQVQSGREECMMSQDGSGSGSRFGSGSRGASVDAYNPPALTVELLAMHNKDMEQKMLSKFKEAKKTGDIRFIRDSKYGPSQNNVVKRLSLQKGNPSVLKNETQIKSKKHQSKPAKEPKSGPAYDLQPNCLPGTDLQWNHFQEEVGARNKFSNAKVNHGHEGYVGFNQANMMERGTPNTLPGFRNVIGVPAFYMAINGRLNSQPAQTFDSVQSGILLQNTPTRYIPMGVIESVPGGVYHQLGISGVDPRVPGSKRVVGGHHSPSQQPELPNCLAVNSCMVCHPASDRSSCKVLKHETKFARPLSRTGSRATSVKGEPGSALESNASATASVKEKNILSPKGHTKPAKKGYSNENDIDPNCLSTSSSSLYSFLKTSEDYSCTNPNSSGDEVIQLNKHIAKPVLSEPFWNERIELTTDLIFNYQLEPRDKATVLKEDLEKLRNMQQFPELHEQLMELFLETEETMSHAQLLLEVNSNSSYEESTSAEEGAGEYDEIAYKKKRLKRKEKLEKLNIFMEADAPFPMPDSPKLALSSKGSWKMFSSPNYSSNADHNSEFSISSNSEVDCIKNVCNKGQESVQENTKVLAKGKEEDEQKADQETGTPGDQEAGKPEEEDTNQSKQKEEQEGRDHDDKDVSMESVQDPAAGCVQGPQLLNNESSSQPVSQGMENDNSGDSDL

*Labidocera maderae* timeless variant 1 (Labma-TIM-v1)

MMDFNFEEPTALLGSQVGDVYQLHENTAEVLNDMNRRLNEEEKTLRTYRRALAFSQVVQKDLIPILMNCSDRGDVFDSVIRLLVNLSVPIECLCPIDIMSQSTNGMQVIFELKNSLKNTKEAFTDSRSSKSIMEKLSSLTENKAKELCYSDINTINSCLLLIRNILHIPETSARPGKSNLQNQIMWNLFVQNLDKVLLDLISHVNASHWCTVIVQLIALIYKDQHIVNLQKMLQTFIENTLSESSDNESNTSPLIFFQHGDSSSQAMDSSEEDSRGQRTTSANSGSDCSSPPIASPHSHNSEEKTEEINLHEETPEEKPEPEKKRESSSGRAMETSSGIESESEPPLKKFIQEENSVIHAKLEPRPEEDFQHKQRFQCIHPISGGRSVFRSESQQGNSSESSDITGVPKSTKNTCGSNSDFGYVSQQPCVNDIPESNSSSSNEEERRIKRQASRANIVKPKAKNQTTLTHQEKQEKRRLKMLSLSRQQRMRVKAMVNHSPTDEDISELLKEFTVDFILTEYSNLVRELLKLECGRQEGLNLDKSHFLWLITYFLKFASQLDIGLEHMGSVLSFNTIAYITYEGVVQQETLELANRGRKSDMTPHLRRMHLVVTALREFIQTLTAYKQSIYTSEGDERHLFSVQLQISYTKSLRQLMVLLLRTYDSGLHSQQYLSDLVVTNHHILTNIEEISQSEMYQGPRVDLLDHLKQLANADIMRHYGHLLTDFQDNLPLVNDCLFTMMHHVAGDLEAPEVLFIPVVLQTFSRILEQGLNICQDWVDLIEFIIQKFIQTMQIAPQKCAATIADCMDSAEVADECGLTGTQASHLFYNYNQVENHSDPVGAIIEIYRQTDTVTFSRMSIIQALLSHGIITHAQYMNFMYMKSVLAQCKTERDGSVIAEVGSEHCTSDGHITDGEEQQDGIIREHKEIKVLKDCLIKQGKESLIYWVQEVLLDACRVKMYPDSICVENRSVPHEPIYFHYMLAKQSIPLVPFNRVQWQGLQTEAFILLLHKLGFLLPADVGKVYPRIPFFWSADHLYAMASKLGPVKEDPFKFSPEDIERIRRKEDRFSVKSPVGDRPPPEKDCDENHDLAELDLLAGNESSNSNMDSRKWIYMTMASKKMEKPSSQKQKDETDMETEEAPKEKEVEKESVTARCPPGRQDSDPLLEESMDET

*Labidocera maderae* timeless variant 2 (Labma-TIM-v2)

MMDFNFEEPTALLGSQVGDVYQLHENTAEVLNDMNRRLNEEEKTLRTYRRALAFSQVVQKDLIPILMNCSDRGDVFDSVIRLLVNLSVPIECLCPIDIMSQSTNGMQVIFELKNSLKNTKEAFTDSRSSKSIMEKLSSLTENKAKELCYSDINTINSCLLLIRNILHIPETSARPGKSNLQNQIMWNLFVQNLDKVLLDLISHVNASHWCTVIVQLIALIYKDQHIVNLQKMLQTFIENTLSESSDNESNTSPLHGDSSSQAMDSSEEDSRGQRTTSANSGSDCSSPPIASPHSHNSEEKTEEINLHEETPEEKPEPEKKRESSSGRAMETSSGIESESEPPLKKFIQEENSVIHAKLEPRPEEDFQHKQRFQCIHPISGGRSVFRSESQQGNSSESSDITGVPKSTKNTCGSNSDFGYVSQQPCVNDIPESNSSSSNEEERRIKRQASRANIVKPKAKNQTTLTHQEKQEKRRLKMLSLSRQQRMRVKAMVNHSPTDEDISELLKEFTVDFILTEYSNLVRELLKLECGRQEGLNLDKSHFLWLITYFLKFASQLDIGLEHMGSVLSFNTIAYITYEGVVQQETLELANRGRKSDMTPHLRRMHLVVTALREFIQTLTAYKQSIYTSEGDERHLFSVQLQISYTKSLRQLMVLLLRTYDSGLHSQQYLSDLVVTNHHILTNIEEISQSEMYQGPRVDLLDHLKQLANADIMRHYGHLLTDFQDNLPLVNDCLFTMMHHVAGDLEAPEVLFIPVVLQTFSRILEQGLNICQDWVDLIEFIIQKFIQTMQIAPQKCAATIADCMDSAEVADECGLTGTQASHLFYNYNQVENHSDPVGAIIEIYRQTDTVTFSRMSIIQALLSHGIITHAQYMNFMYMKSVLAQCKTERDGSVIAEVGSEHCTSDGHITDGEEQQDGIIREHKEIKVLKDCLIKQGKESLIYWVQEVLLDACRVKMYPDSICVENRSVPHEPIYFHYMLAKQSIPLVPFNRVQWQGLQTEAFILLLHKLGFLLPADVGKVYPRIPFFWSADHLYAMASKLGPVKEDPFKFSPEDIERIRRKEDRFSVKSPVGDRPPPEKDCDENHDLAELDLLAGNESSNSNMDSRKWIYMTMASKKMEKPSSQKQKDETDMETEEAPKEKEVEKESVTARCPPGRQDSDPLLEESMDET

*Labidocera maderae* timeless variant 3 (Labma-TIM-v3)

MMDFNFEEPTALLGSQVGDVYQLHENTAEVLNDMNRRLNEEEKTLRTYRRALAFSQVVQKDLIPILMNCSDRGDVFDSVIRLLVNLSVPIECLCPIDIMSQSTNGMQVIFELKNSLKNTKEAFTDSRSSKSIMEKLSSLTENKAKELCYSDINTINSCLLLIRNILHIPETSARPGKSNLQNQIMWNLFVQNLDKVLLDLISHVNASHWCTVIVQLIALIYKDQHIVNLQKMLQTFIENTLSESSDNESNTSPLIFFQHGDSSSQAMDSSEEDSRGQRTTSANSGSDCSSPPIASPHSHNSEEKTEEINLHEETPEEKPEPEKKRESSSGRAMETSSGIESESEPPLKKFIQEENSVIHAKLEPRPEEDFQHKQRFQCIHPISGNSSESSDITGVPKSTKNTCGSNSDFGYVSQQPCVNDIPESNSSSSNEEERRIKRQASRANIVKPKAKNQTTLTHQEKQEKRRLKMLSLSRQQRMRVKAMVNHSPTDEDISELLKEFTVDFILTEYSNLVRELLKLECGRQEGLNLDKSHFLWLITYFLKFASQLDIGLEHMGSVLSFNTIAYITYEGVVQQETLELANRGRKSDMTPHLRRMHLVVTALREFIQTLTAYKQSIYTSEGDERHLFSVQLQISYTKSLRQLMVLLLRTYDSGLHSQQYLSDLVVTNHHILTNIEEISQSEMYQGPRVDLLDHLKQLANADIMRHYGHLLTDFQDNLPLVNDCLFTMMHHVAGDLEAPEVLFIPVVLQTFSRILEQGLNICQDWVDLIEFIIQKFIQTMQIAPQKCAATIADCMDSAEVADECGLTGTQASHLFYNYNQVENHSDPVGAIIEIYRQTDTVTFSRMSIIQALLSHGIITHAQYMNFMYMKSVLAQCKTERDGSVIAEVGSEHCTSDGHITDGEEQQDGIIREHKEIKVLKDCLIKQGKESLIYWVQEVLLDACRVKMYPDSICVENRSVPHEPIYFHYMLAKQSIPLVPFNRVQWQGLQTEAFILLLHKLGFLLPADVGKVYPRIPFFWSADHLYAMASKLGPVKEDPFKFSPEDIERIRRKEDRFSVKSPVGDRPPPEKDCDENHDLAELDLLAGNESSNSNMDSRKWIYMTMASKKMEKPSSQKQKDETDMETEEAPKEKEVEKESVTARCPPGRQDSDPLLEESMDET

*Labidocera maderae* timeless variant 4 (Labma-TIM-v4)

MMDFNFEEPTALLGSQVGDVYQLHENTAEVLNDMNRRLNEEEKTLRTYRRALAFSQVVQKDLIPILMNCSDRGDVFDSVIRLLVNLSVPIECLCPIDIMSQSTNGMQVIFELKNSLKNTKEAFTDSRSSKSIMEKLSSLTENKAKELCYSDINTINSCLLLIRNILHIPETSARPGKSNLQNQIMWNLFVQNLDKVLLDLISHVNASHWCTVIVQLIALIYKDQHIVNLQKMLQTFIENTLSESSDNESNTSPLHGDSSSQAMDSSEEDSRGQRTTSANSGSDCSSPPIASPHSHNSEEKTEEINLHEETPEEKPEPEKKRESSSGRAMETSSGIESESEPPLKKFIQEENSVIHAKLEPRPEEDFQHKQRFQCIHPISGNSSESSDITGVPKSTKNTCGSNSDFGYVSQQPCVNDIPESNSSSSNEEERRIKRQASRANIVKPKAKNQTTLTHQEKQEKRRLKMLSLSRQQRMRVKAMVNHSPTDEDISELLKEFTVDFILTEYSNLVRELLKLECGRQEGLNLDKSHFLWLITYFLKFASQLDIGLEHMGSVLSFNTIAYITYEGVVQQETLELANRGRKSDMTPHLRRMHLVVTALREFIQTLTAYKQSIYTSEGDERHLFSVQLQISYTKSLRQLMVLLLRTYDSGLHSQQYLSDLVVTNHHILTNIEEISQSEMYQGPRVDLLDHLKQLANADIMRHYGHLLTDFQDNLPLVNDCLFTMMHHVAGDLEAPEVLFIPVVLQTFSRILEQGLNICQDWVDLIEFIIQKFIQTMQIAPQKCAATIADCMDSAEVADECGLTGTQASHLFYNYNQVENHSDPVGAIIEIYRQTDTVTFSRMSIIQALLSHGIITHAQYMNFMYMKSVLAQCKTERDGSVIAEVGSEHCTSDGHITDGEEQQDGIIREHKEIKVLKDCLIKQGKESLIYWVQEVLLDACRVKMYPDSICVENRSVPHEPIYFHYMLAKQSIPLVPFNRVQWQGLQTEAFILLLHKLGFLLPADVGKVYPRIPFFWSADHLYAMASKLGPVKEDPFKFSPEDIERIRRKEDRFSVKSPVGDRPPPEKDCDENHDLAELDLLAGNESSNSNMDSRKWIYMTMASKKMEKPSSQKQKDETDMETEEAPKEKEVEKESVTARCPPGRQDSDPLLEESMDET

**II. Clock-associated proteins**

*Labidocera maderae* casein kinase IIα (Labma-CKIIα)

MPLASRARVYADVNSHRPREYWDYEAHGVDWGNQDDYQLVRKLGRGKYSEVFESINITTNEKCVVKTLKPVKKKKIKREIKILENLRGGTNVITLHGVVKDPVSRTPALIFEHVNNTDFKQLYQTLTDYDIRGYLYELLRALDYCHSMGIMHRDVKPHNVMIDHENRRLRLIDWGLAEFYHPGQEYNVRVASRYFKGPELLVDYQMYDYSLDMWSLGCMLASMIFRKEPFFHGHDNYDQLVRIAKVLGTEELYEYLDKYQIELDPRFSDILGRHSRKRWERFVHSENQHLVSPETLDFLDKLLRYDHQERLTAMEAMEHAYFYPVVKDHGRISNISNSPTGGQGLGPGSIPQQVPSSPILSPNNTPLPSNQQTQQ

*Labidocera maderae* casein kinase IIα (Labma-CKIIβ)

MSSSEEVSWISWFCGLRGNEFFCEVDEDYIQDKFNLTGLNEQVPHYRQALDMILDLEPDDELEDNPNQSDLIEQAAEMLYGLIHARYILTNRGIAQMIEKYQAGDFGHCPRVYCENQPMLPIGLSDVPGEAMVKLYCPKCMDVYTPKSSRHHHTDGAYFGTGFPHMLFMVHPEYRPKRPANQFVPRLYGFKIHPLAYQIQQQAAANFKTHETKPGRR

*Labidocera maderae* clockwork orange variant 1 (Labma-CWO-v1)

MQSDSREEDGMNDSSWTTPISYHPSSSSSSSFVPSSSSSFIPSSSPSFIPSSGSSFVSSRVPTSYGETLLSHPKSKKSSSPGSGQANCDEMSNTMNDMMTRRKDLPEDLACFLQQQKQRQARLETTMRSFPSTYDVDQDKQDLKRKRNSHEDPLSHRIIEKRRRDRMNSCLADLSRLIPTSYTKKGRGRIEKTEIIEMAIKHIKHMQSHACREEGQQDARGKCDLSSEMASNLERSNNLESFRVGYHECLTETMHFLVEKEGLYSGDAICVRLMSHLQKHYDQLGKASTSEILTAVTRKWIGSTSIPQPQKGGSRDRGEDAVKSENSEESGYFSVKQEEDASEVVNLTTGHQKQEQSGSLSETYTGGREEREPSVYRRRSTSAEQYSRTDDCTQRREEEGPRGELTQEQPSDFSRKGEADGSDSFQSNLYKFKTNIRQRFDMQDHGVMGRDRMSSINSEPDPSDGMSDRKRLSLDPSENLGVEDMRRLSGDLGRPTVHQLPGPLNEPRHQDDLCPRSPPPPPVRTSTRSELSPAPVKNASQTVPIFALNSRGSYYVPMTVDISIISPFMSVFTEESCPILHPVTISVNFQSPHIPPARSAVIQQASVIKHWRDQPGI

*Labidocera maderae* clockwork orange variant 2 (Labma-CWO-v2)

MQSDSREEDGMNDSSWTTPISYHPSSSSSSSFVPSSSSSFIPSSSPSFIPSSGSSFVSSRVPTSYGETLLSHPKSKKSSSPGSGQANCDEMSNTMNDMMTRRKDLPEDLACFLQQQKQRQARLETTMRSFPSTYDVDQDKQDLKRKRNSHEDPLSHRIIEKRRRDRMNSCLADLSRLIPTSYTKKGRGRIEKTEIIEMAIKHIKHMQSHACREEGKCDLSSEMASNLERSNNLESFRVGYHECLTETMHFLVEKEGLYSGDAICVRLMSHLQKHYDQLGKASTSEILTAVTRKWIGSTSIPQPQKGGSRDRGEDAVKSENSEESGYFSVKQEEDASEVVNLTTGHQKQEQSGSLSETYTGGREEREPSVYRRRSTSAEQYSRTDDCTQRREEEGPRGELTQEQPSDFSRKGEADGSDSFQSNLYKFKTNIRQRFDMQDHGVMGRDRMSSINSEPDPSDGMSDRKRLSLDPSENLGVEDMRRLSGDLGRPTVHQLPGPLNEPRHQDDLCPRSPPPPPVRTSTRSELSPAPVKNASQTVPIFALNSRGSYYVPMTVDISIISPFMSVFTEESCPILHPVTISVNFQSPHIPPARSAVIQQASVIKHWRDQPGI

*Labidocera maderae* doubletime I (Labma-DBT-I)

MELRVGNKYRLGRKIGSGSFGDIYLGTNISTGEEVAIKLECIKTKHPQLHIESKFYRIMQGGVGIPAIKWCGSEGDYNVMVMELLGPSLEDLFNFCSRRFSLKTVLLLADQLISRIEYIHSKNFIHRDIKPDNFLMGLGKKGNLVYIIDFGLAKKYRDARTHQHILYRENKNLTGTARYASINTHLGIEQSRRDDMESLGYILMYFVQGTLPWQGLRAATKQQKYERISEKKMSTPIDVLCKGAPCEFATYLNYCRSLRFDEKPDYSYLRQLFRNLFHRQGFTYDYVFDWNMLKFGGSRGPETDGDPRYSRK

*Labidocera maderae* doubletime II variant 1 (Labma-DBT-II-v1)

MSKNLSVPPARAKSPAPKTKKMMTRKTRDKVTPRAKSRSPNPSFCSVLSRVKDWELGAFTPKTALAENVDILNPSNTPYSEPDNDCSPGNDTDWDWGQKDSPGDSPGPSDNPALTKDALSGTSTPVGELDIRIAGKYRLGRKIGGGSFGDIYLATDVNTNEEVAVKLEHVKTKHPQLHVECKFYKVMQGGVGIPVVNYYGTEGEYNVMVMELLGPSLEDLFNFCNRKLSLKTVLLLADQLICRIEFIHGKNFIHRDMKPDNFLMGLGKKGNLVYVIDFGLAKKFRDQRTHQHIPYRENKNLTGTARYTSINTHLGIEQSRRDDMEALGYILIYFLQGTLPWQGLRAKTKAQKYEKISEKKLSTPVEELCAGAPAEFATYHNYVRSLRFEEKPDYAYLRQLIRNLFYRQGFTYDYVFDWNTLKDDKNPSVAKLEGGSESDKKNGDGDNDREKKKINEEDTNPVEIPTAATTTGATSAVAPTVKAQTEILNHHVAEEPGPEGEPEKPVPAGDGSASIQKPPQIISSPPAFHLDHNTNFISSKPVDVPIVNGFTEPPVILLTTCTPLASPRPEYNSLQPPQLLSPSQQHNQLNSSAQSLVSSTSSVKQLQVL

*Labidocera maderae* doubletime II variant 2 (Labma-DBT-II-v2)

MSKNLSVPPARAKSPAPKTKKMMTRKTRDKVTPRAKSRSPNPSFCSVLSRVKDWELGAFTPKTALAENVDILNPSNTPYSEPDNDCSPGNDTDWDWGQKDSPGDSPGPSDNPALTKDALSGTSTPVGELDIRIAGKYRLGRKIGGGSFGDIYLATDVNTNEEVAVKLEHVKTKHPQLHVECKFYKVMQGGVGIPVVNYYGTEGEYNVMVMELLGPSLEDLFNFCNRKLSLKTVLLLADQLICRIEFIHGKNFIHRDMKPDNFLMGLGKKGNLVYVIDFGLAKKFRDQRTHQHIPYRENKNLTGTARYTSINTHLGIEQSRRDDMEALGYILIYFLQGTLPWQGLRAKTKAQKYEKISEKKLSTPVEELCAGAPAEFATYHNYVRSLRFEEKPDYAYLRQLIRNLFYRQGFTYDYVFDWNTLKDDKNPSVAKLEGGSESDKKNGDGDNDREKKKINEEDTNPVEIPTAATTTGATSAVAPTVKAQTEILNHHVAEEPGPEGEPEKPVPAGDGSASIQKPPQIISSPPAFHLDHNTNFTCTPLASPRPEYNSLQPPQLLSPSQQHNQLNSSAQSLVSSTSSVKQLQVL

*Labidocera maderae* doubletime III variant 1 (Labma-DBT-III-v1)

MIVGHKYDIGRKIGSGSFGDIYIGSNILSGEEVAIKLEPIIAKHPQLEYETRVYRALAGGVGIPFVRWFGHEGDFNAMVIDLLGPSLEDLFNFCTRKFSLKTVLLLADQLLSRIEYIHSKSFIHRDIKPDNFLMGLGRRGNLVNVIDFGLAKKYRDARTHLHIPYRENKNLTGTARYASINTHLGIEQSRRDDMESLGYVLMYFCRGSLPWQGLKAKNKKQKYERILEKKMMTPAEVLCRGYPREFAVYLNYSRSLRFEDRPDYAFLRRLFRELFVREGYTYDYVFDWTILKYNQQQKGTGMAEMLPENPRADGNQTAADGNNNNNTNKVTRLVKEMSGAGSGTAMGNTSAQAVAGSSRPVGVSNVYEQASPRDGGRNQRTDQPRFQAVAPARFPSGSPSADKMMGGYGTNPV

*Labidocera maderae* doubletime III variant 2 (Labma-DBT-III-v2)

MIVGHKYDIGRKIGSGSFGDIYIGSNILSGEEVAIKLEPIIAKHPQLEYETRVYRALAGGVGIPFVRWFGHEGDFNAMVIDLLGPSLEDLFNFCTRKFSLKTVLLLADQLLSRIEYIHSKSFIHRDIKPDNFLMGLGRRGNLVNVIDFGLAKKYRDARTHLHIPYRENKNLTGTARYASINTHLGIEQSRRDDMESLGYVLMYFCRGSLPWQGLKAKNKKQKYERILEKKMMTPAEVLCRGYPREFAVYLNYSRSLRFEDRPDYAFLRRLFRELFVREGYTYDYVFDWTILKYNQQQKGTGMAEMLPENPRADGNQTAADGNNNNNTNKVTRLVKEMSGAGSGTAMGNTSAQAVAGSSRPVGVSNVYEQASPRDGGRNQRTDQPRFQAVAPARFPSGSPSADKVMLG

*Labidocera maderae* jetlag (Labma-JET)

MKDLCLLDLSWTDILFPNIIPLLRIEDLFGLRCVSKEFLIMVEQYFAQNRKLDLSVLRKHTEQAFKIMTSNATCLRYLNLSGSKVATDDLVREILMSNPLLVYLNLSNCHHCTSGILQTMTIKNRQIERLILQDCHWVTRESIEYHAHHQGFSEGKTNQSRLVEVNFTGCWELTDDILVDFLARFKKLKVVQLGNIYSLTDMIMRALATYTRDLEYLDIRGCWRISDNGLRLVTEYCRNLKRLAIMDCRGITERSLKKLRDNKVQIDRKLDETLLRIERMRLQYMHDRLPV

*Labidocera maderae* PAR-domain protein 1 I variant 1 (Labma-PDP1-I-v1)

MHRYNLIRPANIDTLKEQQVPIRPPIMHHVGKPPELYSHTMQDLDEHPSISPKDVSSGAGGGGGIPQASVRPLVRPVIMSPEQNDRLGRHSPESKTKKSARKERMRTISEDSDGDSQPFQGGFTSTVCVSFSQDDLRLATIPGQDFDPATRRFSEEELKPQPIIRKRKKQFVPDELKNSKYWVKRSKNNEAAKRSREARRLKENQIAMRARYLEEENNVLKGEVETLKKENTDLKGMMVALEEKLSQLVENR

*Labidocera maderae* PAR-domain protein 1 I variant 2 (Labma-PDP1-I-v2)

MHRYNLIRPANIDTLKEQQVPIRPPIMHHVGKPPELYSHTMQDLDEHPSISPKDVSSGAGGGGGIPQASVRPLVRPVIMSPEQNDRLGRHSPEKRMRTISEDSDGDSQPFQGGFTSTVCVSFSQDDLRLATIPGQDFDPATRRFSEEELKPQPIIRKRKKQFVPDELKNSKYWVKRSKNNEAAKRSREARRLKENQIAMRARYLEEENNVLKGEVETLKKENTDLKGMMVALEEKLSQLVENR

*Labidocera maderae* PAR-domain protein 1 II (Labma-PDP1-II)

MTCNVDQEIYENNWPGLTLRKLMGKVDIFPGQFWNPGGMGGCIKPDKEEDKSWNNPQTAFLGPQLWDKKLSISNFEQDVSWDNYRGWNHNQVNCQGNGFMNENQAGYRNNYLNHWNQQKQEELPPKKVDDADVEFKVSDEDLSLAMVPGAEFDPKTRCFSSEELKPQPIIKKRPKIFTPINEKDDRYWEKRAKNNAAARRSREARRLKENQIALRAAYLEKQNKHLKITLKNLNLENANMKVNVDHLLARIREKENQAKSSPSPQK

*Labidocera maderae* PAR-domain protein 1 III (Labma-PDP1-III)

MADGLSGLGVSSQGMTIKDILEKVDLFNICVEEDAQMKDKNLMKNSEGKTVIQVSDPRSAYLGPKLWDKQISLSLDLEADHSNDVMNMEEFLAENNIDLDMLEKKEVNDNSNNSFESMDVSTPQGLWGGMESPQSPEIQDTKPVIRPNVIMGGPKKGNEEAGVKKNLNSSSLPKGDNMFLYAESKRARIEREKEERRRKLEEQVDFAPEDLALATIPGASFNPKERAFDMEELRPQPIIRKRKKTYVPDEAKDEKYWENRIKNNVAARRSREARRLKENQIALRAAYLEKENKLLKQELEDVEFLNSKLVTERDILKMKLAKYESMAPQ

*Labidocera maderae* PAR-domain protein 1 IV (Labma-PDP1-IV)

MEVQGGLMSNGMIKELLDRLDVNTNMNTSPPLKAKVEVESIPAPESAYLGPKLWQKPISLQQINEDDFFVLNIDDFLAENDISKDKFGAALKENEKSPEPDEMRCTNMANICLKNPSSPEMMVMSPGGSIPSPISVPSPRPGVIVSMKDCKKNVLPKGDNGFLYAESKRAKMEREKEEKKRRMEMDMEFAPEDLALATVPGADFDPRERAFDVEELRPQPIIRKRPKMFVSETAKDDKYWEKRSKNNVAARRSREARRLKENQIALRAAFLEKENNVLKNELDDANFENSKLAMERDILKKKLAKYESMSSI

*Labidocera maderae* protein phosphatase 1 I (Labma-PP1-I)

MAEGEINIDNIIQRLLEVRGSRPGKTVQMSEAEVRGLCLKSRELFLQQPILLELEAPLKICGDIHGQYTDLLRLFEYGGFPPEANYLFLGDYVDRGKQSLETICLLLAYKIKYPENFFLLRGNHECASINRIYGFYDECKRRYNIKLWKTFTDCFNCLPIAAIVDEKIFCCHGGLSPDLQSMEQIRRIMRPTDVPDTGLLCDLLWSDPDKDVQGWGENDRGVSFTFGADVVSKFLNRHDLDLICRAHQVVEDGYEFFAKRQLVTLFSAPNYCGEFDNAGGMMSVDETLMCSFQILKPSEKKAKYQYQGLNRPQTPRGPQNNPGNIKRK

*Labidocera maderae* protein phosphatase 1 II (Labma-PP1-II)

MDAKFGGPGGGGKETDKLNIDSIIARLLEVRGSRPGKNVQLSEYEIRGLCLKSRELFLSQPILLELEAPLKICGDVHGQYYDLLRLFEYGGFPPESNYLFLGDYVDRGKQSLETVCLLLAYKIKYPENFFLLRGNHECASINRIYGFYDECKRRYNVKLWKTFTDCFNCLPVAAIVDEKIFCCHGGLSPDLQSMEQIRRIMRPTDVPDQGLLCDLLWSDPDKDTMGWGENDRGVSFTFGAEVVSKFLHKHDFDLICRAHQVVEDGYEFFAKRQLVTLFSAPNYCGEFDNAGAMMSVDETLMCSFQILKPADKKKFPYGGLNSSRPLTPPRGATQQKGKKK

*Labidocera maderae* protein phosphatase 1 III (Labma-PP1-III)

MGEKGDVSEVDVDSIIDRLLEVRGCRPGKQVQLDEHEIKWLCSKSREIFISQPILLELEAPIKICGDIHGQYYDLLRLFEYGGFPPEANYLFLGDYVDRGKQSLETICLLLAYKIKYPENFFILRGNHECASINRIYGFYDECKRRYNIKLWKTFTDCFNCLPIAAIIDEKIFTMHGGLSPDLQSMEQIRRVMRPTDVPDTGLLCDLLWSDPEKEITGWGENDRGVSFTFGPDVVSRFLQNHEMDLICRAHQVVEDGYEFFAKRQLVTLFSAPNYCGEFDNAGAMMSVDDTLMCSFQILKPAEKKQKYTYAAGRMG

*Labidocera maderae* protein phosphatase 1 IV (Labma-PP1-IV)

MSIEVPTSDSSAINYSTPIQDVNNVSRRPTIKNPVKPLTRMMRNLKDANGSSVPNLTTNGTSNTQSNQLIDSIISKLIDIGMSGRVPRVLPIKSSDILWLLAQVQPILLSQPTLLEIQAPVKIVGDIHGQYLDLMRIFTKSGWPPGSNYLFLGDYVDRGKQSLETILLLFAFKVRYKGRFFLLRGNHECQGICRVYGFYDECKRRASVKIWKSFVDTFNCLPIAAVVADKIFCVHGGLSPELNSMQRIRDIERPTEVPDFGLINDLLWSDPSHSAVDWEDSDRGVSYCFGRDILSRFLHKHKFDLLARAHMVVEDGYEFFANRGLVTIFSAPNYCGIFKNMGAVMIVEPDLVCRFDLLQPASQASVTLQDEVVKADMAAAHAHAQLQNKDGNGTTGDGSNVNAQDRASPGGSVLSRSLSTKGGKVLKSPGLGHKSVNGDQGVISGARWNPNESPPEGSLLITRDKSKR

*Labidocera maderae* protein phosphatase 2A catalytic subunit – microtubule star I (Labma-MTS-I)

MNQDEKTNLKELDGWVEQLMECKQLSENQVKTLCEKAKEILSKESNVQEVKCPVTVCGDVHGQFHDLMELFRIGGKSPDTNYLFMGDYVDRGYYSVETVTLLVTLKVRFRERITILRGNHESRQITQVYGFYDECLRKYGNANVWKYFTDLFDYLPLTALVDSQIFCLHGGLSPSIDTLDHIRALDRLQEVPHEGPMCDLLWSDPDDRGGWGISPRGAGYTFGQDISETFNHTNGLTLVSRAHQLVMEGYNWSHDRNVVTIFSAPNYCYRCGNQAAIMELDDALKYSFLQFDPAPRRGEPHVTRRTPDYFL

*Labidocera maderae* protein phosphatase 2A catalytic subunit – microtubule star II (Labma-MTS-II)

MQTGGGSTVVGAPMQGFRQEPPHGSPTQFTPATASNPPTAMHSPSPEVDEVDRWVAQLSDCKQLSELEVKKLCDKAREILVTESNVQPVRCPITVCGDIHGQFHDLMELFRIGGNCPDTNYLFMGDYVDRGYYSVETVTLLVTLKVRYRDRVTILRGNHESRQITQVYGFYDECLRKYGNANVWKYFTDLFDYLPLTALIEEQVFCLHGGLSPSIDNLDHIRCLDRIQEVPHEGPMCDLLWSDPDDRPGWGISPRGAGYTFGQDISEAFNHNNSLTLIARAHQLVMEGYNWCQDRNVVTIFSAPNYCYRCGNQASIMEIDEHMKYTFLQFDPAPRRGEPHVTRRTPDYFL

*Labidocera maderae* protein phosphatase 2A regulatory subunit – twins I (Labma-TWS-I)

MANPGNSDIQWSFSQVKGTLDDDVTEADIISCVEFNQDGELLATGDKGGRVVIFQRDAASKASIPRRGEYNVYSTFQSHEPEFDYLKSLEIEEKINKIRWLKRKNPAHFLLSTNDKTIKLWKVSERDRRAEGYNLKEENGTIRDPASIKSLRVPVLKPMDLMVEASPRRIFANAHTYHINSISVNSDQETYLSADDLRINLWHMEITDQSFNIVDIKPANMEELTEVITASEFHPKECNLFVYSSSKGTIRLCDMRQSALCDSHAKMFEEPEDPTNRSFFSEIISSISDVKFSNSGRYMISRDYLSVKVWDLHMESKPIETFTVHEYLRSKLCSLYENDCIFDKFECCWNGTDSAIMTGSYNNFFRMFDRSSRKEVTLEASRDVAKPKTMLKPRKVCSGGKRKKDEISVDCLDFNKKILHTAWHPQENIIAVAATNNLFLFQEKY

*Labidocera maderae* protein phosphatase 2A regulatory subunit – twins II (Labma-TWS-II)

MAVLLTDTPAAMDTSEPMHTDPPSQSLTSPIETDPRVAPPSASDAPIDDDSQPMMGLGASPAELTWKFAQCFGDKGEIEEITEADIISAVEFDHTGDYLATGDKGGRVVLFERNKSKKGCEYKFYTEFQSHEPEFDYLKSLEIEEKINKIRWVPRQNQAHFLLSTNDKTIKLWKVFEKSLKMVAETNLNPNGKKNGGGRGLILPMMQHQDTMVAAIPRRTYANAHAYHINSISLNSDGETYISADDLRINLWNLNISDQSFNIVDIKPANMEELTEVITAAEFHPQHCNLFMYSISKGAIKLADMREAALCDRYTKVFDEDEDASSKSFFTEIIASISDIKFSNDGRYILSRDYLTLKVWDMRMENKPLKTISIHDHLRSKLCDLYENDCIFDKFECTWSGDGRGLMTGSYNNFLHIFDVNGQYDCLLQADKSAFKSRKFSYGQPPSPGGTTPPAMPPGSAGMATPGMSQGNAPGTPGATGPGSNTAAAAAAFAAASSVHTLDFNKKILHASWHPRENSLAVAATNNLFIFSTL

*Labidocera maderae* protein phosphatase 2A regulatory subunit – widerborst variant 1 (Labma-WDB-v1)

MSTGTFVDRIDPFAKRQSLKKKQKKSQGSSRYRTGGDVELTALPLLKDVANSEQEDLFIRKLRQCCVGFDFMDPVADLKGKEVKRATLNEVVDYITSGRGVLTEPVYPEIIRMIACNLFRTLPPSDNPDFDPEEDDPTLEASWPHLTLVYEFFLRFLESPDFQPTIGKKVIDQKFVLQLLELFDSEDPRERDFLKTVLHRIYGKFLGLRAFIRKQINNFFLRKVETLCSGKVRFVYETEHFNGVGELLEILGSIINGFALPLKAEHKQFLVRVLIPLHKVRCLSLYHAQLAYCVVQFLEKDATLTEQVIKGLLKFWPKTCSQKEVMFLGEIEEILDVIEPAQFVKIQEPLFKQIAKCVSSPHFQVAERALYFWNNEYIMSLIEENNQVIMPIMFPALYRISKEHWNQTIVALVYNVLKTFMEMNSRLFDDLTASYKADRQKEKKKEREREDLWRKMQDLSIKNANKEGISLDMIEMNHVKD

*Labidocera maderae* protein phosphatase 2A regulatory subunit – widerborst variant 2 (Labma-WDB-v2)

MSVLTKSPGYIRKWIQSTANAEKPARGESIIDVANSEQEDLFIRKLRQCCVGFDFMDPVADLKGKEVKRATLNEVVDYITSGRGVLTEPVYPEIIRMIACNLFRTLPPSDNPDFDPEEDDPTLEASWPHLTLVYEFFLRFLESPDFQPTIGKKVIDQKFVLQLLELFDSEDPRERDFLKTVLHRIYGKFLGLRAFIRKQINNFFLRKVETLCSGKVRFVYETEHFNGVGELLEILGSIINGFALPLKAEHKQFLVRVLIPLHKVRCLSLYHAQLAYCVVQFLEKDATLTEQVIKGLLKFWPKTCSQKEVMFLGEIEEILDVIEPAQFVKIQEPLFKQIAKCVSSPHFQVAERALYFWNNEYIMSLIEENNQVIMPIMFPALYRISKEHWNQTIVALVYNVLKTFMEMNSRLFDDLTASYKADRQKEKKKEREREDLWRKMQDLSIKNANKEGISLDMIEMNHVKD

*Labidocera maderae* shaggy I (Labma-SGG-I)

MSSSRPRTTSFVEAQKSTNPNFGGMKISSKDGSKVMTVIASPGQGPDRSTEVSYTDTKVIGNGSFGVVYQAKLCETNEMVAIKKVLQDKRFKNRELQIMRRLDHCNIVSLLYFFYTSGEKKDEIYLNLVLEFIPETVYKVARQYSKQKQTIPVTFIKLYMYQLFRSLAYIHSNGICHRDIKPQNLLLDPESGILKLCDFGSAKHLVRGEPNVSYICSRYYRAPELIFGATDYTTNIDVWSAGCVFAELMLGQPIFPGDSGVDQLVEIIKVLGTPTREQIKEMNPNYTEFKFPQIKAHPWPKVFRARTSPEAIDLVSRLLEYTPSARITPLQACAHTFFDELREPSTKLPSGRDLPPLFNFTEQELKIQPSLNSQLIPSHLQTNTGESSSESPLQDSTDKQDAAMSQPSSSS

*Labidocera maderae* shaggy II variant 1 (Labma-SGG-II-v1)

MELCPYGVRACPYEEPLNPTPLMQHVPSVISPLKGSFLPPVSFNQEHSTAGTVDRLLTSPNLEHFRTLQHQSTDASHGRQYRKISTTDPPSYTPQLAHVSTGRGKNAQSNQRVHQTNNASSSSQPYSNNPTFTHQPATSASASGGTGGGGLPPNQPPQPPPSHHSQHGFTRTNMDSVTITATDAASGQPLRLTYSDVKPIGNGSFGYVYEGTVQDVQYIDDSAVQPNPSTSTTAGPPNGPSSQSSSAHPSPSPPHGPLIYVGERVAIKKVLQDKRFKNRELQIMRMMRHPNIVQLKAFFYSNGDKPTKDDVYLNLVIEFIPETIYKSSRSYTRAKQYMPFLLIKLYMYQLFRSLAYIHSLGICHRDIKPQNLLLNPLTGVLKLCDFGSAKVLVSGEPNVAYICSRYYRAPELIFGSTQYTTHIDLWSTGCVMAELMIGQPLFPGESGVDQLVEIIKILGTPTREQIRAMNQNYTEHRFPIINSHPWSRVFRVRQSTPPEGLDLIGRLLNYTPGERISAIEAVTHPFFDDLRVPETKLGNGRELPPLFDFTPRELSIRHELNRRLIPRHAEPDLAARGVDLDKYLYQSGGPSSRDRQMDGL

*Labidocera maderae* shaggy II variant 2 (Labma-SGG-II-v2)

MELCPYGEPLNPTPLMQHVPSVISPLKGSFLPPVSFNQEHSTAGTVDRLLTSPNLEHFRTLQHQSTDASHGRQYRKISTTDPPSYTPQLAHVSTGRGKNAQSNQRVHQTNNASSSSQPYSNNPTFTHQPATSASASGGTGGGGLPPNQPPQPPPSHHSQHGFTRTNMDSVTITATDAASGQPLRLTYSDVKPIGNGSFGYVYEGTVQDVQYIDDSAVQPNPSTSTTAGPPNGPSSQSSSAHPSPSPPHGPLIYVGERVAIKKVLQDKRFKNRELQIMRMMRHPNIVQLKAFFYSNGDKPTKDDVYLNLVIEFIPETIYKSSRSYTRAKQYMPFLLIKLYMYQLFRSLAYIHSLGICHRDIKPQNLLLNPLTGVLKLCDFGSAKVLVSGEPNVAYICSRYYRAPELIFGSTQYTTHIDLWSTGCVMAELMIGQPLFPGESGVDQLVEIIKILGTPTREQIRAMNQNYTEHRFPIINSHPWSRVFRVRQSTPPEGLDLIGRLLNYTPGERISAIEAVTHPFFDDLRVPETKLGNGRELPPLFDFTPRELSIRHELNRRLIPRHAEPDLAARGVDLDKYLYQSGGPSSRDRQMDGL

*Labidocera maderae* supernumerary limbs variant 1 (Labma-SLIMB-v1)

MEEMDVSSEGVSGEGLREVEEEVQHATFQTTPTRTPPSRTSPVKMSPVSEKTSPVELDAESLEPSPKYLKDREVCLEYFTCWTEQDQINFVKDLIRSMHHHQHGAVNAFLKPMLQRDFISLLPKKGLDHVAENILSFLDARSLCAAELVSKEWNRVISEGMLWKKLIERKVNTDSLWRGLAERRGWNQFLFKPKPGESHPDYSFYRKLYPSIIKDIETIEDNWRCGKHNLQRINCRSENSKGVYCLQYDDSKIVSGLRDNTIKMWDRQTLQQHQRVLTGHTGSVLCLQYDDKVIISGSSDSTVRVWDVETGEMVNTLIHHCEAVLHLSFKFRYMNGMMVTCSKDRSIAVWDMVSPTEINLRRVLVGHRAAVNVVDFDDKYIVSASGDRTIKVWSTHSCEFVRTLNGHKRGIACLQYHDRLVVSGSSDNTIRLWDIECGQCLRVLEGHEELVRCIRFDSKRIVSGAYDGKIKVWDLQAALDPRTPTGTLCTRTLVEHSGRVFRLQFDEFQIVSSSHDDTILIWDFLNCTPPERLTFQQQEPFKNVHLR

*Labidocera maderae* supernumerary limbs variant 2 (Labma-SLIMB-v2)

MEEMDVSSEGVSGEGLREVEEEVQHATFQTTPTRTPPSRTSPVKMSPVSEKTSPVELDAESLEPSPKYLKDREVCLEYFTCWTEQDQINFVKDLIRSMHHHQHGAVNAFLKPMLQRDFISLLPKKGLDHVAENILSFLDARSLCAAELVSKEWNRVISEGMLWKKLIERKVNTDSLWRGLAERRGWNQFLFKPKPGESHPDYSFYRKLYPSIIKDIETIEDNWRCGKHNLQRINCRSENSKGVYCLQYDDSKIVSGLRDNTIKMWDRQTLQQHQRVLTGHTGSVLCLQYDDKVIISGSSDSTVRVWDVETGEMVNTLIHHCEAVLHLSFKFRYMNGMMVTCSKDRSIAVWDMVSPTEINLRRVLVGHRAAVNVVDFDDKYIVSASGDRTIKVWSTHSCEFVRTLNGHKRGIACLQYHDRLVVSGSSDNTIRLWDIECGQCLRVLEGHEELVRCIRFDSKRIVSGAYDGKIKVWDLQAALDPRTPTGTLCTRTLVEHSGRVFRLQFDEFQIVSSSHDDTILIWDFLNCTPPESNKSPSRTYTYVSNF

*Labidocera maderae* vrille (Labma-VRI)

MVAGEFGSYGEREKDGQGNRSSAQGEAMMLESKFFPGHPSLGQSPYSPSFGRKDIFSQRKQREFIPENKKDDSYWDRRRRNNEAAKRSREKRRLNDMLLETRVLELTKDNHILQAQLNAIYEKYGIKGENMISMDQVLSTLPSNDQVLNFTKKRLGPIHGLGSASPNPLSFQSLNLPLNMNNNTMNNNNNTNTTPNNNTTSINNNAGINHEMRSCSPSVPHSPDRSYRSPSPHYGPTPQNYTDIRSYAETGHMYRRPFSRSTYEDEQNDDKDSGLALNLSTDRAGSDGRSSGTGSPPVITSSNIGEREGSSSTSSSDESGYPRSPNSGESSLPHKLRFKSVVNEKEAVSSLLSLHQIAMIKREPTEHINHWMERMESGGGVIPNPNFFASLLPQRTAPLAEHQQQQEEASEGPLQKRARPTPDNLTDEVARLTSEVATLKNILVNRMKDETNEGRDE

**III. Clock input pathway proteins**

*Labidocera maderae* cryptochrome 1 (Labma-CRY1)

**MTEKETVNILWFRNGLRFHDNGSLLNATKDKKAKLLPLFIFDGETPVTKQCKYNKMAFLLECLEDLHQQLKEVGSQLYCIRGKPVDVFRKLSKKLKILKLCFDQDCEPIWLERENSVKNFCASHKIEVFENVGATLWNPMDIIKGNDGSPPLTYAQFCHVTQGIGPPDHPCESIDLKTLDIVGLDEDLMAELQFFPGVPSPEDLGFKFSGEQKIYKGGERKALKFFNRRVEFEMEAFLDGSFLPNRREPDILNPPKSLSPDLKFGCLSVKKFYWAIMDAFKQVHEGNPAPSYTIVSQLIWREFFYTMSTKNPFYGEIWRNPICINVPWKNDDVLLEKFLAAKTGYPFIDAGLRQLKSEGWIHHILRNAISMFLTRGDLFLSWEHGLNLFLSYLIDADWAVCAGNWMWVSSSAFEKALNCSFSLDPRVYGRRVDPFGAYIKRYVPELKEFPVEYIYSPWTAPVEVQEKAGCIIGKDYPHPMVDHDKVVESNRQIMLDLQDNLMKELKKQPNHIKPSDDCEIRNFFRLGINED**

**IV. Clock output pathway proteins**

*Labidocera maderae* pigment dispersing hormone precursor variant 1 (Labma-prepro-PDH-v1)

**MLIRNLIFLFLYLVVQGKMVYQYRYDGRPVNPDPEMRTRLIEEDDEGRRPFQLGGNRGARGYQAPPDINDFFIPQASKLSRGINNNIGNFDGFSEYKQESRVENQGLKGKMAKKNSEMLHILRSMPKDMGKIIRNG**

*Labidocera maderae* pigment dispersing hormone precursor variant 2 (Labma-prepro-PDH-v2)

**MLIRNLIFLFLYLVVQGKMVYQYRYDGRPVNPDPEMRTRLIELGGNRGARGYQAPPDINDFFIPQASKLSRGINNNIGNFDGFSEYKQESRVENQGLKGKMAKKNSEMLHILRSMPKDMGKIIRNG**

*Labidocera maderae* pigment dispersing hormone receptor (Labma-prepro-PDHR)

**+RHLRGVIQTNFAWRTCGDGDWLDRDGIPVENNQSYTNFIQCLPRQMYVDATAFLNDSDWNKEWKGWVLDTTAYLEILGYFVSIISLLGSIFIFTFFKSLWTQEKRIHLHLYLGMFIQVFIRLMLYLEQRNTMNFTRVERIEMALSAWRGFSETEIVCHLTIILLEYSKTVMFHWMFIEGIHLNNILVVNVFLTNSDRNQNIYLFVGWLLPVLSVGVWSLSSWSTIYSQCWYGYNHEIYYWIVEGPRLFILMLNFVFLLNILRMLYTRKETGSTQDYSFVRSSIKVTIVLQPLLGISNILQIVDSPYDAGLVYFTFWSFSTSFLASFQGFFASIFYCFCNRDVHNVVKSYVRKQLFQRRYMEGVPRTESVRTRHSRVDRSQLFQEREPEHSSWSPRGRSVRLTSQPILADLSSARVNHHMERITEETML**
