# Supplementary material for: A deep transcriptomic resource for the copepod crustacean Labidocera madurae: A potential indicator species for assessing near shore ecosystem health
Source: PLoS One. 2017 Oct 24;12(10):e0186794. doi: 10.1371/journal.pone.0186794 (PMC5655441; doi:10.1371/journal.pone.0186794)
Supplement: S4 Table — For each L. madurae transcripts, transcript and protein name and Top hit results (Top hit Accession No., BLAST Score and BLAST E-value) are listed. (DOCX) [file pone.0186794.s009.docx]

| **S4 Table**. | | | | | |
| --- | --- | --- | --- | --- | --- |
| *L. madurae* protein | | Top FlyBase annotated protein hit | | | |
| Clock component | Name | Protein name | Accession No. | BLAST statistics | |
|  |  |  |  | Score | E-value |
| Core clock | Labma-CLK | Clock, isoform A | AAF50516 | 420 | 3e-117 |
|  | Labma-CRY2 | Cryptochrome 2* | ABA62409 | 889 | 0.0 |
|  | Labma-CYC-v1 | Cycle | AAF49107 | 380 | 3e-105 |
|  | Labma-CYC-v2a | Cycle | AAF49107 | 381 | 3e-105 |
|  | Labma-CYC-v2b | Cycle | AAF49107 | 380 | 4e-105 |
|  | Labma-CYC-v3 | Cycle | AAF49107 | 381 | 2e-105 |
|  | Labma-CYC-v4 | Cycle | AAF49107 | 381 | 2e-105 |
|  | Labma-PER-v1 | Period, isoform A | AAF45804 | 276 | 1e-73 |
|  | Labma-PER-v2 | Period, isoform A | AAF45804 | 276 | 1e-73 |
|  | Labma-TIM-v1 | Timeless, isoform B | AAF51098 | 446 | 1e-124 |
|  | Labma-TIM-v2 | Timeless, isoform B | AAF51098 | 446 | 1e-124 |
|  | Labma-TIM-v3 | Timeless, isoform T | NA^1^ | 446 | 1e-124 |
|  | Labma-TIM-v4 | Timeless, isoform B | AAF51098 | 446 | 1e-124 |
| Clock-associated | Labma-CKIIα | Casein kinase IIα, isoform A | AAN11415 | 571 | 5e-163 |
|  | Labma-CKIIβ | Casein kinase IIβ, isoform K | AGB95305 | 417 | 7e-117 |
|  | Labma-CWO-v1 | Clockwork orange, isoform A | AAF54527 | 131 | 4e-30 |
|  | Labma-CWO-v2 | Clockwork orange, isoform A | AAF54527 | 133 | 8e-31 |
|  | Labma-DBT-I | Discs overgrown, isoform A | AAF57110 | 547 | 8e-156 |
|  | Labma-DBT-II-v1 | Discs overgrown, isoform A | AAF57110 | 481 | 1e-135 |
|  | Labma-DBT-II-v2 | Discs overgrown, isoform A | AAF57110 | 481 | 1e-135 |
|  | Labma-DBT-III-v1 | Discs overgrown, isoform A | AAF57110 | 463 | 2e-130 |
|  | Labma-DBT-III-v2 | Discs overgrown, isoform A | AAF57110 | 463 | 3e-130 |
|  | Labma-JET | Jetlag, isoform B | ABI31287 | 128 | 9e-30 |
|  | Labma-PDP1-I-v1 | PAR-domain protein 1, isoform J | AAN12026 | 144 | 1e-34 |
|  | Labma-PDP1-I-v2 | PAR-domain protein 1, isoform J | AAN12026 | 144 | 9e-35 |
|  | Labma-PDP1-II | PAR-domain protein 1, isoform N | AGB94241 | 134 | 1e-31 |
|  | Labma-PDP1-III | PAR-domain protein 1, isoform D | AAN12025 | 130 | 2e-30 |
|  | Labma-PDP1-IV | PAR-domain protein 1, isoform L | AGB94239 | 121 | 2e-27 |
|  | Labma-PP1-I | Flapwing, isoform B | AAF46583 | 604 | 7e-173 |
|  | Labma-PP1-II | Protein phosphatase 1α at 96A, isoform A | AAF56306 | 617 | 6e-177 |
|  | Labma-PP1-III | Flapwing, isoform B | AAF46583 | 558 | 5e-159 |
|  | Labma-PP1-IV | Protein phosphatase 1α at 96A, isoform A | AAF56306 | 394 | 2e-109 |
|  | Labma-MTS-I | Microtubule star, isoform A | AAF52567 | 609 | 2e-174 |
|  | Labma-MTS-II | Microtubule star, isoform A | AAF52567 | 562 | 2e-160 |
|  | Labma-TWS-I | Twins, isoform B | AAF54499 | 804 | 0.0 |
|  | Labma-TWS-II | Twins, isoform A | AAF54498 | 515 | 7e-146 |
|  | Labma-WDB-v1 | Widerborst, isoform A | AAF56720 | 773 | 0.0 |
|  | Labma-WDB-v2 | Widerborst, isoform A | AAF56720 | 738 | 0.0 |
|  | Labma-SGG-I | Shaggy, isoform A | AAN09082 | 652 | 0.0 |
|  | Labma-SGG-II-v1 | Shaggy, isoform Q | AGB95039 | 463 | 4e-130 |
|  | Labma-SGG-II-v2 | Shaggy, isoform Q | AGB95039 | 463 | 2e-130 |
|  | Labma-SLIMB-v1 | Supernumerary limbs, isoform A | AAF55853 | 783 | 0.0 |
|  | Labma-SLIMB-v2 | Supernumerary limbs, isoform A | AAF55853 | 783 | 0.0 |
|  | Labma-VRI | Vrille, isoform C | AAN10539 | 161 | 2e-39 |
| Clock input | Labma-CRY1 | Cryptochrome* | AAX58599 | 545 | 0.0 |
| Clock output | Labma-prepro-PDH-v1 | Uncharacterized protein Dmel_CG18635 | AAF57791 | 29.3 | 1.3 |
|  | Labma-prepro-PDH-v2 | Uncharacterized protein Dmel_CG18635 | AAF57791 | 28.9 | 1.2 |
|  | Labma-PDHR | Pigment-dispersing factor receptor, isoform C | AHN59297 | 215 | 1e-55 |
| *As *Drosophila melanogaster* does not possess CRY2, reciprocal BLAST searches for this protein, as well as CRY1, were conducted using the non-redundant *Danaus plexippus* proteins present in GenBank as the target database, rather than the annotated *D. melanogaster* proteins curated in FlyBase; *D. plexippus* possesses both CRY1 and CRY2 [64].  ^1^*Drosophila melanogaster* timeless, isoform T has not been assigned a GenBank accession number; its FlyBase annotated protein number is FBpp0401565.  Protein abbreviations: CLK, clock; CRY2, cryptochrome 2; CYC, cycle; PER, period; TIM, timeless; CKIIα; casein kinase IIα; CKIIβ; casein kinase IIβ; CWO, clockwork orange; DBT, doubletime; JET, jetlag; PDP1, PAR-domain protein 1; PP1, protein phosphatase 1; MTS, microtubule star; TWS, twins; WDB, widerborst; SGG, shaggy; SLIMB, supernumerary limbs; VRI, vrille; CRY1, cryptochrome 1; PDH, pigment dispersing hormone; PDHR, pigment dispersing hormone receptor. | | | | | |
