# Supplementary material for: A deep transcriptomic resource for the copepod crustacean Labidocera madurae: A potential indicator species for assessing near shore ecosystem health
Source: PLoS One. 2017 Oct 24;12(10):e0186794. doi: 10.1371/journal.pone.0186794 (PMC5655441; doi:10.1371/journal.pone.0186794)
Supplement: S5 Table — For each L. madurae transcripts, transcript name, BLAST results (Top hit Accession No., Species, Protein name, BLAST Score and BLAST E-value) are listed. (DOCX) [file pone.0186794.s010.docx]

| **S5** **Table.** | | | | | | |
| --- | --- | --- | --- | --- | --- | --- |
| *L. madurae* protein | | Top GenBank non-redundant arthropod protein hit | | | | |
| Clock component | Name | Accession No. | Species (subphylum) | Protein name | BLAST statistics | |
|  |  |  |  |  | Score | E-value |
| Core clock | Labma-CLK | KOC70150 | *Habropoda laboriosa* (H) | Circadian locomoter output cycles protein kaput | 463 | 3e-155 |
|  | Labma-CRY2 | XP_313179 | *Anopheles gambiae* (H) | AGAP004261-PA | 889 | 0.0 |
|  | Labma-CYC-v1 | KFB45024 | *Anopheles sinensis* (H) | AGAP005655-PA-like protein | 540 | 0.0 |
|  | Labma-CYC-v2a | KFB45024 | *Anopheles sinensis* (H) | AGAP005655-PA-like protein | 544 | 0.0 |
|  | Labma-CYC-v2b | NP_001107795 | *Tribolium castaneum* (H) | Cycle | 538 | 0.0 |
|  | Labma-CYC-v3 | KFB45024 | *Anopheles sinensis* (H) | AGAP005655-PA-like protein | 541 | 0.0 |
|  | Labma-CYC-v4 | KFB45024 | *Anopheles sinensis* (H) | AGAP005655-PA-like protein | 544 | 0.0 |
|  | Labma-PER-v1 | KDR13740 | *Zootermopsis nevadensis* (H) | Period circadian protein | 368 | 1e-104 |
|  | Labma-PER-v2 | KDR13740 | *Zootermopsis nevadensis* (H) | Period circadian protein | 368 | 1e-104 |
|  | Labma-TIM-v1 | CRK93394 | *Clunio marinus* (H) | CLUMA_CG006930, isoform A | 676 | 0.0 |
|  | Labma-TIM-v2 | CRK93394 | *Clunio marinus* (H) | CLUMA_CG006930, isoform A | 677 | 0.0 |
|  | Labma-TIM-v3 | CRK93394 | *Clunio marinus* (H) | CLUMA_CG006930, isoform A | 675 | 0.0 |
|  | Labma-TIM-v4 | CRK93394 | *Clunio marinus* (H) | CLUMA_CG006930, isoform A | 677 | 0.0 |
| Clock-associated | Labma-CKIIα | AII16523 | *Paracyclopina nana* (Cr) | Casein kinase IIα | 633 | 0.0 |
|  | Labma-CKIIβ | AII16524 | *Paracyclopina nana* (Cr) | Casein kinase IIβ | 434 | 3e-162 |
|  | Labma-CWO-v1 | XP_018327706 | *Agrilus planipennis* (H) | LOC108738678, isoform X1 | 193 | 6e-53 |
|  | Labma-CWO-v2 | XP_018327706 | *Agrilus planipennis* (H) | LOC108738678, isoform X1 | 195 | 8e-54 |
|  | Labma-DBT-I | AII16522 | *Paracyclopina nana* (Cr) | Casein kinase Iε | 605 | 0.0 |
|  | Labma-DBT-II-v1 | KFM71835 | *Stegodyphus mimosarum* (Ch) | Casein kinase Iε | 532 | 0.0 |
|  | Labma-DBT-II-v2 | KFM71835 | *Stegodyphus mimosarum* (Ch) | Casein kinase Iε | 531 | 0.0 |
|  | Labma-DBT-III-v1 | KFM71835 | *Stegodyphus mimosarum* (Ch) | Casein kinase Iε | 496 | 1e-173 |
|  | Labma-DBT-III-v2 | KFM71835 | *Stegodyphus mimosarum* (Ch) | Casein kinase Iε | 496 | 1e-173 |
|  | Labma-JET | ACO15768 | *Caligus clemensi* (Cr) | F-box only protein 37 | 224 | 1e-70 |
|  | Labma-PDP1-I-v1 | XP_017490459 | *Rhagoletis zephyria* (H) | Hepatic leukemia factor | 142 | 3e-40 |
|  | Labma-PDP1-I-v2 | XP_017490459 | *Rhagoletis zephyria* (H) | Hepatic leukemia factor | 142 | 3e-40 |
|  | Labma-PDP1-II | XP_006616203 | *Apis dorsata* (H) | hepatic leukemia factor-like isoform X7 | 137 | 4e-37 |
|  | Labma-PDP1-III | ACO11939 | *Lepeophtheirus salmonis* (Cr) | Hepatic leukemia factor | 182 | 3e-53 |
|  | Labma-PDP1-IV | ACO11939 | *Lepeophtheirus salmonis* (Cr) | Hepatic leukemia factor | 164 | 2e-46 |
|  | Labma-PP1-I | XP_008192788 | *Tribolium castaneum* (H) | Serine/threonine-protein phosphatase PP1β catalytic subunit isoform X2 | 635 | 0.0 |
|  | Labma-PP1-II | JAN79021 | *Daphnia magna* (Cr) | Serine/threonine-protein phosphatase PP1β catalytic subunit | 654 | 0.0 |
|  | Labma-PP1-III | XP_015905891 | *Parasteatoda tepidariorum* (Ch) | Serine/threonine-protein phosphatase PP1β catalytic subunit | 574 | 0.0 |
|  | Labma-PP1-IV | ACO15366 | *Caligus clemensi* (Cr) | Serine/threonine-protein phosphatase PP1β | 398 | 3e-135 |
|  | Labma-MTS-I | KMQ93778 | *Lasius niger* (H) | Serine threonine-protein phosphatase 2A catalytic subunit α | 627 | 0.0 |
|  | Labma-MTS-II | KMQ93778 | *Lasius niger* (H) | Serine threonine-protein phosphatase 2A catalytic subunit α | 583 | 0.0 |
|  | Labma-TWS-I | XP_008207502 | *Nasonia vitripennis* (H) | Protein phosphatase PP2A 55 kDa regulatory subunit isoform X5 | 847 | 0.0 |
|  | Labma-TWS-II | XP_011567020 | *Plutella xylostella* (H) | Protein phosphatase PP2A regulatory subunit B-like | 647 | 0.0 |
|  | Labma-WDB-v1 | KPI92290 | *Papilio xuthus* (H) | Serine/threonine-protein phosphatase 2A 56 kDa regulatory subunit α | 855 | 0.0 |
|  | Labma-WDB-v2 | KPI92290 | *Papilio xuthus* (H) | Serine/threonine-protein phosphatase 2A 56 kDa regulatory subunit α | 789 | 0.0 |
|  | Labma-SGG-I | JAS06994 | *Clastoptera arizonana* (H) | Hypothetical protein g.17724 | 692 | 0.0 |
|  | Labma-SGG-II-v1 | JAS06994 | *Clastoptera arizonana* (H) | Hypothetical protein g.17724 | 499 | 2e-171 |
|  | Labma-SGG-II-v2 | JAS06994 | *Clastoptera arizonana* (H) | Hypothetical protein g.17724 | 499 | 9e-172 |
|  | Labma-SLIMB-v1 | KDR19729 | *Zootermopsis nevadensis* (H) | F-box/WD repeat-containing protein 1A | 824 | 0.0 |
|  | Labma-SLIMB-v2 | XP_013784678 | *Limulus polyphemus* (Ch) | β-TrCP-like | 828 | 0.0 |
|  | Labma-VRI | XP_014273353 | *Halyomorpha halys* (H) | Nuclear factor interleukin-3-regulated protein | 191 | 5e-54 |
| Clock input | Labma-CRY1 | BAF45421 | *Dianemobius nigrofasciatus* (H) | Cryptochrome | 551 | 0.0 |
| Clock output | Labma-prepro-PDH-v1 | XP_011496884 | *Ceratosolen solmsi marchali* (H) | Peptidyl-α-hydroxyglycine α-amidating lyase 1 | 32.3 | 9.1 |
|  | Labma-prepro-PDH-v2 | KNC30537 | *Lucilia cuprina* (H) | Hypothetical protein FF38_02282 | 36.2 | 0.35 |
|  | Labma-PDHR | XP_013136553 | *Papilio polytes* (H) | PDF receptor | 247 | 9e-75 |
| Protein abbreviations: CLK, clock; CRY2, cryptochrome 2; CYC, cycle; PER, period; TIM, timeless; CKIIα; casein kinase IIα; CKIIβ; casein kinase IIβ; CWO, clockwork orange; DBT, doubletime; JET, jetlag; PDP1, PAR-domain protein 1; PP1, protein phosphatase 1; MTS, microtubule star; TWS, twins; WDB, widerborst; SGG, shaggy; SLIMB, supernumerary limbs; VRI, vrille; CRY1, cryptochrome 1; PDH, pigment dispersing hormone; PDHR, pigment dispersing hormone receptor.  Arthropod subphylum abbreviations: Ch, Chelicerata; Cr, Crustacea; H, Hexapoda. | | | | | | |
