# Supplementary material for: A deep transcriptomic resource for the copepod crustacean Labidocera madurae: A potential indicator species for assessing near shore ecosystem health
Source: PLoS One. 2017 Oct 24;12(10):e0186794. doi: 10.1371/journal.pone.0186794 (PMC5655441; doi:10.1371/journal.pone.0186794)
Supplement: S6 Table — (DOCX) [file pone.0186794.s011.docx]

| **S6 Table.** | | |
| --- | --- | --- |
| *L. madurae* protein | | Pfam domains/regions (amino acid coordinates*) |
| Clock component | Name |  |
| Core clock | Labma-CLK | Helix-loop-helix DNA-binding domain (23-73); PAS domain (277-385) |
|  | Labma-CRY2 | DNA photolyase (11-175); FAD binding domain of DNA photolyase (291-491) |
|  | Labma-CYC-v1 | Helix-loop-helix DNA-binding domain (94-147); PAS fold (167-236); PAS domain (378-487) |
|  | Labma-CYC-v2a | Helix-loop-helix DNA-binding domain (88-141); PAS fold (161-230); PAS domain (372-481) |
|  | Labma-CYC-v2b | Helix-loop-helix DNA-binding domain (88-141); PAS fold (161-230); PAS domain (372-481) |
|  | Labma-CYC-v3 | Helix-loop-helix DNA-binding domain (57-110); PAS fold (130-200); PAS domain (341-450) |
|  | Labma-CYC-v4 | Helix-loop-helix DNA-binding domain (51-104); PAS fold (124-194); PAS domain (335-444) |
|  | Labma-PER-v1 | PAS fold (411-516); Period protein 2/3C-terminal region (1049-1232) |
|  | Labma-PER-v2 | PAS fold (407-512); Period protein 2/3C-terminal region (1045-1228) |
|  | Labma-TIM-v1 | Timeless protein (20-289) |
|  | Labma-TIM-v2 | Timeless protein (20-285) |
|  | Labma-TIM-v3 | Timeless protein (20-289) |
|  | Labma-TIM-v4 | Timeless protein (20-285) |
| Clock-associated | Labma-CKIIα | Protein kinase domain (37-322) |
|  | Labma-CKIIβ | Casein kinase II regulatory subunit (8-191) |
|  | Labma-CWO-v1 | Helix-loop-helix DNA-binding domain (154-207); Hairy Orange (241-282) |
|  | Labma-CWO-v2 | Helix-loop-helix DNA-binding domain (154-207); Hairy Orange (235-276) |
|  | Labma-DBT-I | Protein kinase domain (9-264) |
|  | Labma-DBT-II-v1 | Protein kinase domain (137-398) |
|  | Labma-DBT-II-v2 | Protein kinase domain (137-399) |
|  | Labma-DBT-III-v1 | Protein kinase domain (7-257) |
|  | Labma-DBT-III-v2 | Protein kinase domain (7-257) |
|  | Labma-JET | Leucine Rich repeat (210-233) |
|  | Labma-PDP1-I-v1 | Basic region leucine zipper (177-230) |
|  | Labma-PDP1-I-v2 | Basic region leucine zipper (168-221) |
|  | Labma-PDP1-II | Basic region leucine zipper (183-236) |
|  | Labma-PDP1-III | Basic region leucine zipper (252-303) |
|  | Labma-PDP1-IV | Basic region leucine zipper (235-288) |
|  | Labma-PP1-I | Serine-threonine protein phosphatase N-terminal domain (8-55); Calcineurin-like phosphoesterase (56-251) |
|  | Labma-PP1-II | Serine-threonine protein phosphatase N-terminal domain (20-67); Calcineurin-like phosphoesterase (68-263) |
|  | Labma-PP1-III | Serine-threonine protein phosphatase N-terminal domain (12-59); Calcineurin-like phosphoesterase (60-255) |
|  | Labma-PP1-IV | Serine-threonine protein phosphatase N-terminal domain (70-118); Calcineurin-like phosphoesterase (119-314) |
|  | Labma-MTS-I | Calcineurin-like phosphoesterase (52-247) |
|  | Labma-MTS-II | Calcineurin-like phosphoesterase (91-286) |
|  | Labma-TWS-I | None detected |
|  | Labma-TWS-II | None detected |
|  | Labma-WDB-v1 | Protein phosphatase 2A regulatory B subunit-B56 family (43-460) |
|  | Labma-WDB-v2 | Protein phosphatase 2A regulatory B subunit-B56 family (28-444) |
|  | Labma-SGG-I | Protein kinase domain (54-338) |
|  | Labma-SGG-II-v1 | Protein kinase domain (259-527) |
|  | Labma-SGG-II-v2 | Protein kinase domain (252-520) |
|  | Labma-SLIMB-v1 | D domain of β-TrCP (77-115); F-box-like (121-169); WD domain, G-beta repeat (229-266); WD domain, G-beta repeat (271-307); WD domain, G-beta repeat (358-394); WD domain, G-beta repeat (398-434); WD domain, G-beta repeat (438-474); WD domain, G-beta repeat (488-523) |
|  | Labma-SLIMB-v2 | D domain of β-TrCP (77-115); F-box-like (121-169); WD domain, G-beta repeat (229-266); WD domain, G-beta repeat (271-307); WD domain, G-beta repeat (358-394); WD domain, G-beta repeat (398-434); WD domain, G-beta repeat (438-474); WD domain, G-beta repeat (488-523) |
|  | Labma-VRI | Basic region leucine zipper (70-121) |
| Clock input | Labma-CRY1 | DNA photolyase (8-171); FAD binding domain of DNA photolyase (294-496) |
| Clock output | Labma-prepro-PDH-v1 | None detected |
|  | Labma-prepro-PDH-v2 | None detected |
|  | Labma-PDHR | Secretin family 7-transmembrane receptor (68-331) |
| *Amino acid coordinates presented represent the Pfam domain “Envelope”.  Protein abbreviations: CLK, clock; CRY2, cryptochrome 2; CYC, cycle; PER, period; TIM, timeless; CKIIα; casein kinase IIα; CKIIβ; casein kinase IIβ; CWO, clockwork orange; DBT, doubletime; JET, jetlag; PDP1, PAR-domain protein 1; PP1, protein phosphatase 1; MTS, microtubule star; TWS, twins; WDB, widerborst; SGG, shaggy; SLIMB, supernumerary limbs; VRI, vrille; CRY1, cryptochrome 1; PDH, pigment dispersing hormone; PDHR, pigment dispersing hormone receptor. | | |
